# Enhancing red blood cell compatibility: in vitro hemagglutination prevention using a trispecific triabody as a blocking fragment for blood group antigens

**DOI:** 10.1186/s13036-026-00661-w

**Published:** 2026-03-13

**Authors:** Saleha Hafeez, Muhammad Asghar

**Affiliations:** 1 Department of Biomedicine, Atta-Ur-Rahman School of Applied Biosciences, National University of Sciences and Technology, H-12 Sector, Islamabad, 44000 Pakistan; 2 Department of Biomedical Engineering, School of Mechanical and Manufacturing Engineering, National University of Sciences & Technology, H-12 Sector, Islamabad, 44000 Pakistan; 3 Department of Biology, Lund University, Lund, Sweden; 4 Department of Sports Sciences and Clinical Biomechanics, University of Southern Denmark, Odense, Denmark

**Keywords:** Trispecific triabody, Universal red blood cells, Binding cooperativity, Antigen blocking, Hemagglutination, Transfusion compatibility, ELISA

## Abstract

**Background:**

Access to safe and timely blood transfusion is a cornerstone of modern healthcare but depends on a stable supply of voluntary donations and rigorous hemovigilance systems. O-negative red blood cells are universally compatible and essential for emergency transfusions; however, their scarcity, particularly in low-resource regions, poses significant challenges. To address this challenge, a compact trispecific triabody was designed to block A, B, and Rh(D) antigens on RBCs.

**Results:**

In this study, two triabody configurations differing in the placement of the anti-Rh(D) variable domain were generated, producing closed (C1) and open (O1) formats. The selected triabody-C1 was expressed in *Escherichia coli* BL21(DE3) and purified into two fractions, AE3-B1 and AE3-B2. Hemagglutination assays demonstrated that AE3-B2 did not induce hemagglutination, whereas AE3-B1 showed mixed-field hemagglutination under standard conditions and complete hemagglutination under potentiator-enhanced hemagglutination conditions. ELISA-based binding assays indicated that the triabody’s monomers functioned independently with free antigens, while RBC-bound antigen assays revealed altered binding behavior upon sequential antigen engagement. Blood incompatibility related hemagglutination assays using monoclonal antibodies and incompatible O-negative blood plasma demonstrated complete prevention of hemagglutination by AE3-B2 triabody-coated RBCs, confirming effective antigen blocking.

**Conclusions:**

The trispecific triabody efficiently prevents hemagglutination by blocking A, B, and Rh(D) antigens on RBCs, enabling them to exhibit enhanced compatibility and hemagglutinating patterns similar to O-negative cells. These findings provide a promising strategy to increase the pool of compatible blood for transfusion, particularly in emergency and resource-limited settings, while emphasizing that future in vivo investigations are needed to confirm efficacy and safety.

**Graphical Abstract:**

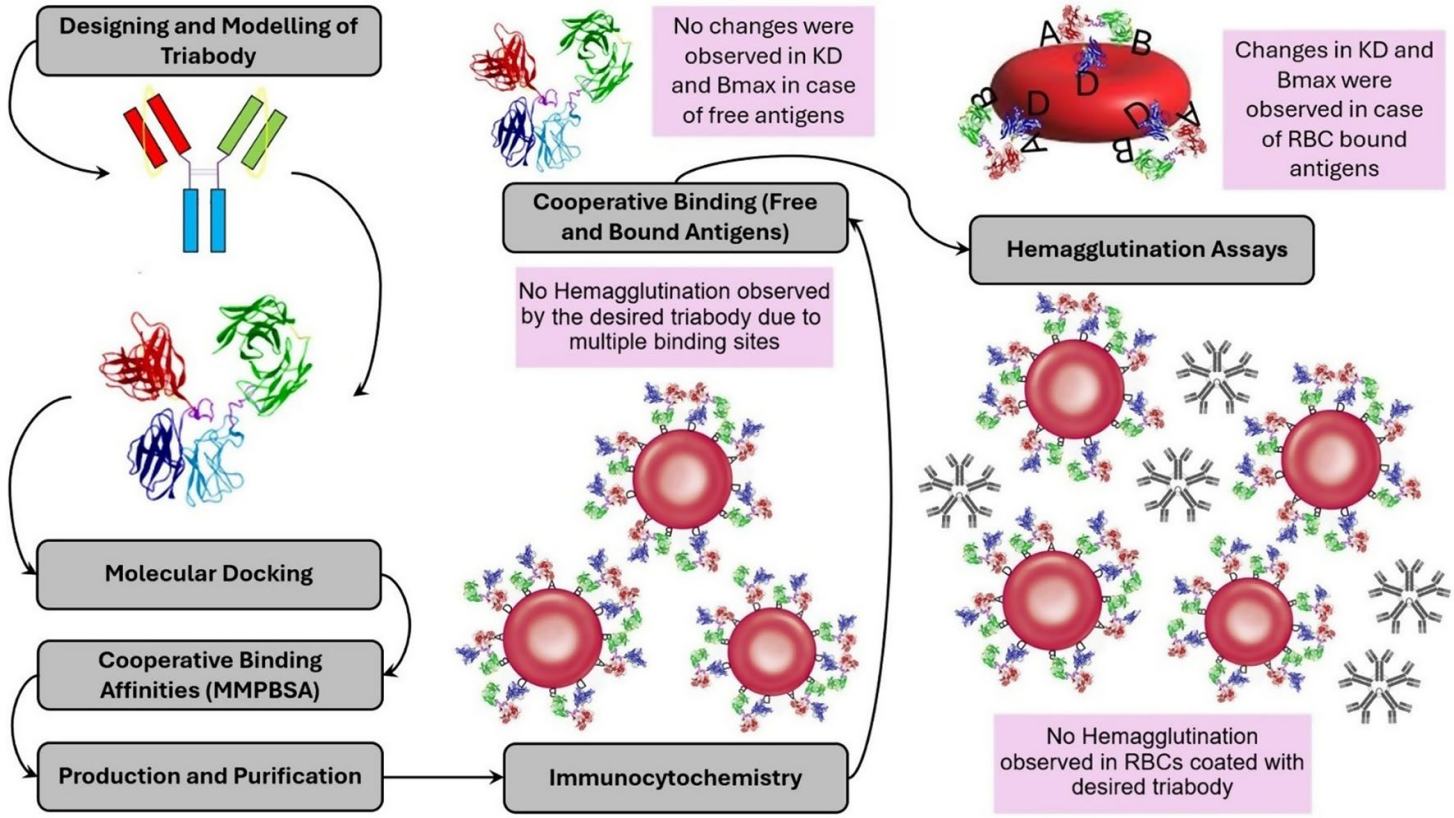

**Supplementary Information:**

The online version contains supplementary material available at 10.1186/s13036-026-00661-w.

## Introduction

Transfusion medicine has long been an integral part of modern medicine, which relies heavily on blood products received from voluntary blood donors. A blood transfusion frequently represents the difference between life and death during medical emergencies, critical surgical procedures, and for patients with long-term medical conditions. A critical factor in achieving a successful transfusion is ensuring blood type compatibility, as mismatches can elicit severe and potentially fatal immune responses [1]. This compatibility is governed by the presence of specific antigens on the surface of red blood cells (RBCs) and the corresponding antibodies found in the plasma [2]. RBCs clump together in the presence of these antibodies through a process called agglutination [3].

The effectiveness of hemagglutination mainly depends on the type of antibody involved i.e., immunoglobulin IgG or IgM. The IgM antibody, a large pentameric antibody, is capable of binding ten antigens [4]. Its multivalency and considerable size make it highly effective at bridging the distance between multiple RBCs. In particular, its large molecular structure enables it to overcome the natural repulsive forces that keep RBCs apart. The anti-A and anti-B antibodies belonging to the major ABO blood group system are typically IgM. Consequently, transfusions related to ABO blood type incompatibility leads to a rapid and immediate hemagglutination reaction, which can result in a life-threatening acute hemolytic transfusion reaction [5, 6].

In contrast, IgG antibodies are smaller and bivalent (able to bind only two antigens) in nature. Due to their compact size, IgG antibodies can sensitize the RBCs but are inefficient at bridging the gap between RBCs to overcome the negative zeta potential [6, 7]. Consequently, IgG-mediated hemagglutination is not directly visible and requires specialized techniques such as antiglobulin testing, also known as the Coombs test, which utilizes anti-human antibodies (secondary antibodies) to enable visible clumping of cells [8]. Antibodies belonging to the Rh blood group system are predominantly IgG in nature. An initial exposure of an Rh(D)-negative individual to Rh(D)-positive blood can lead to the development of anti-Rh(D) antibodies. A subsequent blood transfusion of Rh(D)-positive blood can lead to a delayed hemolytic transfusion reaction [9].

The combination of the ABO and Rh blood group systems gives us various blood types. Among these, O-negative RBCs hold a special position as “universal donors” due to the absence of A, B and Rh(D) antigens. The lack of immunogenic antigens makes them invaluable in time-sensitive situations where patient’s blood type is unknown, or their true blood type has been masked by a recent blood transfusion [10–12]. Despite their indispensable role, the supply of O-negative blood is limited and often faces significant depletion due to the constant high demand. This persistent need for a constant supply of blood highlights the need for readily available universally compatible blood [13, 14]. This challenge is more prominent and acute in blood deserts. Blood deserts are geographical regions where 75% of blood transfusion cases are deprived of timely and affordable blood components [15].

The urgent requirement for universal blood has driven the research into alternative solutions, including the development of blood substitutes such as perfluorocarbons (PFCs), hemoglobin-based oxygen carriers HBOCs and RBC substitutes [16, 17], enzymatic conversion of A and B blood types to O type blood [18], masking or blocking the surface antigens to increase compatibility [13] and the generation of cultured RBCs from stem cells [19]. While these strategies hold promise, no such alternative solution has been approved for blood transfusion yet.

Given the advantages of O-negative blood and its persistent demand, the present study introduces a uniquely designed trispecific triabody as a blocking fragment to address situations where immediate cross-matching is not available and to increase the pool of usable blood for emergency use. Building upon previous antigen blocking strategies [13, 20], our research introduces a uniquely designed trispecific triabody to block the A, B and Rh(D) blood group antigens. In addition to its blocking capabilities, the triabody’s small and compact structure allows it to efficiently block antigens on RBCs without inducing hemagglutination, providing a safe approach to enhance their compatibility. This could drastically improve the outcomes in underserved regions by providing readily available blood products, especially packed red blood cells (pRBCs) for transfusion when compatible blood is unavailable.

Here, we computationally designed and modeled all possible structures of the triabody. Molecular docking was performed between the selected structure of the triabody and blood group antigens A, B and Rh(D), followed by computational prediction of intramolecular cooperative binding affinities. Next, triabody was produced from *E.coli* BL21(DE3) using a two-plasmid system and purified through a three-step process. The standard hemagglutination and potentiator-enhanced hemagglutination (PEH) assay techniques were then performed to microscopically determine its hemagglutination capabilities. To determine the binding behavior, ELISA-based experiments were conducted using both free and RBC-bound antigens. Finally, RBC-bound antigens were blocked by the triabody, and blood incompatibility related hemagglutination assays were performed to evaluate its effectiveness as a blocking fragment in preventing RBC hemagglutination.

## Materials and methods

### Software, web servers and chemicals

All the software, web servers, chemicals and molecular biology products used in this study are mentioned in the supplementary materials.

### Protein designing, modeling and refinement

**Fig. 1:**
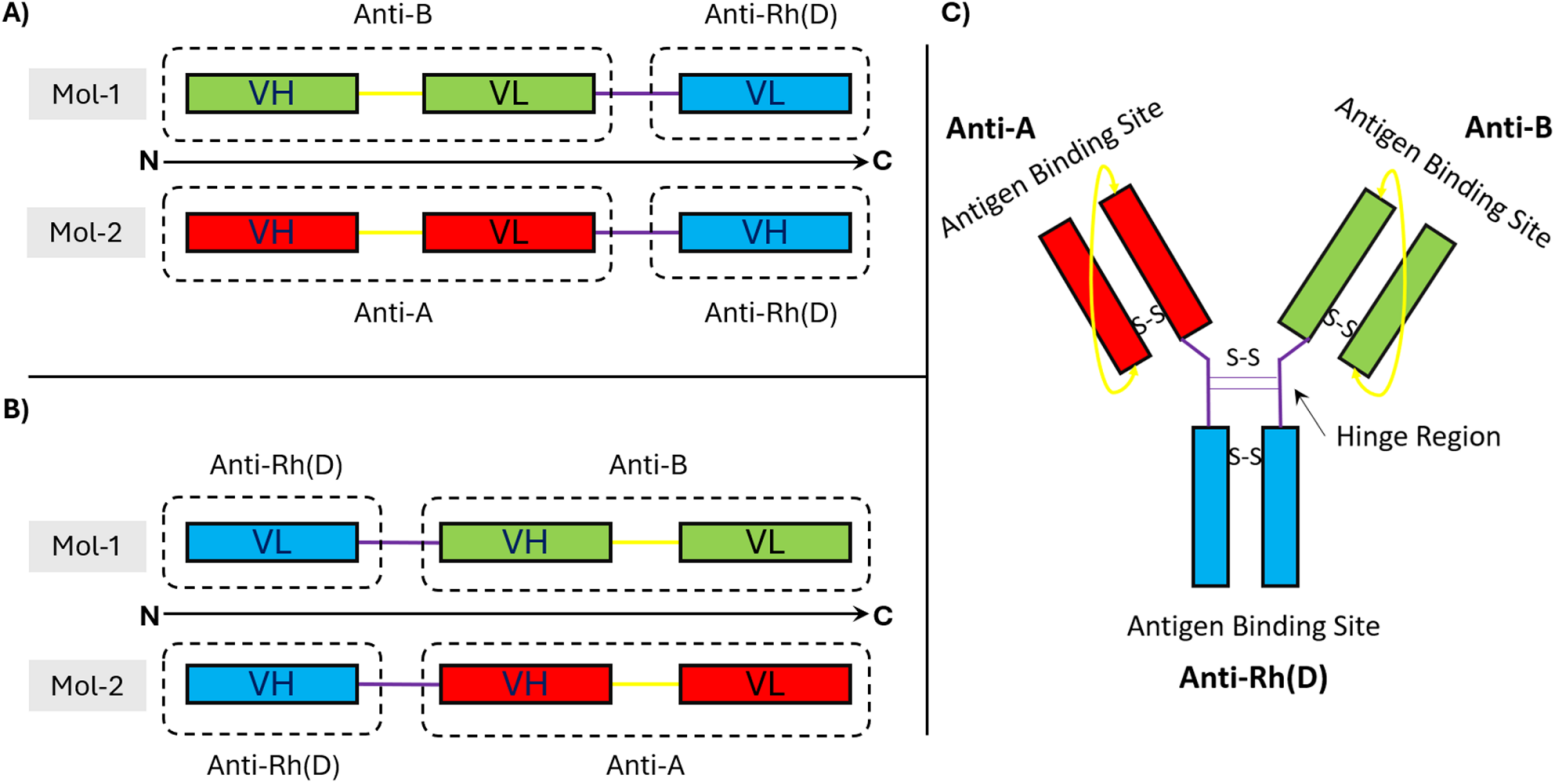
Design of the triabody. Two combinations were used for designing a triabody from two ds-scFvs of anti-A, and anti-B and dsFv of anti-Rh(D). (**A**) In the first combination, VH and VL chains of anti-Rh(D) were placed at the C-terminal region of fusion proteins and (**B**) in the second combination these chains were placed at the N-terminal region of fusion proteins. (**C**) Final design of triabody required from the two possible combinations of fusion proteins

The sequences of anti-A and anti-B fragment variables (Fvs) were taken from PDB: 1JV5 [21], and research article published by Santos-Esteban and Curiel‐Quesada (2001) [22] respectively. The sequences of anti-Rh(D) variable light (VL) and heavy (VH) chains were taken from accession numbers AAC13488.1 (anti-Rh(D) VL chain) and AAC13447.1 (anti-Rh(D) VH chain) respectively [23]. Both chains of anti-A and anti-B Fvs were joined from VH to VL via a (G_4_S)_3_ flexible linker (VH-Linker-VL). Interdomain disulfide bonds were added into each Fv at the site described by Hafeez and Zaidi (2024) [13] and Zhao et al. (2010) [24]. After the addition of disulfide bonds, the sequences were arranged into two combinations with two fusion proteins in each, as shown in detail in Fig. 1. The complete disulfide-stabilized single chain fragment variable (ds-scFv) in each fusion protein of both combinations was joined to the variable chain of the other Fv through the hinge region (EPKSCDKTHTCPPCPAPELLGGP) of the IgG1 antibody [25]. All proteins were modeled through the I-TASSER web server [26]. Models with correct domain locations and orientations of VH-VL were joined together through molecular docking using LZerD web server [27] to form a triabody. Best structures were selected for structural refinement on the basis of the accessibility of the binding site, i.e., all structures in which the binding site was not easily accessible for interaction were discarded. PyMOL 3.1 was used to visualize all the structures [28]. Theoretical molecular weights were predicted through the ProtParam tool [29].

The selected structures were protonated to pH 7.4 using the ProteinPrepare tool of the PlayMolecule web server [30]. The protonation states of amino acid residues (histidine, aspartic acid, and glutamic acid) with pKa values close to pH 7.4 were manually assigned. Intramolecular disulfide bonds were preserved while assigning protonation states. For intermolecular disulfide bonds, hydrogen atoms were removed from sulfur group of cysteine residues and bonds were formed via pdb2gmx with (-ss) and (-merge all) flags on GROMACS 2024.2 software [31]. Molecular dynamics (MD) simulations were performed to refine the structure and investigate the structural dynamics of the triabodies. Simulations were performed for 150 ns using the Amber force field ff99SB at a temperature of 298 K. The simulation results were evaluated based on the radius of gyration (R_g_) and the distance between antigen binding sites, while MD trajectories were analyzed to assess overall structural changes during the simulations.

### Modeling of antigens, molecular docking and MD simulations

The structures of trisaccharide antigens (A-trisaccharide and B-trisaccharide) were built using the Glycam web server [32], while the antigen Rh(D) sequence from Conroy et al. (2005) [33] was modeled using I-TASSER. Trisaccharide-triabody docking was performed with SwissDock [34], using grids centered on the respective antigen binding sites. For protein-protein docking, antigen Rh(D) was protonated at pH 7.4 and docked using LZerD. Best docked complexes were selected based on terminal monosaccharide interactions (trisaccharides) or complex location (protein-protein). The MD simulations were conducted in GROMACS 2024.2 for 150 ns under physiological conditions (310 K, 0.154 M NaCl, and pH 7.4). The Amber ff99SB force field was used for proteins, and Glycam-06 h topology for trisaccharides was generated using ACPYPE (AmberTools24) [35].

### Binding free energy calculations

Molecular Mechanics Poisson-Boltzmann Surface Area (MMPBSA) calculations were performed to estimate the binding free energy (ΔG_Bind_) of the triabody-antigen complexes using the MmPbAaStat.py script within the g_mmpbsa software tool [36]. The final 10 ns of MD simulations for each monomer within the triabody was considered the equilibrium phase and was used for energy calculations. To computationally predict the intramolecular cooperative binding affinities, blood group antigens A, B and Rh(D) were sequentially docked in all possible orders: A→B→Rh(D), A→Rh(D)→B, B→A→Rh(D), B→Rh(D)→A, Rh(D)→A→B and Rh(D)→B→A. MD simulations were performed after each docking step as mentioned above in Section “Modeling of antigens, molecular docking and MD simulations”. Binding free energies were then calculated through MMPBSA to determine a trend in cooperative binding affinities.

### Production and purification

The triabody molecule was expressed using two expression plasmids, pET-28a(+) and pET-21(+), which harbor kanamycin and ampicillin resistance genes, respectively. The designs of both recombinant fusion protein constructs are shown in the Supplementary Figure S1, and detailed information on the gene construct design is also provided in the Supplementary Materials. Both recombinant plasmids were obtained from Twist Bioscience (USA) and were subsequently co-transformed into *E. coli* BL21(DE3) using the heat-shock method [37].

For large-scale production, a 100 ml overnight culture of *E.coli* BL21 (DE3) was used to inoculate 5 L of LB media supplemented with (25 µg/ml) kanamycin, (50 µg/ml) ampicillin and (1%) glucose. Incubation was performed at 37 °C under constant shaking at 200 rpm until an OD_600_ of 0.6 was reached. The culture was cooled to 18 °C before slow induction was carried out with 0.25 mM IPTG at 18 °C for 20 h. The cells were harvested following induction and total proteins were extracted via sonication as described by Hafeez and Zaidi (2024) [13]. The crude extract was collected and filtered through a 0.45 μm syringe filter. Western blotting was performed for the analysis of protein samples.

A trispecific triabody was purified via a three-step process. Initially, nickel affinity chromatography (Ni^2+^-NTA) was used to isolate the his-tagged triabodies. This was followed by extraction of different-sized triabodies from the Native PAGE gel and finally by acid-glycine elution to separate the functional triabodies. Final product purity and homogeneity were assessed by size-exclusion chromatography (SEC). The presence or absence of interdomain disulfide bonds was evaluated by non-reducing SDS-PAGE. Detailed protocols for the purification techniques and SEC are provided in the Supplementary Materials.

### Hemagglutination assay and immunocytochemistry (ICC)

Immunocytochemistry (ICC) was performed to microscopically assess the ability of the triabody to induce hemagglutination of RBCs (detailed protocol is provided in the Supplementary Materials). Prior to ICC, RBCs from AB+, AB−, A+, and B+ blood types were visually screened for hemagglutination. An additional PEH assay (detailed protocol provided in the Supplementary Materials) was performed to further confirm the triabody’s ability to induce hemagglutination. Any triabody fraction that caused hemagglutination was excluded from further studies.

### Determination of intramolecular binding cooperativity of the triabody via free blood group antigens

Intramolecular binding cooperativity of the triabody was further investigated through specialized ELISA-based experiments. The aim was to understand how the binding of one antigen affects the binding of other antigens when the antigens are free. The experimental sequence began with immobilizing anti-his tag IgG antibodies (10 µg/ml) on protein A/G precoated plates, followed by the addition of purified his-tagged triabodies in twofold serial dilutions ranging from 0.2 nM to 100 µM. In studying the binding order A→B→Rh(D), the first binding event involved saturating the A binding sites with the A-trisaccharide, followed by saturation of the B binding sites with the B-trisaccharide (second binding event), and finally, the Rh(D) binding site was saturated with the antigen Rh(D) (third binding event). An overview of the process is given in Supplementary Figure S2. The protocols for determining the optimal concentrations of A-trisaccharide, B-trisaccharide, and antigen Rh(D) used to assess the binding cooperativity of the triabody are provided in Supplementary Figure S3 and Supplementary Table S1.

The binding parameters, including the dissociation constant K_D_​ and maximal binding B_max_ of the B and Rh(D) binding sites were determined via indirect ELISA as described by Syedbasha et al. (2016) [38] using unbound B-trisaccharide and the antigen Rh(D). To determine the changes in the binding parameters of the B binding site, a neoglycoconjugation process was used in which unbound B-trisaccharide was covalently coupled to bovine serum albumin (BSA) via a homobifunctional ethylenediamine (EDA) linker. This procedure proceeded with the coating of BSA (20 µg/ml dissolved in 0.1 M carbonate-bicarbonate buffer pH 9.6) onto 96-well plates overnight at 4 °C. The next day, wells were washed several times with 200 µl of 0.1 M 2-(N-morpholino) ethanesulfonic acid (MES) buffer (pH 6). A freshly prepared solution of EDC and Sulfo-NHS (5 mM each, 1:1 molar ratio) in 0.1 M MES buffer was then added (100 µl per well) and incubated for 30 min at room temperature with mild shaking. Following incubation, the wells were washed three times with 0.1 M MES buffer (pH 6).

For the coupling of EDA to BSA, an excess concentration of EDA (100 mM) was prepared in 0.1 M MES buffer (pH 6). Following the washing step, 200 µl of EDA solution was added to each well and incubated overnight at 4 °C. Immediately after incubation, the wells were washed three times with a 0.5 M solution of sodium borate Na_2_B_4_O_7_ (pH 8.5).

A reductive amination reaction was performed to facilitate the attachment of B-trisaccharide to the BSA-EDA conjugates. The plates were incubated for 96 h at 56 °C with 100 µl of the unbound B-trisaccharide solution (from second binding). Simultaneously, a 50 µl solution, composed of 0.5 M sodium borate Na_2_B_4_O_7_ (pH 9), 1.5 M sodium sulfate Na_2_SO_4_, and 0.75 M sodium cyanoborohydride NaBH_3_CN was added. Following incubation, the wells were washed rigorously three times with distilled water. An indirect ELISA was performed in which 100 µL of 10 mg/ml his-tagged triabody was used to detect B-trisaccharide. This was followed by sequential incubation with 100 µl of primary anti-his tagged (1:1000 dilution) and secondary HRP-conjugated antibodies (1:10000 dilution). The TMB substrate reaction was performed for 15 min and stopped with 0.5 M H_2_SO_4_. Absorbance readings were taken at 450 nm, and the binding parameters K_D_ and B_max_ were determined via nonlinear regression curve fitting using GraphPad Prism Version 9.5.1 [39] and compared with other binding events.

Following the determination of the binding parameters K_D_ and B_max_ of the B-trisaccharide, the antigen Rh(D) was introduced and allowed to saturate the Rh(D) binding sites (third binding event). Unbound antigen Rh(D) were collected and coated on ELISA plates. An indirect ELISA was performed as described above (using triabody), and the binding parameters K_D_ and B_max_ were determined and compared with those of the other binding events. Similarly, the intramolecular binding cooperativity of triabody was studied in other orders A→Rh(D)→B and B→A→Rh(D), B→Rh(D)→A, Rh(D)→A→B and Rh(D)→B→A.

### Determination of triabody binding behavior toward RBC-bound blood group antigens

To determine the binding behavior of a triabody designed to block blood group antigens on RBCs, an ELISA-based experiment was performed in triplicate. In this experiment the CDR-grafted humanized nanobodies (VHH) of anti-A, anti-B and anti-Rh(D) were designed and used as blocking fragments of antigens A, B and Rh(D) on RBCs.

To make the CDR-grafted humanized nanobodies, the CDR sequences of the anti-A, anti-B and anti-Rh(D) scFvs were taken and grafted onto the framework sequence of the camelid nanobody (Supplementary Figure S4). After that, the whole sequence was humanized via llamanade web server [40]. Nanobodies were then produced from *E.coli* BL21(DE3) and the effect of changes in pH on the interaction of complexes of both triabody with antigens and nanobodies with antigens was determined. The criteria for the selection of nanobodies as blocking fragments were as follows: (1) the first blocking nanobody must be sensitive to slight fluctuations in pH compared with the second blocking nanobody, and (2) pH-dependent dissociation must occur at a different pH from that of the triabody. The pH values required for the complete dissociation of several blood group nanobodies are given in Supplementary Table S2. The selected blocking nanobodies (Supplementary Table S3) were then resuspended in TBS buffer (pH 7.4) in a concentration of 10 mg/ml.

To study the order, A→B→Rh(D) (an overview of the process is given in Supplementary Figure S5), AB+ RBCs (1 µl) were immobilized on ELISA plates using 0.3% glutaraldehyde as previously described by Koganei (2007) [41] and incubated in 100 µl of a solution of anti-B VHH (first blocking) for one hour under slow shaking. A similar blocking step was followed for anti-Rh(D) (second blocking) with one modification: an anti-Rh(D) scFv was used instead of a nanobody in the binding orders of A→B→Rh(D) and B→A→Rh(D). In other binding orders anti-Rh(D) VHH was used. Unbound blocking fragments were removed by washing several times with TBS buffer (pH 7.4). After blocking antigens B and Rh(D), the A binding sites of triabody were allowed to interact with antigens A on the RBC by incubating 100 µl of triabody (100 µM in TBS buffer, pH 7.4) for 30 min under slow shaking. After incubation, the wells were washed three times with TBS buffer (pH 7.4). Following washing, first blocking fragment i.e., anti-B VHH was removed by washing three times with TBS (pH 7.83) buffer. After washing, 100 µl of TBS buffer (pH 7.4) was added, and the triabody’s B binding sites were allowed to interact with antigens B on the RBC surface. After antigen B was saturated, 100 µl of his-tagged anti-B ds-scFv (the concentrations and dilutions of all three ds-scFvs were the same as those of the triabody, as provided in Supplementary Table S1), which had the same sequence as the triabody’s anti-B ds-scFv, was added, and allowed to interact with unbound antigen B or to displace weakly bound B binding sites for 30 min under constant shaking. Unbound anti-B ds-scFv was taken and indirect ELISA was performed using primary and secondary antibodies as mentioned above in Section “Determination of intramolecular binding cooperativity of the triabody via free blood group antigens”. The binding parameters K_D_ and B_max_ were determined via nonlinear regression curve fitting using GraphPad Prism Version 9.5.1 and compared with the binding parameters of same antigen B in other orders.

The same procedure was followed to dissociate the second blocking fragment anti-Rh(D) scFv. The binding parameters K_D_ and B_max_ were determined by measuring the unbound his-tagged anti-Rh(D) ds-scFv via indirect ELISA and compared with the binding parameters of the same antigen in other orders. A similar process was used to measure the binding parameters for other binding orders, such as A→Rh(D)→B, B→A→Rh(D), B→Rh(D)→A, Rh(D)→A→B, and Rh(D)→B→A.

Due to natural variability in antigen density on RBCs and the number of cells per well, absolute K_D_ and B_max_ values may vary between replicates. Therefore, statistical analysis was not applied, and data were evaluated based on the relative trend in apparent binding parameters across samples.

### Coating of RBCs with a triabody and blood incompatibility related hemagglutination assays

The trispecific triabody was reconstituted in TBS buffer (pH 7.4) and then divided into 100 µl aliquots at a concentration of 100 µM. Approximately 10 µl of RBCs from ABO and Rh(D) blood types (A+, B+, O+, AB+, A−, B−, O − and AB−) were incubated in 100 µl aliquots of triabody at 37 °C for 30 min. Following incubation, the RBCs were washed with TBS buffer (pH 7.4), and this process was repeated with fresh aliquots of triabody. The remaining triabody in the supernatant was quantified via indirect ELISA by using free antigens as described in Section “Determination of intramolecular binding cooperativity of the triabody via free blood group antigens”. The incubation of RBCs with the triabody was repeated until no further changes in the triabody concentration in the supernatant was observed.

Immediate IgM-mediated and incompatible plasma-based hemagglutination assays were performed to evaluate both the effectiveness of triabody coating and its ability to block all blood group antigens. In the IgM-mediated assay, 1 µl of triabody-coated RBCs was incubated with 100 µl of anti-A IgM, anti-B IgM, or anti-Rh(D) IgM antibodies for 10 min at room temperature.

In the incompatible plasma-based hemagglutination assay, 20 µl of triabody-coated AB+ RBCs were incubated with 500 µl of O− plasma containing naturally occurring anti-A, anti-B, and anti-Rh(D) antibodies (only plasma positive for anti-Rh(D) antibodies was used) for 5 h at 37 °C under constant slow shaking. Hemagglutination was assessed both visually and microscopically using ICC.

## Results

### Structures of triabody

**Fig. 2:**
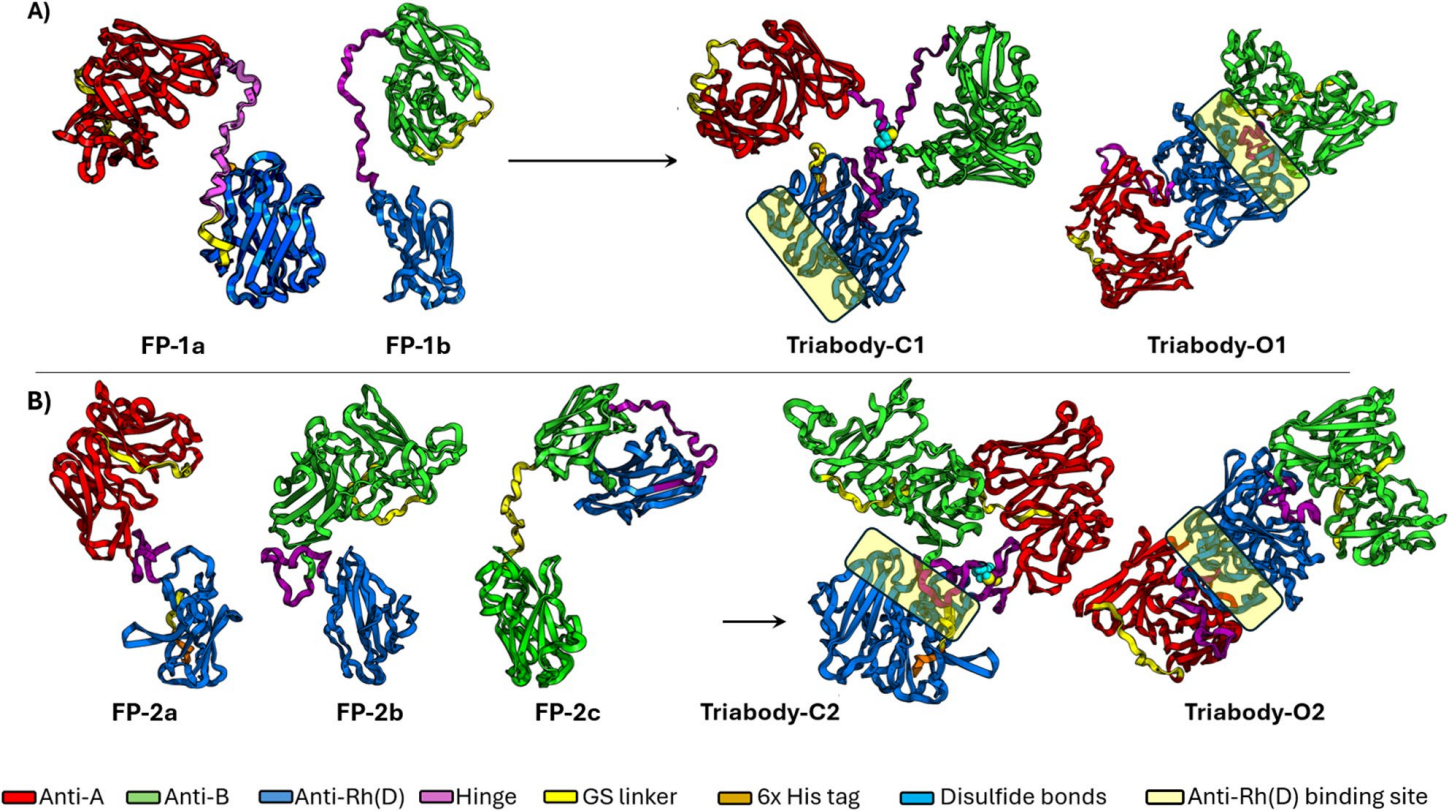
Triabodies generated from the pairing of two fusion proteins from each combination. (**A**) Pairing of fusion proteins of first combination produced both functionally closed (C1) and open (O1) forms of triabody. (**B**) In second combination, pairing of fusion proteins FP-2a and FP-2b produced one non-functionally closed (C2) and one functionally open (O2) form of triabody

On the basis of the placement of variable chains of anti-Rh(D), two combinations were used to model the molecules of the triabody (Fig. 1). As shown in Fig. 2, the fusion proteins FP-1a and FP-1b generated from combination one showed correct pairing of VH chains of both anti-A and anti-B ds-scFv with their respective VL chains. On the other hand, three types of fusion proteins (FP-2a, FP-2b and FP-2c) were observed from combination two. Like combination one, the fusion proteins FP-2a and FP-2b in combination two exhibited correct pairing of variable chains. However, the fusion protein FP-2c exhibited the interaction of VL chain of anti-Rh(D) with the VH chain of anti-B ds-scFv.

For triabodies to effectively block blood group antigens, all three of their antigen binding sites must be freely accessible. Two types of triabody structures were produced from both combinations. Depending on the location of the disulfide bond formed these structures were in closed and open forms with intramolecular and intermolecular disulfide bonds respectively. The docking of two fusion proteins from combination one produced a closed triabody-C1 form with the antigen binding sites of all three Fv facing outward and an open triabody-O1 form where all the binding sites were easily accessible. Docking of two fusion proteins from combination two with correct pairing (FP-2a and FP-2b) produced an open triabody-O2 form similar to triabody-O1 of combination one and a closed triabody-C2 form with antigen binding sites of anti-A and anti-B ds-scFv facing outward and antigen binding site of anti-Rh(D) facing inward (blocked by intermolecular disulfide bond).

MD simulations were performed to assess the triabodies’ structural dynamics. The structural behavior of triabody-C1 and O1 was evaluated using radius of gyration (R_g_), antigen binding site distances, and MD trajectories. In triabody-C1, the R_g_ (Supplementary Figure S6A) decreased from ~ 3.5 nm to ~ 3.2 nm by ~ 100 ns, indicating compaction. The A–B distance fluctuated between ~ 7.0 and ~ 9.5 nm, while the A–Rh(D) and B–Rh(D) distances stabilized after ~ 125 and ~ 80 ns, respectively, suggesting closer scFv association (Supplementary Figures S6B-D). Snapshots show rotational reorientation of the scFvs throughout the simulation, with antigen binding sites of anti-A and anti-B ds-scFvs adopting new orientations, while the anti-Rh(D) dsFv exhibits limited movement, maintaining its orientation (Supplementary Figures S7A and S7B). These observations suggest that triabody-C1 adopts a compact, stable conformation while retaining limited flexibility between its binding domains.

In triabody-O1, the R_g_ increased from ~ 3.0 nm to ~ 3.7 nm (Supplementary Figure S6E), and the A–B distance from ~ 13.2 nm to ~ 14.5 nm, reflecting elongation (Supplementary Figure S6F). Snapshots show pronounced rotational reorientation of the anti-A and anti-B ds-scFvs at the end of the simulation, while the anti-Rh(D) dsFv remains comparatively constrained, indicating differential mobility and a more extended, flexible structure. This indicates that triabody-O1 adopts an elongated conformation with increased mobility observed primarily in the anti-A and anti-B ds-scFvs at the simulation’s end (Supplementary Figures S7D and S7E).

**Fig. 3:**
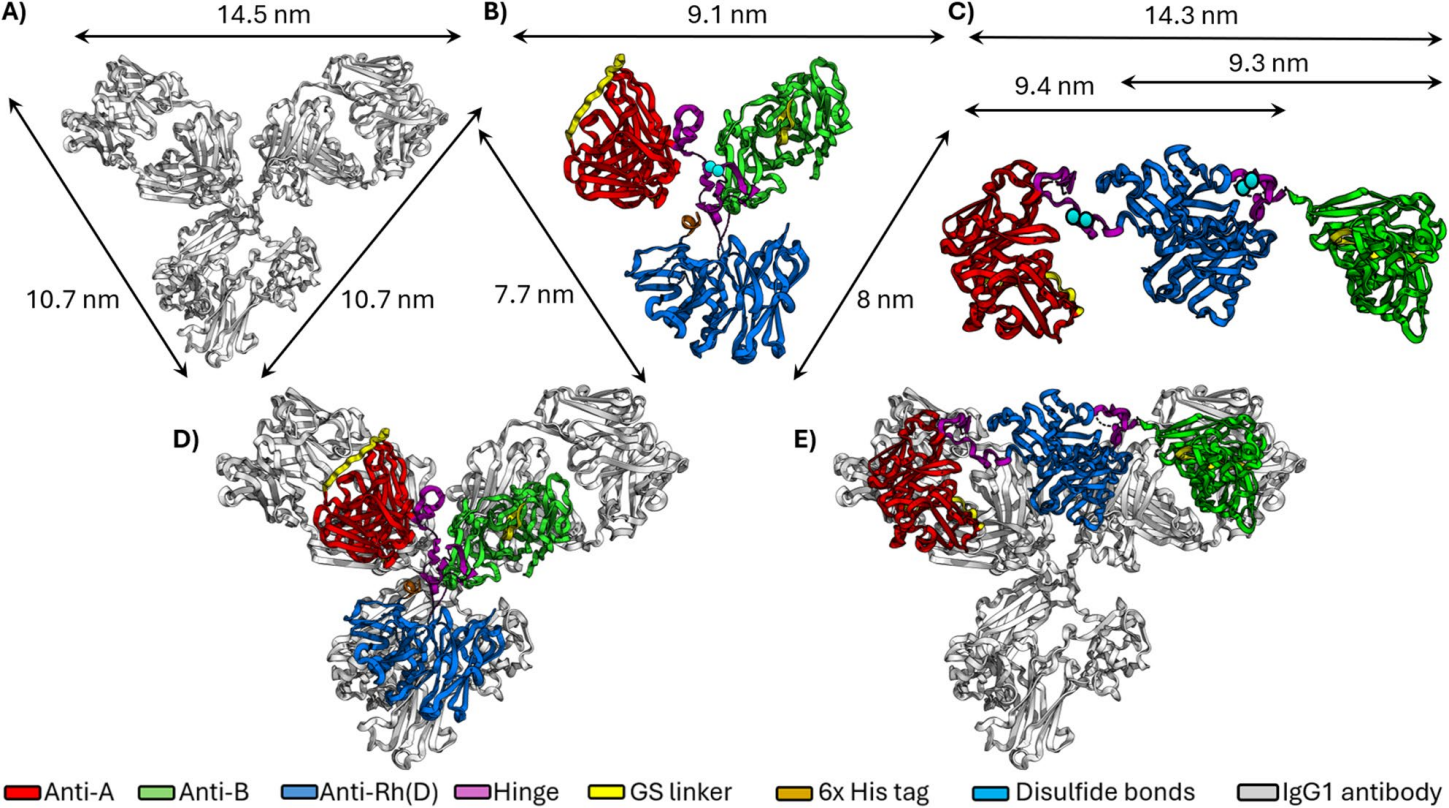
Comparison of molecular dimensions of antibody and triabodies. (**A**) Dimensions of IgG1 antibody. (**B**) Dimensions of closed form of triabody-C1. (**C**) Dimensions of open form of triabody-O1. (**D**) Superimposed models of IgG1 antibody and triabody-C1. (**E**) Superimposed models of IgG1 antibody and triabody-O1

Overall, the best structures of triabodies were obtained from the fusion proteins of combination one. The selected triabodies had ten disulfide bonds (eight intramolecular and two intermolecular) in the closed C1 form and eleven disulfide bonds (ten intramolecular and one intermolecular) in the open O1 form. The predicted theoretical weight of both triabodies was 83.3 kDa. Figure 3 shows the comparison of triabodies with the IgG1 antibody structure PDB: 1IGY [42]. Additional computational studies such as molecular docking and MMPBSA were conducted on closed structure i.e., triabody-C1.

### Functional analysis

MMPBSA calculations were performed to predict the intramolecular cooperative binding affinities of the triabody. The results shown in Table 1 revealed no significant changes in the binding free energies of the complexes formed between the triabody and each blood group antigen, regardless of the binding order. For instance, the binding free energy of the triabody–antigen A complex remained consistently around − 13.20 kcal/mol, irrespective of whether antigen A was the first, second, or third to bind. Similarly, the binding free energies of the triabody–antigen B and triabody–antigen Rh(D) complexes were approximately − 12.6 kcal/mol and − 49 kcal/mol, respectively. These results indicate a lack of intramolecular cooperativity, meaning that the binding of one antigen is independent of the binding of the others. Supplementary Figure S8 shows a snapshot from the MD trajectory of the triabody complexes with all three blood group antigens at the end of the 150 ns simulation, following the binding sequence A→B→Rh(D).

**Table 1:** Binding free energies (ΔG_Bind_) kcal/mol computed by MMPBSA

Binding sequence	First binding		Second binding		Third binding	
A→B→ Rh(D)	A	−13.20	B	−12.59	Rh(D)	−48.9
A→ Rh(D)→B	A	−13.18	Rh(D)	−49.1	B	−12.61
B→A→ Rh(D)	B	−12.60	A	−13.19	Rh(D)	−48.8
B→ Rh(D)→A	B	−12.62	Rh(D)	−49.1	A	−13.21
Rh(D)→A→B	Rh(D)	−48.4	A	−13.20	B	−12.59
Rh(D)→B→A	Rh(D)	−49.0	B	−12.60	A	−13.22

ΔG values were computed using the MMPBSA method and represent theoretical estimates of binding free energy

### Three-step purification

**Fig. 4:**
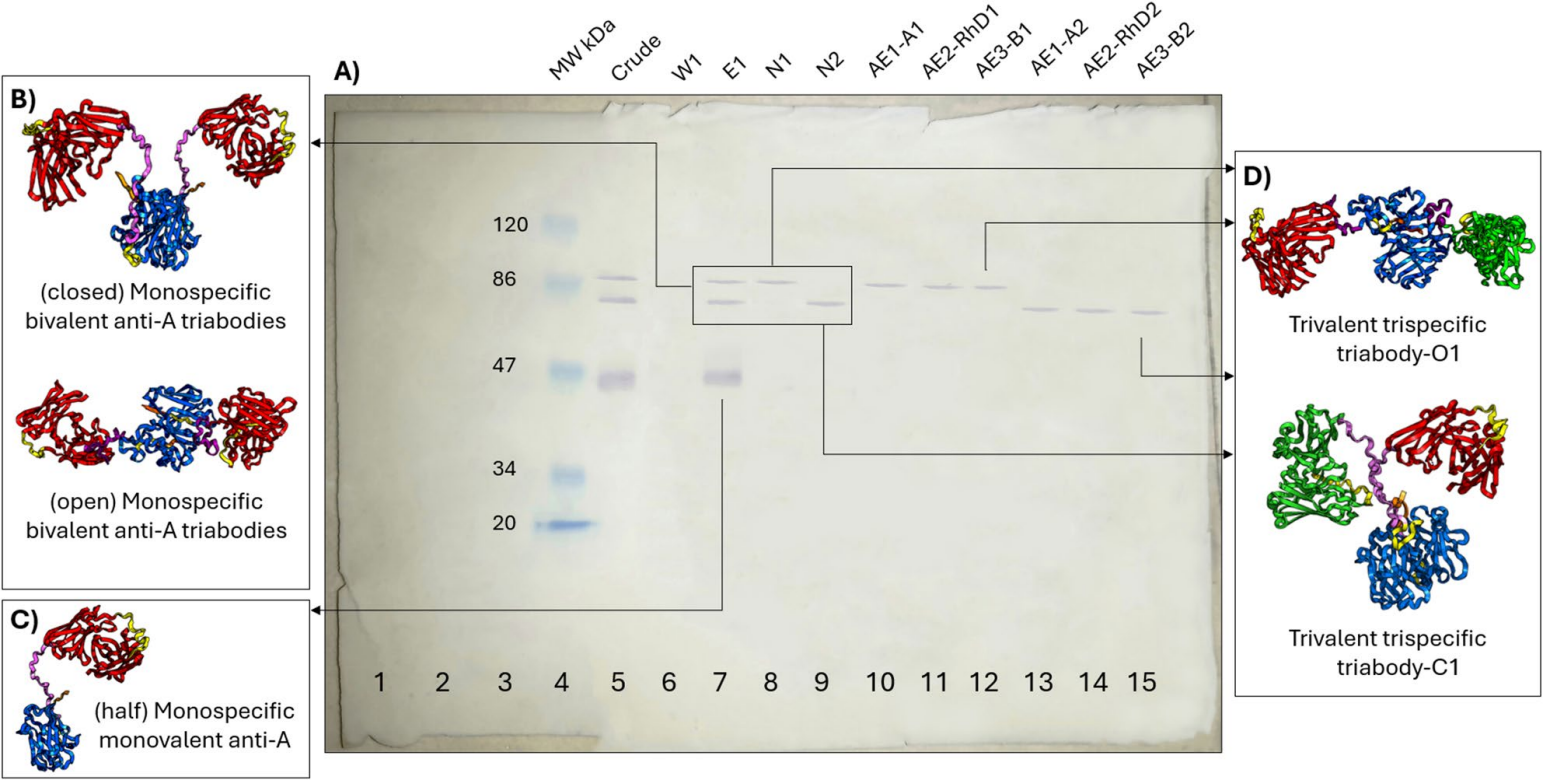
(**A**) TMB stained western blot showing purification of trifunctional triabody in a three-step process. Lanes 5–7 show purification through Ni^2+^-NTA chromatography. E1 shows purified his-tagged proteins at ~ 45 kDa, ~ 83 kDa and ~ 87 kDa. Lanes 8 and 9 show second purification through Native PAGE gel. N1 (lane 8) and N2 (lane 9) show bands at ~ 87 kDa and ~ 83 kDa respectively. Lanes 10–15 show last purification through acid-glycine elution. AE1-A1 (lane 10), AE2-RhD1 (lane 11) and AE3-B1 (lane 12) show purification of functional triabody form N1 fraction with functional A, Rh(D) and B sites, respectively. AE1-A2 (lane 13), AE2-RhD2 (lane 14) and AE3-B2 (lane 15) show purification of functional triabody from N2 fraction with functional A, Rh(D) and B sites, respectively. (**B**) Monospecific anti-A his-tagged triabodies purified in E1, N1 and N2 elution fractions. (**C**) Monospecific anti-A his-tagged half triabody purified in E1 elution fraction. (**D**) Trivalent triabodies purified in E1, N1, N2, AE3-B1 and AE3-B2 elution fractions

Triabody was produced under slow induction conditions (18 °C and 0.25 mM IPTG) to allow proper folding of the protein. His-tagged proteins were purified via Ni^2+^-NTA chromatography (first purification step) (Fig. 4A) and analyzed via western blotting. As shown in Fig. 4A, in the Ni^2+^-NTA elution profile E1 (lane 7), the molecular weight of the triabody (~ 83 kDa) was approximately the same as the theoretical weight predicted by the ProtParam tool. In addition to the predicted triabody, a larger triabody of ~ 87 kDa and a half triabody of ~ 45 kDa were also observed.

Protein extraction from Native PAGE gel was a second step in separating individual triabody species. The Ni^2+^-NTA elution profile E1 was taken, and Native PAGE was carried out. Subsequently, each individual triabody species was extracted from the gel. As shown in Fig. 4A, a larger triabody (lane 8) and desired-sized triabody (lane 9) were successfully isolated from a mixture of different-sized his-tagged proteins.

Purification through acid-glycine elution was the final crucial step in obtaining a trifunctional triabody. Following Native PAGE purification, the elution profiles for the N1 fractions were AE1-A1, AE2-RhD1, and AE3-B1, whereas the elution profiles for the N2 were AE1-A2, AE2-RhD2, and AE3-B2. As shown in Fig. 4A, the elution profiles AE1-A1 (lane 10) and AE1-A2 (lane 13) show bands at ~ 87 kDa and ~ 83 kDa respectively. These bands represent the purification of triabodies with functional A binding sites. Similarly, the bands observed in the elution profiles of AE2-RhD1 (lane 11) and AE2-RhD2 (lane 14) represent the purified bifunctional triabodies with functional A and Rh(D) binding sites. Finally, bands in elution profiles AE3-B1 (lane 12) and AE3-B2 (lane 15) show purified trifunctional triabodies (~ 87 kDa and ~ 83 kDa, respectively) with functional A, Rh(D) and B binding sites. Figure 4B and C, and 4D depict the expected purified proteins in each step.

The SEC chromatogram of the final products showed two distinct peaks corresponding to AE3-B1 and AE3-B2, indicating purity of triabody species with different conformations and demonstrating that each fraction was largely homogeneous and free from significant aggregates or contaminants (Supplementary Figure S9A). Non-reducing SDS-PAGE (Supplementary Figure S9B) showed a single band for AE3-B2 at ~ 83 kDa, while AE3-B1 displayed two bands at ~ 45 kDa. This indicates that the triabody fraction in AE3-B2 contains intact interdomain disulfide bonds, whereas AE3-B1 lacks interdomain disulfide bonds.

### Hemagglutination assay and immunocytochemistry (ICC)

**Fig. 5:**
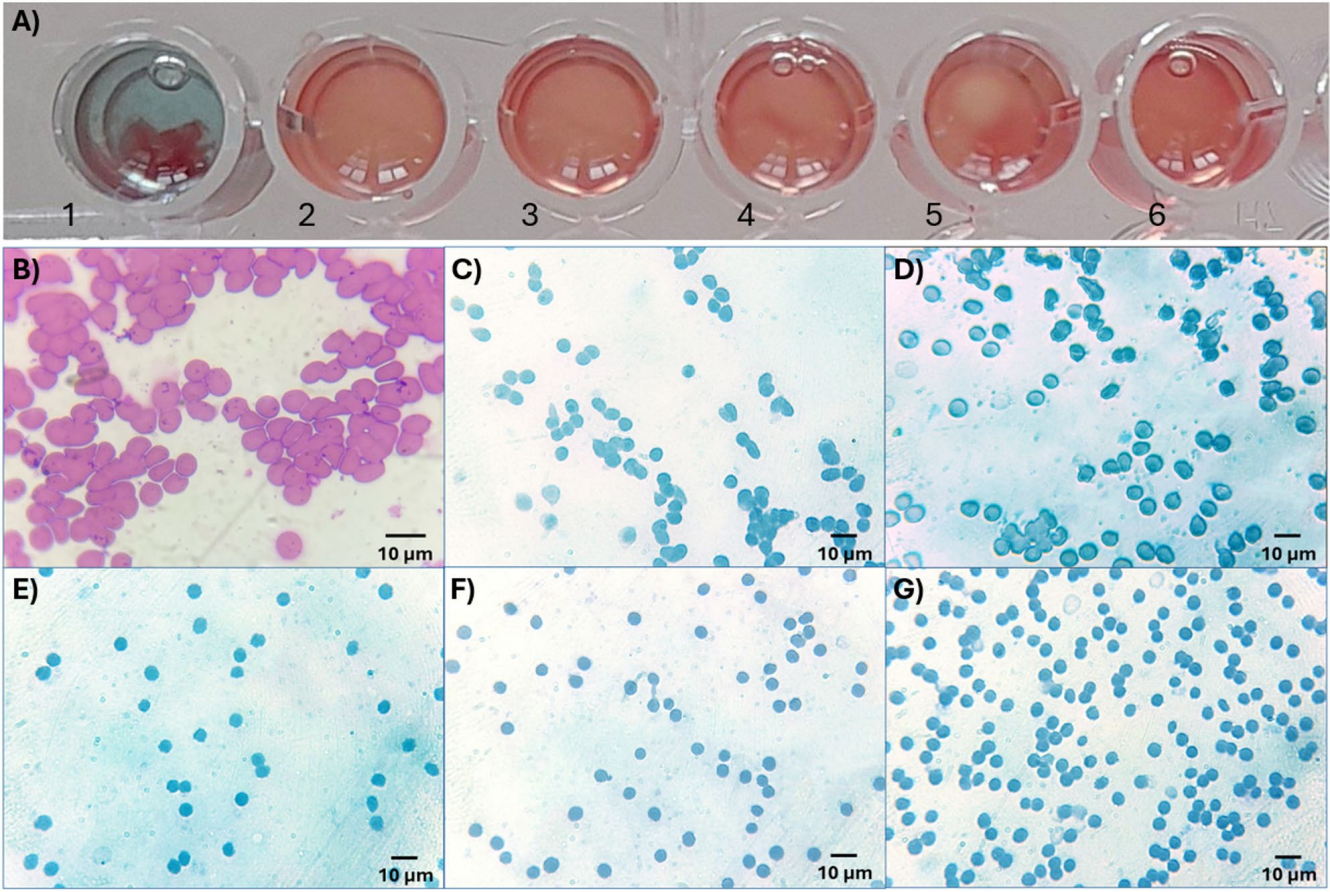
(**A**) Hemagglutination testing. Well 1 shows control (A+ RBCs in anti-A IgM antibodies). Wells 2 to 5 shows AB+, AB−, A + and B+ RBCs in triabody from AE3-B1 fraction. Well 6 shows AB+ RBCs in triabody from AE3-B2 fraction. (**B**) (control) Giemsa-stained A+ RBCs show complete hemagglutination in the presence of anti-A IgM antibodies. (**C**) Mixed-field hemagglutination of AB+ RBCs coated with triabody from AE3-B1 fraction. (**D**) Mixed-field hemagglutination of AB− RBCs coated with triabody from AE3-B1 fraction. **E**). No hemagglutination of A+ RBCs coated with triabody from AE3-B1 fraction. **F**) No hemagglutination of B+ RBCs coated with triabody from AE3-B1 fraction. **G**) No hemagglutination of AB+ RBCs coated with triabody from AE3-B2 fraction

Immunocytochemistry (ICC) was performed to observe the hemagglutinating properties of the triabodies. For this purpose, RBCs with at least two blood group antigens were taken and coated with triabodies. As shown in Fig. 5A no visible hemagglutination of RBCs was observed when these RBCs were incubated with triabodies from either the AE3-B1 (wells 2–5) or the AE3-B2 (well 6) fraction. However, when examined microscopically, mixed-field hemagglutination was observed in AB+ (Fig. 5C) and AB− (Fig. 5D) RBCs that were incubated with triabody from the AE3-B1 fraction. In contrast, no hemagglutination was observed in A+ (Fig. 5E) and B+ (Fig. 5F) RBCs incubated with the same triabody. Similarly, no hemagglutination was observed in AB+ RBCs (Fig. 5G) incubated with triabody from the AE3-B2 fraction.

A PEH assay was performed to further confirm the hemagglutinating properties of triabodies. As shown in Supplementary Figure S10A, no hemagglutination was observed in the ficin-treated RBCs or in RBCs incubated with the triabody from AE3-B2 fraction (Supplementary Figure S10B), whereas hemagglutination was observed in RBCs incubated with the AE3-B1 triabody fraction (Supplementary Figure S10C).

### Determination of triabody binding behavior toward free and RBC-bound blood group antigens

ELISA-based experiments were used to evaluate the binding behavior of the triabody toward RBC-bound antigens. Free antigens were included to determine whether observed changes in binding patterns resulted from the cell-surface environment or from the structural constraints of the triabody during initial and subsequent binding events.

**Fig. 6:**
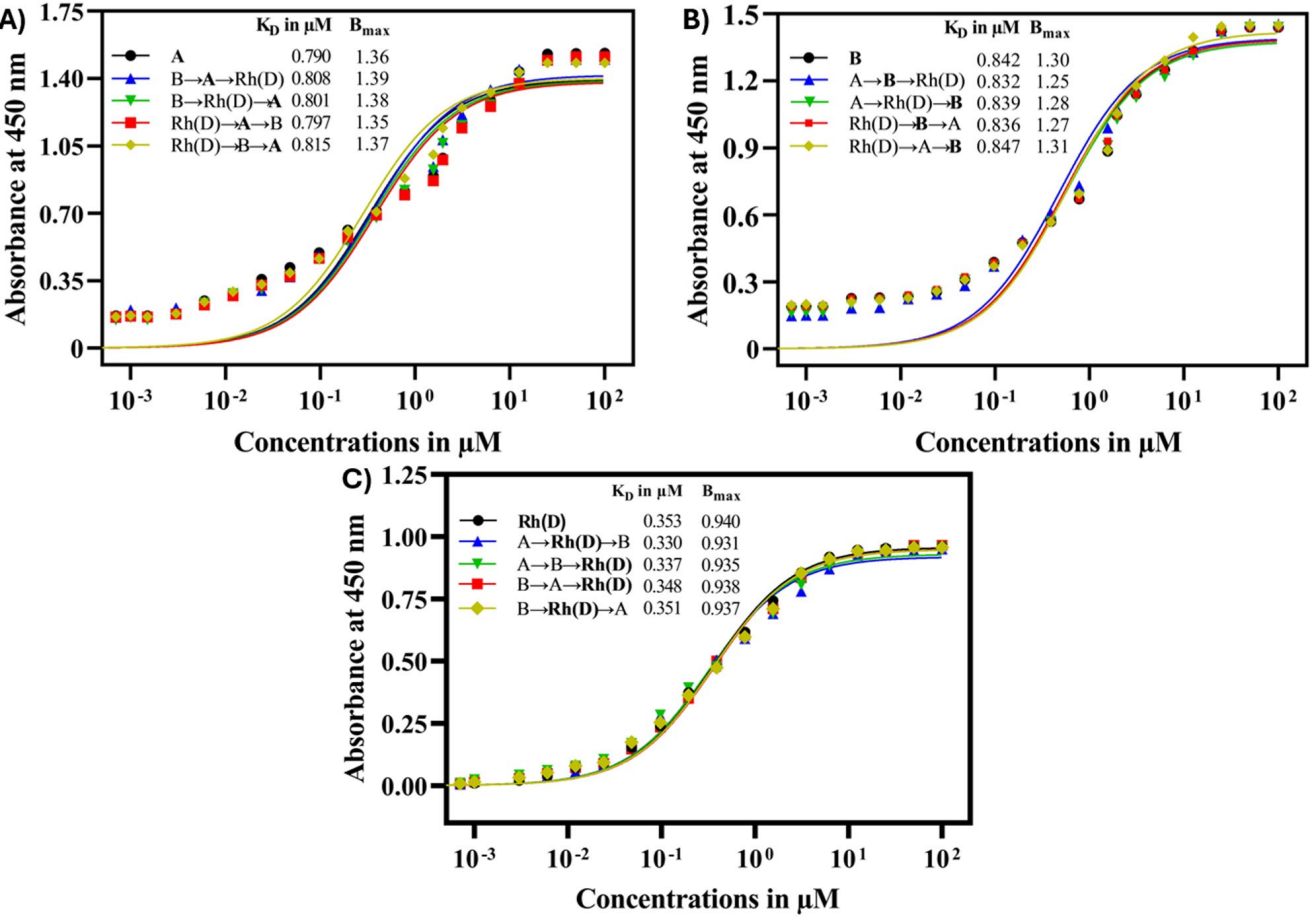
Binding cooperativity of the triabody with free blood group antigens. The binding parameters K_D_​ and B_max_ are shown for (**A**) anti-A, (**B**) anti-B and (**C**) anti-Rh(D) binding sites of triabody. The x-axis represents the triabody log concentration (µM), and the y-axis represents the absorbance at 450 nm

As shown in Fig. 6, there were no significant overall changes in the binding parameters K_D_ and B_max_, suggesting non-cooperative binding, i.e., subsequent binding was not influenced by prior binding events. The K_D_​ values for the anti-A, anti-B, and anti-Rh(D) binding sites of the triabody, when binding individually, were 0.790, 0.842, and 0.353 µM, respectively. For the binding orders where A-trisaccharide (Fig. 6A) was involved in the second binding event (B→A→Rh(D) and Rh(D)→A→B), the K_D_​ values were 0.808 and 0.797 µM respectively. Similarly, in the third binding event involving A-trisaccharide (B→Rh(D)→A and Rh(D)→B→A), the K_D_​ values were 0.801 and 0.815 µM respectively. The B_max_ values of A-trisaccharide for both the second and third binding events remained close to the B_max_ value of the initial binding event (1.36), with values of 1.39 and 1.35 for the second binding events (B→A→Rh(D) and Rh(D)→A→B), and 1.38 and 1.37 values for the third binding events (B→Rh(D)→A and Rh(D)→B→A).

In the case where B-trisaccharide (Fig. 6B) was involved in the second binding event, the K_D_​ values were 0.832 µM and 0.836 µM for the orders A→B→Rh(D) and Rh(D)→B→A, respectively. In the third binding event, the K_D_​ values were 0.839 µM and 0.847 µM for the orders A→Rh(D)→B and Rh(D)→A→B, respectively. Similar to the A-trisaccharide, the B_max_ values for the B-trisaccharide were close to the initial value of 1.30. The second binding events yielded B_max_ values of 1.25 and 1.27 for the A→B→Rh(D) and Rh(D)→B→A binding orders, respectively. The third binding events produced B_max_ values of 1.28 for the A→Rh(D)→B order and 1.31 for the Rh(D)→A→B order.

For the second binding events in the orders A→Rh(D)→B and B→Rh(D)→A, which involve the antigen Rh(D) Fig. 6C), the K_D_ values were 0.330 and 0.351 µM, respectively. A similar case was observed for the other binding orders, A→B→Rh(D) and B→A→Rh(D), which involve the antigen Rh(D) in the third binding position, with K_D_ values of 0.337 and 0.348 µM, respectively. The changes in B_max_ values for antigen Rh(D) were similar to those observed for A-trisaccharide and B-trisaccharide, i.e., the B_max_ values for the second and third binding events were closer to the initial value (0.940). The B_max_ values for the binding order A→Rh(D)→B and B→Rh(D)→A, involving Rh(D) in the second binding event, were 0.931 and 0.937, respectively. For the third binding events A→B→Rh(D) and B→A→Rh(D), the B_max_ values were 0.935 and 0.938, respectively.

The binding behavior of the triabody with RBC-bound antigens was indirectly assessed by measuring changes in the apparent binding parameters K_D_ and B_max_ of the anti-A, anti-B, and anti-Rh(D) ds-scFvs. This approach was crucial for understanding the observed cooperativity and how it is influenced by the physical environment of the antigen, the small size of the triabody and the conformational flexibility of its monomeric scFv units.

**Fig. 7:**
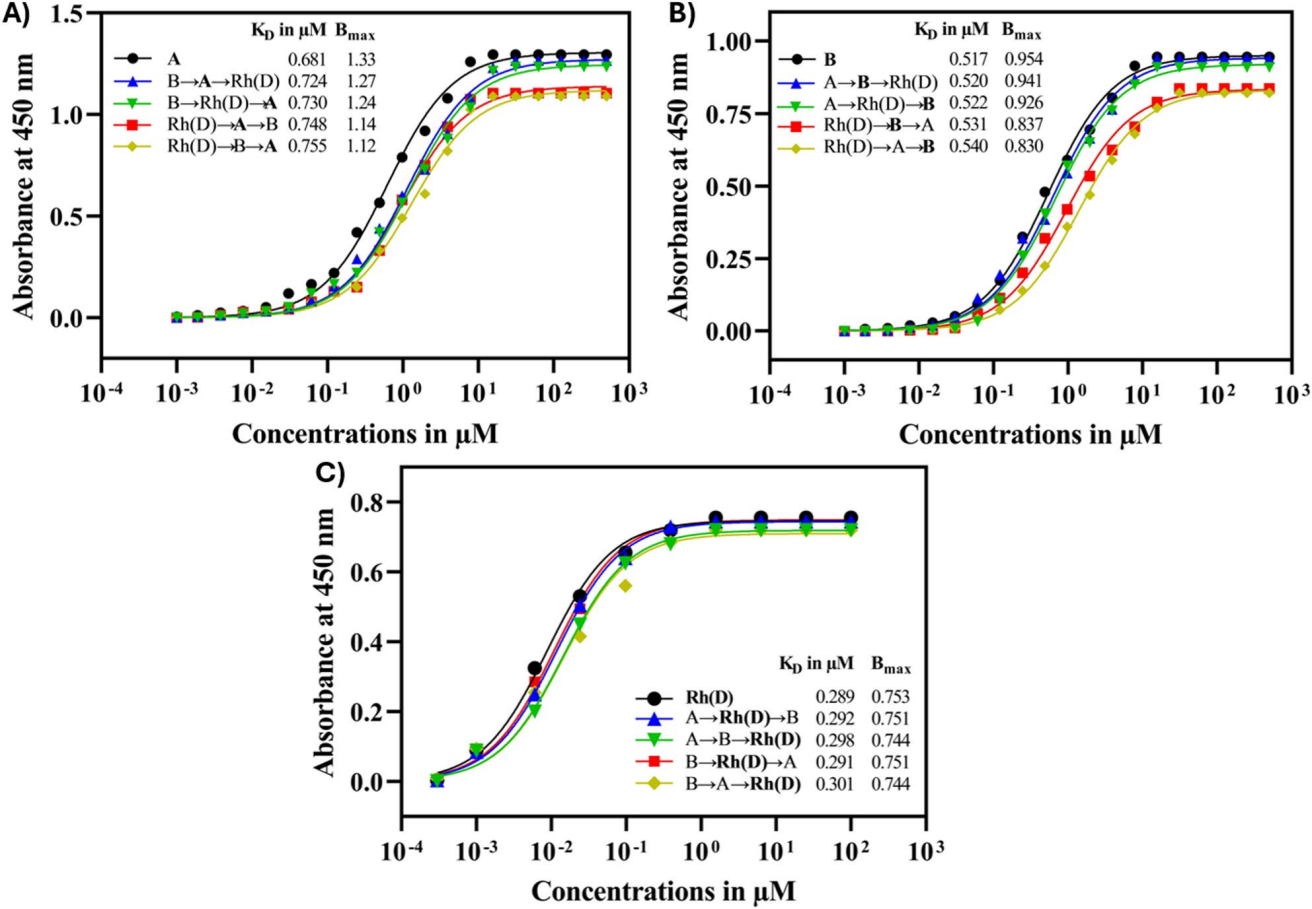
Binding behavior of the triabody toward RBC-bound blood group antigens. The binding parameters K_D_ and B_max_ are shown for (**A**) anti-A ds-scFv, (**B**) anti-B ds-scFv and (**C**) anti-Rh(D) ds-scFv. The x-axis represents the ds-scFv log concentration (µM), and the y-axis represents the absorbance at 450 nm

As shown in Fig. 7A, antigen A exhibited higher K_D_ values for both the second and third binding events compared with its initial binding (0.618 µM). When antigen B was bound first, the K_D_ values for antigen A increased to 0.724 µM and 0.730 µM in the sequences B→A→Rh(D) and B→Rh(D)→A, respectively. A further increase was noted when antigen Rh(D) served as the first binding partner, with K_D_ values reaching 0.748 µM and 0.755 µM for Rh(D)→A→B and Rh(D)→B→A, respectively. After initial binding to antigen B, the B_max_ value for antigen A decreased from 1.33 to 1.27 and 1.24, whereas even lower values of 1.14 and 1.12 were observed when antigen Rh(D) was bound first.

In contrast to antigen A, the K_D_ values for the second and third binding events involving antigen B were found to remain comparable to those observed when antigen B was the first antigen to bind (0.517 µM) as shown in Fig. 7B. When antigen A was bound first, the K_D_ values for antigen B were 0.520 µM and 0.522 µM for the sequences A→B→Rh(D) and A→Rh(D)→B, respectively. In comparison, when antigen Rh(D) was involved in the initial binding event, higher K_D_ values were observed, i.e., 0.533 µM and 0.540 µM for the sequences Rh(D)→B→A and Rh(D)→A→B, respectively. Following the initial binding of antigen A, the B_max_ values for antigen B were observed to remain close to its initial value (0.954), at 0.941 and 0.926 for the sequences A→B→Rh(D) and A→Rh(D)→B, respectively. However, B_max_ values were significantly reduced to 0.837 and 0.830 when antigen Rh(D) was the first antigen to bind, as observed in the sequences Rh(D)→B→A and Rh(D)→A→B (Fig. 7B).

Unlike antigens A and B, the binding of antigen Rh(D) remained largely unaffected by prior binding events. As shown in Fig. 7C when antigen Rh(D) was involved in the second binding event, the K_D_ values were 0.292 µM and 0.291 µM for the sequences A→Rh(D)→B and B→Rh(D)→A, respectively, which were comparable to its initial binding value of 0.289 µM. The B_max_ value was 0.751, which was consistently maintained in second binding events close to the initial binding value of 0.753. However, when Rh(D) was the third antigen to bind, the K_D_ values increased slightly to 0.298 µM and 0.301 µM for the sequences A→B→Rh(D) and B→A→Rh(D), respectively, with corresponding B_max_ values of 0.744.

A similar trend in binding behavior was observed in other replicates, as shown in Supplementary Figures S11 and S12.

### Coating of RBCs with a triabody and blood incompatibility related hemagglutination assays

To enable RBCs from different blood groups to display enhanced compatibility, the triabody must effectively block the major blood group antigens A, B, and Rh(D). To achieve this, a repeated exposure protocol was used, which involved incubating RBCs in concentrated aliquots of a triabody solution. Supplementary Figure S13 shows that for all blood types of RBCs, a significant decrease in the concentration of triabody was observed in the first aliquot after 30 min of incubation. However, no further reduction in concentration was observed in aliquots 2 and 3. In the case of O– RBCs, no reduction was observed in any aliquot due to the absence of blood group antigens.

**Fig. 8:**
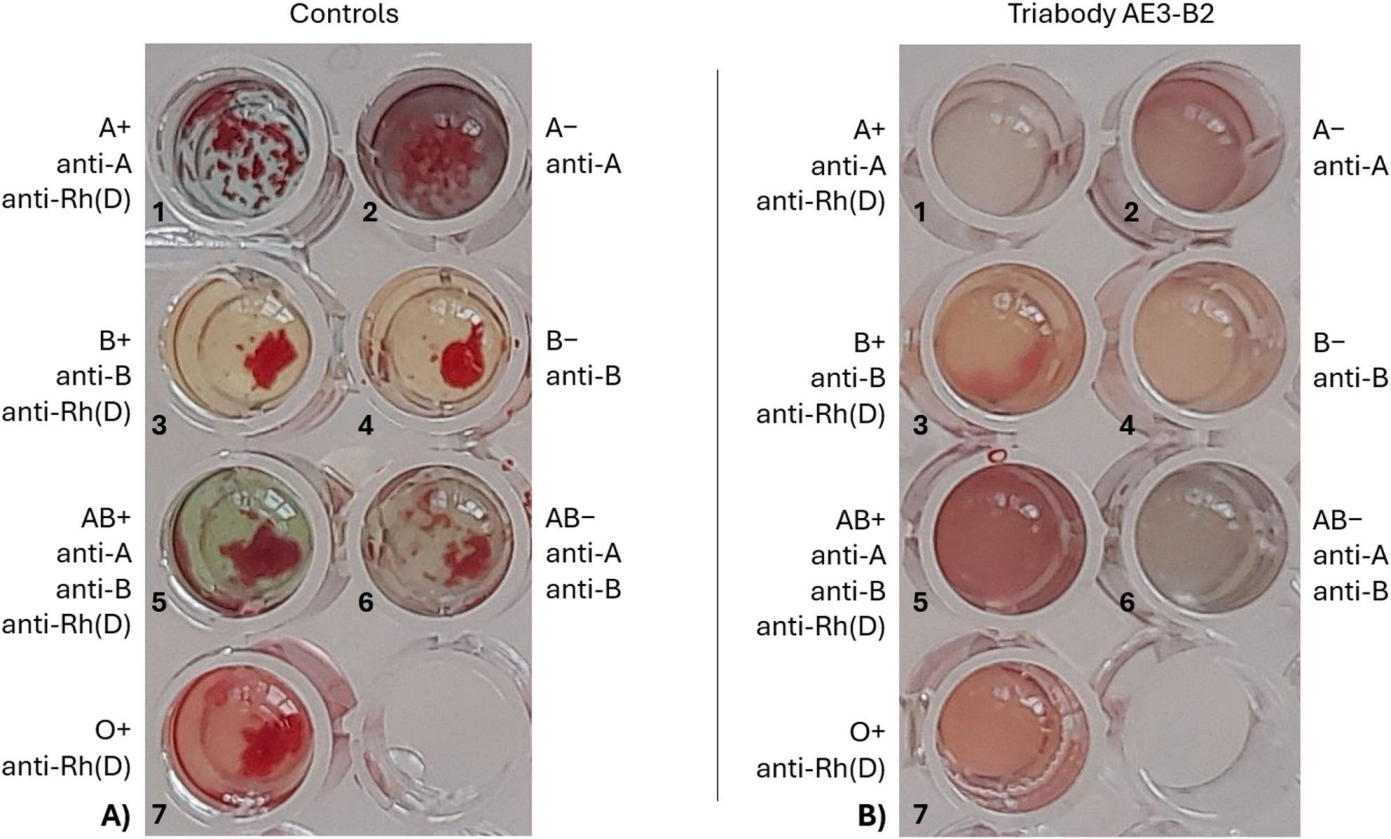
IgM-mediated hemagglutination assay. (**A**) Control wells consisting of hemagglutinated RBCs in corresponding blood group IgM antibodies. (**B**) RBCs coated with triabody from the AE3-B2 fraction in blood group IgM antibodies

The overall results (Fig. 8) of the IgM-mediated hemagglutination assay were negative, indicating that RBCs coated with trispecific triabodies purified in fraction AE3-B2 (Fig. 8B) did not hemagglutinate in the presence of associated anti-A IgM, anti-B IgM and anti-Rh(D) IgM antibodies. The control wells in Fig. 8A show positive hemagglutination of uncoated RBCs of different blood types in the presence of associated blood group antibodies. Supplementary Figure S14 shows TMB-stained, non-hemagglutinated triabody-coated AB+ RBCs.

**Fig. 9:**
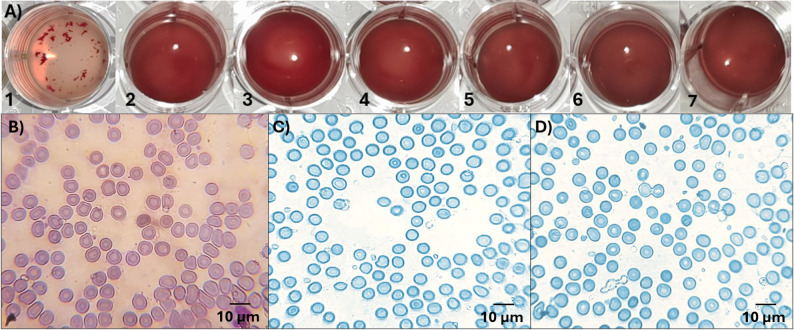
(**A**) (control) Well 1 shows hemagglutination of uncoated AB+ RBCs in O– blood plasma. (control) Well 2 shows whole O– blood. Wells 3–7 show AE3-B2 triabody-coated AB+ RBCs in incompatible O– blood plasma after 1 to 5 h, respectively. (**B**) (control) Giemsa-stained non-hemagglutinated O– RBCs in whole blood. (**C**) TMB-stained non-hemagglutinated AE3-B2 triabody-coated RBCs after 1 h. (**D**) TMB-stained non-hemagglutinated AE3-B2 triabody-coated RBCs after 5 h

The incompatible plasma-based hemagglutination assay was performed to assess the effectiveness of triabody coating in blocking surface blood group antigens on AB+ RBCs and preventing antibody-mediated hemagglutination. The assay was conducted under in vivo-like conditions at 37 °C using incompatible O– plasma. Under these conditions, triabody-coated AB+ RBCs showed no visible hemagglutination even after 5 h of incubation. Visual evaluation revealed a uniform RBC suspension without macroscopic clumping (Fig. 9A), and microscopic analysis further confirmed the absence of hemagglutinate formation, with RBCs remaining evenly dispersed and morphologically intact (Fig. 9C and D). These results demonstrate that triabody coating effectively blocks blood group antigens on AB+ RBCs, allowing them to display hemagglutinating patterns similar to those of O– RBCs, as illustrated by well 2 in Fig. 9A and the micrograph in Fig. 9B, and preventing plasma antibody–induced hemagglutination under physiologically relevant conditions.

## Discussion

The main aim of this research was to design a novel trispecific triabody capable of acting as a blocking fragment for blood group antigens A, B, and Rh(D). By blocking these key antigens, RBCs of any blood type can reduce the risk of antibody-mediated hemagglutination and potentially enable safer transfusions regardless of the recipient’s native blood type. The design of the triabody was inspired by the compact, Y-shaped structure of IgG antibodies. Unlike IgM antibodies, IgG molecules are well known to rarely induce direct hemagglutination of RBCs due to their smaller size [43]. To further minimize the potential for hemagglutination, the triabody was intentionally designed to be even smaller than an IgG antibody. Furthermore, in addition to its trivalent binding capability, triabody was designed to be trispecific. These combined features would allow the triabody to efficiently block multiple blood group antigens without causing the hemagglutination of RBCs.

To design a compact Y-shaped triabody, the hinge region of an IgG1 antibody was used. The hinge region, similar to a glycine-serine (GS) linker, provides flexibility to protein structures. However, unlike GS linker, the flexibility and movement of the hinge region can be controlled by the presence of cysteine residues. These cysteine residues are capable of forming intermolecular disulfide bonds between two hinge regions, thereby stabilizing the overall structure [44–46]. As shown in Fig. 2, the addition of hinge regions in the triabodies offered a distinct advantage over GS linkers by producing compact Y-shaped structures (triabody-C1 and C2) that closely mimic the native IgG structure. Such structural compactness would not have been achieved if a GS linker had been used instead.

In general, scFvs possess a well-conserved framework and exhibit a remarkable functional diversity. Certain residues within the framework of a scFv are highly conserved and act as essential building blocks for the basic architecture of the molecule. These residues, through various interactions, provide shape and stability to the scFv e.g., cysteines are involved in intradomain disulfide bonds. Functional diversity arises from the hypervariable regions, which provide binding specificity to scFvs [47]. As shown in Fig. 2B, incorrect pairing was observed in the fusion protein FP-2c. This mispairing could be due to two reasons: (1) similar frameworks of both chains and (2) the close proximity of the VH chain of anti-B to the VL chain of anti-Rh(D). The VH and VL frameworks of the Fvs used in this study share a high degree of similarity, with the only significant difference located in the sequence of complementarity-determining regions (CDRs) responsible for binding specificity. This similarity, combined with the close proximity of the chains, may have facilitated the incorrect pairing of the VL chain of anti-Rh(D) with the VH of anti-B within FP-2c.

The accessibility of antigen binding sites is essential for triabodies to function effectively. This was clearly observed in triabodies-C1 and C2 (Fig. 2). In triabody-C1, the arrangement of variable chains positioned the anti-Rh(D) binding site outward, making it accessible. Conversely, the same variable chains in triabody-C2 positioned the anti-Rh(D) binding site inward, rendering it inaccessible. This issue in triabody-C2 could have been prevented by incorporating an additional GS linker of at least 10 amino acids (G_4_S)_2_ between the hinge region and the anti-Rh(D) variable chain, as shown in Supplementary Figure S15. The additional linker would have allowed reorientation and facilitated the formation of an intermolecular disulfide bond in the opposite direction, thereby exposing the anti-Rh(D) binding site and orienting it outward. These findings suggest that the arrangement of variable chains during triabody design can impact the final pairing of chains and ultimately their functionality.

The tendency of triabodies to induce hemagglutination is a critical factor in determining their suitability as blocking agents for blood group antigens on RBCs. Although triabodies possess multiple antigen binding sites capable of mediating RBC cross-linking, their hemagglutinating activity is strongly dependent on structural organization. Size-exclusion chromatography (SEC) and non-reducing SDS-PAGE analyses demonstrated that the purified triabody fractions AE3-B1 and AE3-B2 were homogeneous but structurally distinct. AE3-B2 migrated as a single ~ 83 kDa species (Supplementary Figure S9B), consistent with a conformation stabilized by interdomain disulfide bonds, whereas AE3-B1 lacked such bonds, indicating an alternative structural arrangement. These two fractions correspond to different structural classes illustrated in Supplementary Figure S16, which summarizes the possible triabody configurations.

These structural differences were reflected in the hemagglutinating behavior of the two triabody fractions. While no macroscopic hemagglutination was observed, microscopic examination revealed mixed-field hemagglutination in AB + and AB– RBCs incubated with AE3-B1. This effect was confirmed in the PEH assay, which produced complete hemagglutination exclusively with AE3-B1. In contrast, AE3-B2 failed to induce hemagglutination in both assays, consistent with a closed structure organization. MD simulations of an open triabody (triabody-O1) provide a structural explanation: the extended A–B distance (~ 14 nm) and outward-facing orientation of the anti-A and anti-B ds-scFv binding sites (Figure S7) position the binding sites to readily bridge adjacent RBCs, consistent with the hemagglutination observed for AE3-B1.

Collectively, these findings demonstrate that hemagglutination requires an open triabody configuration, as represented by Supplementary Figures S16C and S16D, which allows sufficient inter-RBC bridging. Conversely, closed configurations, as shown in Supplementary Figures S16A and S16B, abolish hemagglutinating activity. While open triabodies like AE3-B1 can effectively block blood group antigens, their extended configuration permits residual RBC cross-linking, which could pose compatibility risks if used for transfusion.

Given that the triabody was designed to block blood group antigens on the surface of RBCs, it was essential to evaluate whether it could bind all three antigens simultaneously or whether its structural constraints or the cell-surface environment might limit this capability. To address this, binding behavior was first examined using free antigens to determine whether intramolecular cooperativity exists and then compared with RBC-bound antigens to assess whether surface-associated constraints alter apparent binding parameters. As shown in Fig. 6, no significant changes in K_D_ or B_max_ were observed during sequential binding to free antigens, indicating non-cooperative binding and suggesting that the anti-A, anti-B, and anti-Rh(D) monomers act as separate units.

In contrast, binding to RBC-bound antigens (Fig. 7) resulted in binding order-dependent changes in apparent binding parameters. Increased K_D_ values and reduced B_max_ values were observed during both the second and third binding events, with the magnitude of these effects depending on the binding order and antigen involved. These changes were most evident for antigens A and B, whereas binding to antigen Rh(D) was comparatively stable across binding sequences. These binding order-dependent changes are consistent with a combined effect of reduced availability of antigen binding sites and decreased antigen accessibility resulting from structural constraints of the triabody, rather than steric hindrance imposed solely by the surface density of antigens.

The changes were especially significant when Rh(D) was the first antigen to bind. This observation may be explained by the relatively low abundance of antigens Rh(D) on RBCs compared with carbohydrate antigens A and B [48, 49]. Fewer Rh(D) molecules require fewer triabodies to reach saturation during the initial binding. As a result, fewer triabodies remain available for subsequent interactions with antigen A or B, leading to reduced availability of antigen binding sites, as indicated by the increased K_D_ and decreased B_max_ values. In contrast, these effects were less prominent when antigen A or B was involved in the first binding event, or when Rh(D) was engaged during the second binding step.

The results of the IgM-mediated hemagglutination assays, as shown in Fig. 8, directly confirm the effectiveness of the triabody as a blocking fragment. The complete absence of hemagglutination in triabody-coated RBCs demonstrates that the triabody successfully prevents IgM antibodies from interacting with their corresponding blood group antigens. These findings are consistent with those reported by Hafeez and Zaidi (2024) [13], who similarly observed a lack of hemagglutination when antigen A blocked RBCs were incubated with anti-A IgM antibodies.

While IgM-mediated hemagglutination assays provide a stringent in vitro assessment of antigen accessibility, the first environment that triabody-coated RBCs would encounter following transfusion is incompatible recipient plasma. To evaluate triabody performance under physiologically relevant, in vivo-like conditions, an incompatible plasma-based hemagglutination assay was performed. As shown in Fig. 9, no hemagglutination was observed throughout the 5-hour incubation period at 37 °C, indicating that triabody coating effectively prevents antibody-mediated RBC aggregation under prolonged exposure to incompatible plasma. Importantly, the triabody-coated RBCs exhibited hemagglutinating patterns comparable to O– RBCs, as shown by the absence of hemagglutination in whole O– blood used as a reference control (Fig. 9A and B). This suggests that triabody-coated RBCs have the potential to reduce blood incompatibility related hemagglutination, and with further in vivo investigations, could be transfused into recipients of different blood types, thereby expanding the pool of compatible blood.

This study has several limitations that should be acknowledged. The exact number and location of inter- and intramolecular disulfide bonds in the triabody were not directly determined, limiting detailed structural insights; advanced structural analyses such as X-ray crystallography or nuclear magnetic resonance (NMR) spectroscopy are needed to map these bonds. While ELISA-based experiments provided valuable insights into binding behavior and cooperativity, relying solely on these measurements has inherent limitations, and complementary biophysical approaches such as surface plasmon resonance (SPR) or biolayer interferometry (BLI) would provide more accurate kinetic and affinity data. The distinction between closed and open triabody conformations requires direct validation using techniques such as size-exclusion chromatography coupled with multi-angle light scattering (SEC-MALS), small-angle X-ray scattering (SAXS), cryogenic electron microscopy (cryo-EM), or non-reducing mass spectrometry.

Triabody effectiveness was assessed in vitro outside a physiological environment, and its performance under complex in vivo conditions remains untested; subsequent studies using animal models immunized with human RBCs to produce human ABO and Rh(D) antibodies, followed by transfusion of triabody-coated RBCs, could evaluate efficacy, clearance, and potential immune responses. In incompatible plasma-based hemagglutination assay, the influence of other RBC-bound antigens was not considered, nor was the formation of antibody-antigen complexes determined, which should be addressed in future research. Finally, the immunogenicity of the triabody itself was not studied, and further investigations are essential to assess safety, the persistence of triabody on RBC surfaces, and interactions with standard blood bank serology and compatibility testing; for example, bound triabodies may alter the accessibility of other antigens, and steric hindrance could reduce antibody binding, potentially affecting antigen screening.

## Conclusion

This study demonstrates that the trispecific triabody can effectively block ABO and Rh(D) antigens on RBCs, preventing hemagglutination in vitro and showing hemagglutination patterns similar to O– cells. Its compact Y-shaped structure allows efficient multivalent binding while minimizing RBC cross-linking, highlighting its potential as a versatile blocking agent for transfusion medicine. With further in vivo investigation, triabody-coated RBCs could be transfused into recipients of different blood types, expanding the pool of compatible blood. Future studies should evaluate triabody-coated RBCs in animal models to assess efficacy, safety, immunological response, clearance, and interactions with standard blood bank procedures. Collectively, these investigations will provide essential data to advance the triabody toward translational and clinical applications.

## Supplementary Information

Below is the link to the electronic supplementary material.

Supplementary Material 1

Supplementary Material 2

Supplementary Material 3

## Data Availability

All data generated or analyzed during this study are included in this published article and its supplementary information files.
