# Supplementary material for: Enhancing red blood cell compatibility: in vitro hemagglutination prevention using a trispecific triabody as a blocking fragment for blood group antigens
Source: J Biol Eng. 2026 Mar 13;20:70. doi: 10.1186/s13036-026-00661-w (PMC13101387; doi:10.1186/s13036-026-00661-w)
Supplement: Supplementary file 1 — Supplementary Material 1 [file 13036_2026_661_MOESM1_ESM.docx]

**Enhancing Red Blood Cell Compatibility: Mimicking O-Negative RBC Compatibility Using a Trispecific Triabody as a Blocking Fragment for Blood Group Antigens**

^1^Saleha Hafeez and ^1,2^Muhammad Asghar^1,2,3,4^*

^1^Department of Biomedicine, Atta-Ur-Rahman School of Applied Biosciences, National University of Sciences and Technology, H-12 Sector, Islamabad 44000, Pakistan

^2^Department of Biomedical Engineering, School of Mechanical and Manufacturing Engineering, National University of Sciences & Technology, H-12 Sector, Islamabad 44000, Pakistan.

^3^Department of Biology, Lund University Sweden

^4^Department of Sports Sciences and Clinical Biomechanics, University of Southern Denmark.

*Corresponding Author: Muhammad Asghar

Email: [muhammad.asghar@biol.lu.se](mailto:muhammad.asghar@biol.lu.se), [muhammad.asghar@smme.nust.edu.pk](mailto:muhammad.asghar@smme.nust.edu.pk)

**Supplementary Materials**

All the software and web servers used in this study were as follows: I-TASSER web server, PyMOL 3.1, ProtParam tool, PlayMolecule web server, Glycam web server, Llamanade web server, SwissDock web server, GROMACS 2024.2, LZerD web server and Ambertools24.

All the chemicals used were of analytical grade and used as it is without further purification.

Screened blood types A+, B+, O+, AB+, A−, B−, O−, and AB− were provided by NUST ASAB Diagnostics Lab Islamabad and British Lab Multan, Pakistan.

Specific molecular biology products used in study were as follows: Bio Basic prestained mid-range (20-120 kDa) protein ladder, Thermo Fisher HisPur Ni^2+^-NTA resin, Cloud-Clone anti-histidine and HRP-conjugated anti-rabbit antibodies and Bio Basic and SolarBio TMB staining kit, Biosynth blood group A and B trisaccharide, Cloud-Clone recombinant antigen-Rh(D). Thermo Fisher Pierce protein A/G coated plates.

**
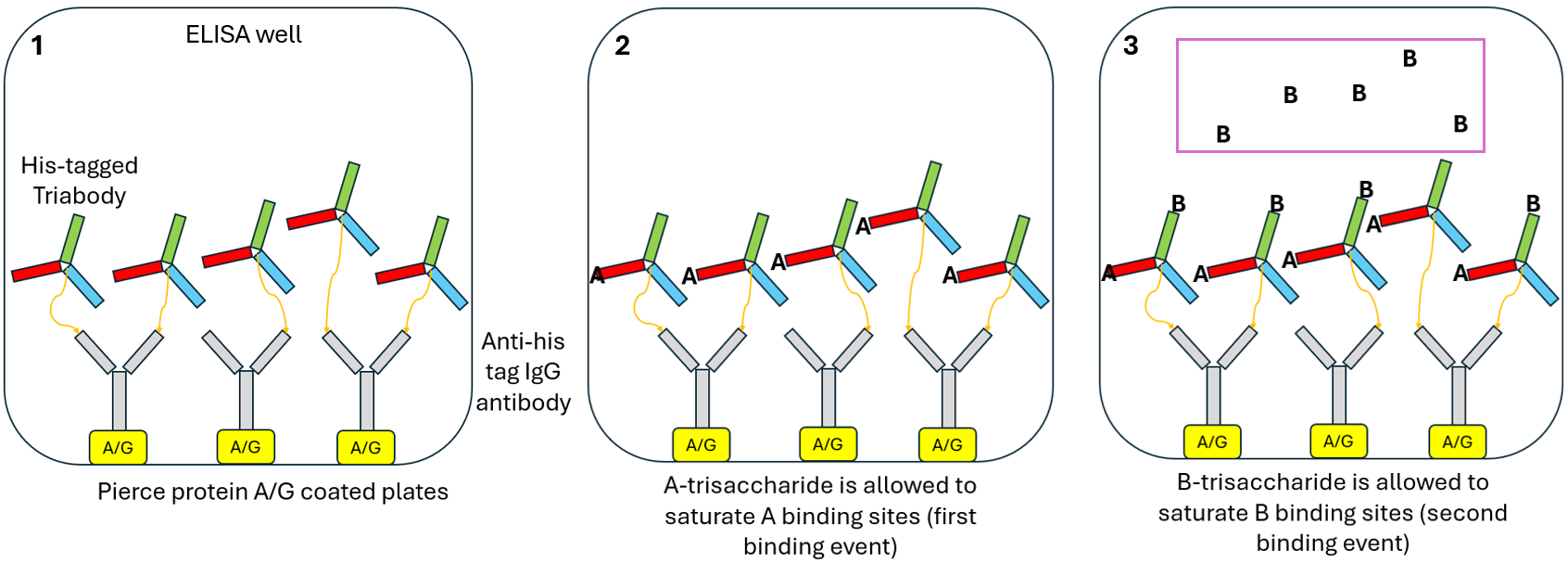
**

**
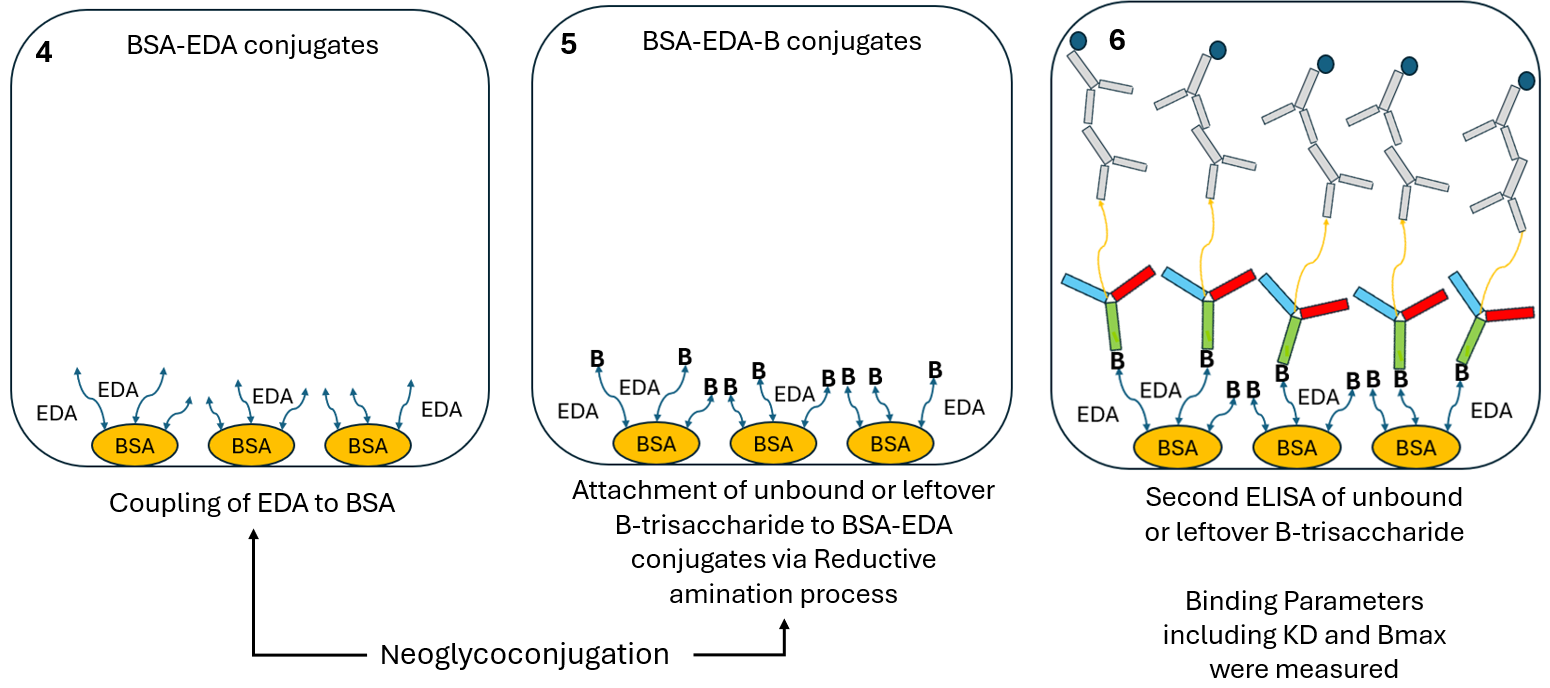
**

**
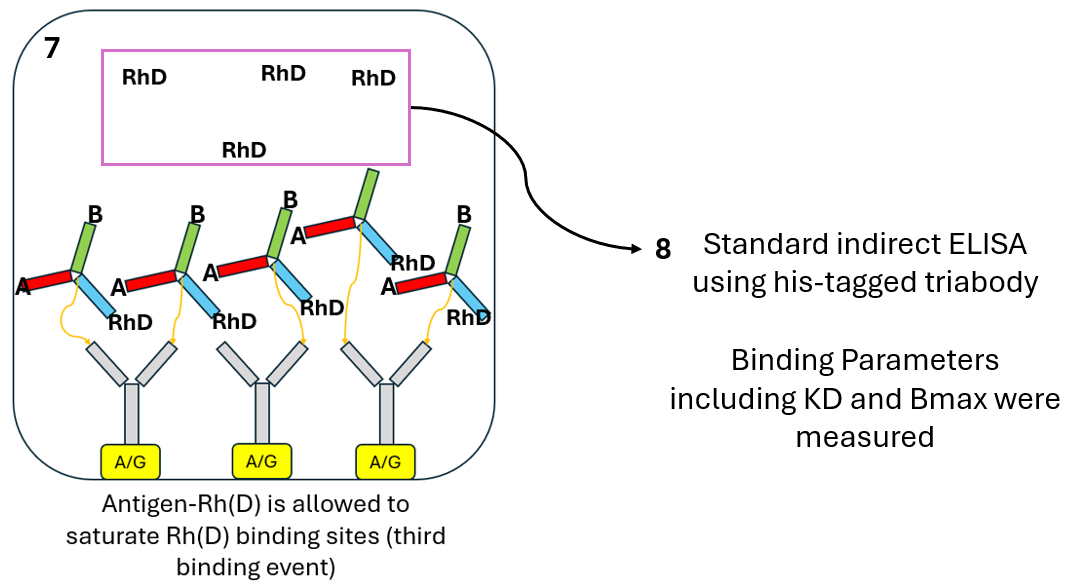
**

**Figure S1:** An overview of the process used for determining binding parameters K_D_ and B_max_ using free antigens.

**Determination of Optimal Antigen and Trisaccharide Concentrations for ELISA-Based Experiments**

An initial test was conducted to determine the optimal concentrations of A and B trisaccharides, as well as the Rh(D) antigen, to cover a wide range of triabody dilutions from 0.2 nM to 100 µM (2-fold serial dilutions). An ELISA experiment was performed, similar to that shown in Supplementary Figure S1. In this experiment, only two triabody concentrations were used: 0.2 nM and 100 µM. The optical density (OD) of leftover or unbound antigens was measured. Optimal concentrations were selected based on detectable OD readings; specifically, the antigen concentration was chosen so that an OD signal was detectable at the highest triabody concentration (100 µM), corresponding to the lowest dilution.

**Table S1:** Concentrations of A-trisaccharide, B-trisaccharide and antigen Rh(D) used for determining intramolecular binding cooperativity of triabody.

| **Trisaccharide/Antigen** | **Concentration**  **(μM) Tri/Ag** | **Lowest Concentration (μM) Triabody** | **OD** | **Highest Concentration (μM)** | **OD** |
| --- | --- | --- | --- | --- | --- |
| A | 100 | 0.0002 | 1.390 | 100 | 0.031 |
| **A** | **50** | **0.0002** | **1.390** | **100** | **0.002** |
| A | 25 | 0.0002 | 1.390 | 100 | 0* |
| A | 12.5 | 0.0002 | 1.330 | 100 | 0* |
| B | 100 | 0.0002 | 1.390 | 100 | 0.023 |
| **B** | **50** | **0.0002** | **1.390** | **100** | **0.001** |
| B | 25 | 0.0002 | 1.390 | 100 | 0* |
| B | 12.5 | 0.0002 | 1.320 | 100 | 0* |
| Rh(D) | 1 | 0.0003 | 1.230 | 100 | 0.007 |
| Rh(D) | 0.5 | 0.0003 | 1.160 | 100 | 0.006 |
| **Rh(D)** | **0.25** | **0.0003** | **1.030** | **100** | **0.001** |
| Rh(D) | 0.125 | 0.0003 | 0.590 | 100 | 0* |

*****Not detectable

**
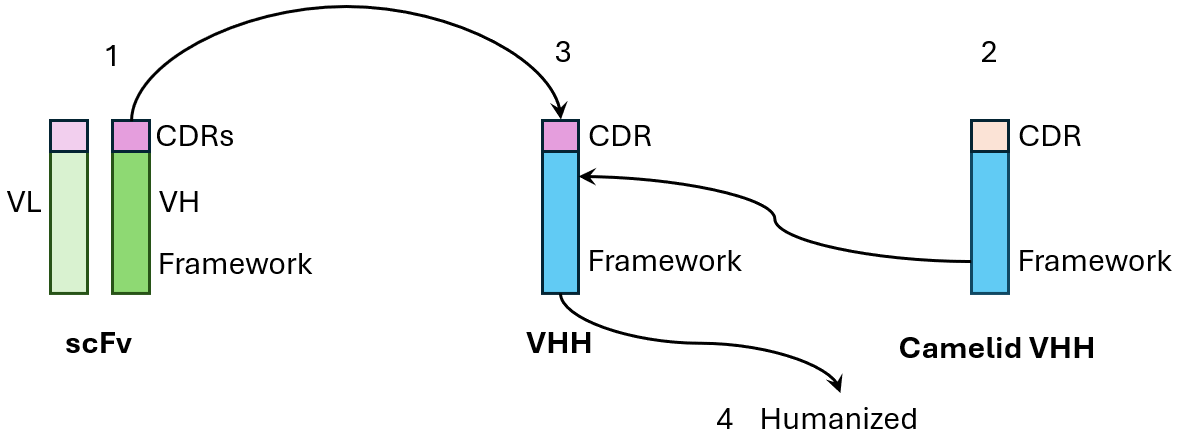
**

**Figure S2:** CDR-grafted humanized nanobody VHH.

**Table S2:** Dissociation pH of several blood group nanobodies, scFv and triabody.

| **Triabody** | **Dissociation pH** | **Nanobody** | **Dissociation pH** | **scFv** | **Dissociation pH** |
| --- | --- | --- | --- | --- | --- |
| A | 6.7 | - | - | - | - |
| B | 7.1 | - | - | - | - |
| RhD | 9.12 | - | - | - | - |
| - | - | - | - | Rh(D)  (AAC13488.1) VL  (AAB22940.1)  VH | 8.54 |
| - | - | A1 (1JV5) | 7.91 | - | - |
| - | - | A2* | 8.3 | - | - |
| - | - | B1* | 7.83 | - | - |
| - | - | B2 (AAK07095.1) | 8.02 | - | - |
| - | - | B3 (AAK07096.1) | 8.21 | - | - |
| - | - | Rh(D)1 (AAB22940.1) | 7.90 | - | - |
| - | - | Rh(D)2  (AAC13437.1) | 7.97 | - | - |
| - | - | Rh(D)3 (AAC13447.1) | 8.19 | - | - |

*Sequences taken from research article Santos‐Esteban and Curiel‐Quesada (2001).

**Table S3:** Selected blocking fragments for studying different binding orders in ELISA-based experiments.

| **Binding Sequence** | **1^st^ Blocking Fragment** | **Dissociation pH** | **2^nd^ Blocking Fragment** | **Dissociation pH** |
| --- | --- | --- | --- | --- |
| A→B→RhD | B1 | 7.83 | Rh(D) | 8.54 |
| A→RhD→B | Rh(D)3 | 7.9 | B3 | 8.21 |
| B→A→RhD | A1 | 7.91 | Rh(D) | 8.54 |
| B→RhD→A | Rh(D)3 | 7.9 | A2 | 8.3 |
| RhD→A→B | A1 | 7.91 | B3 | 8.21 |
| RhD→B→A | B1 | 7.83 | A2 | 8.3 |

**
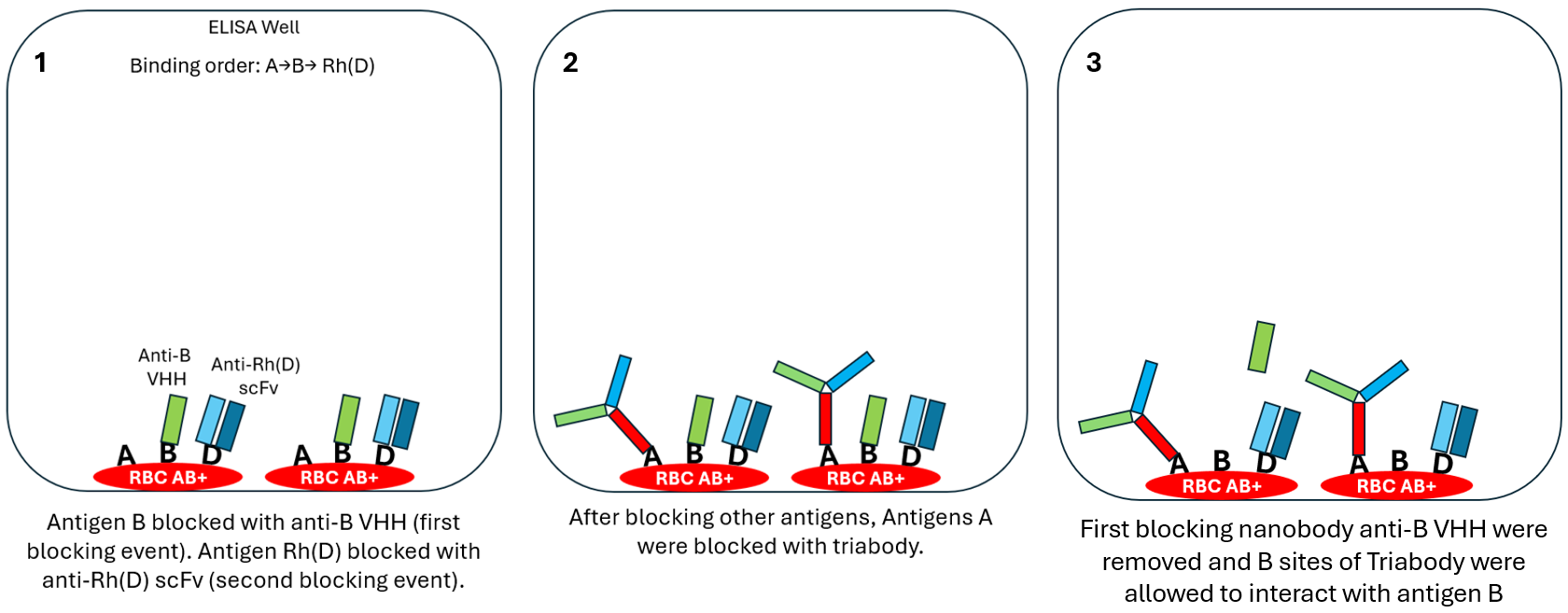
**

**
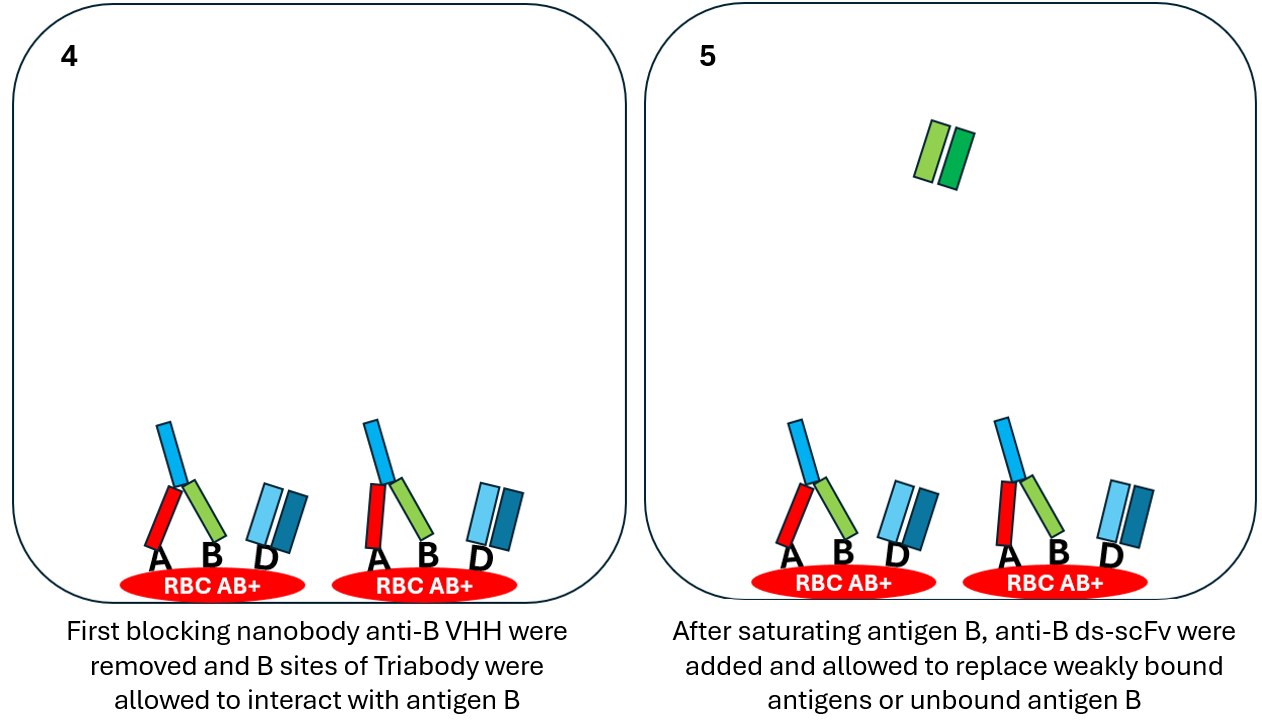
**

**
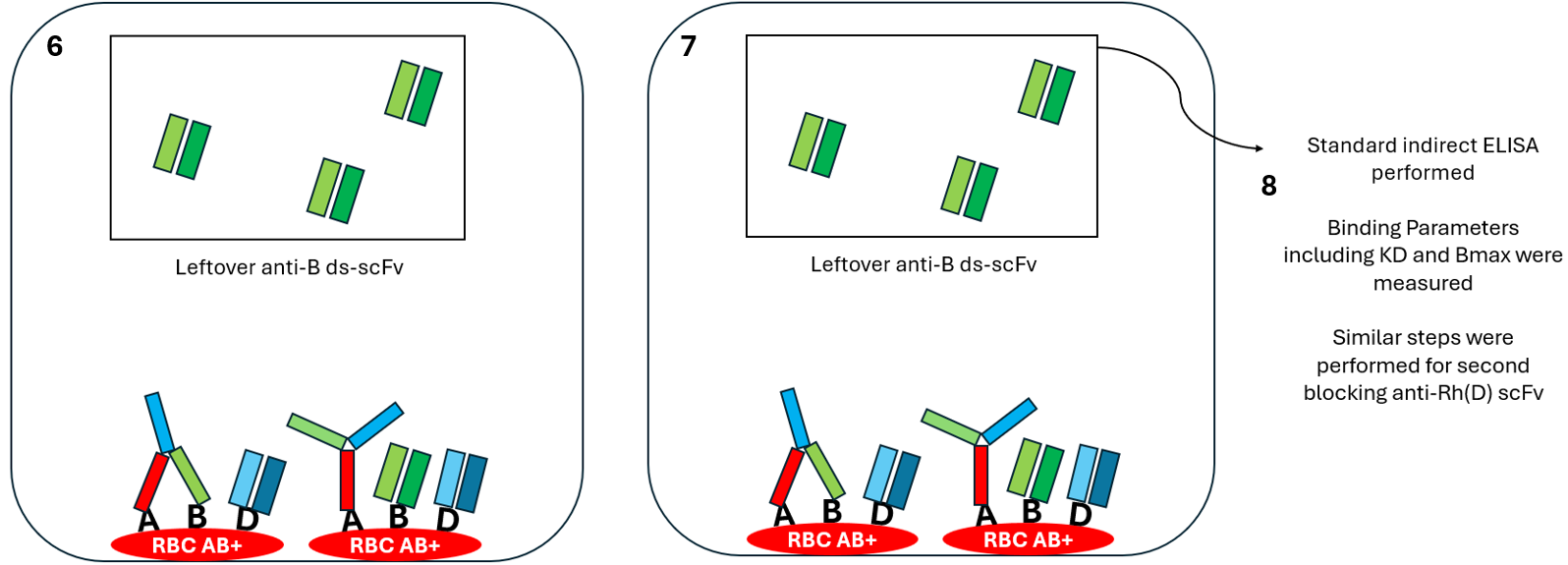
**

**Figure S3:** An overview of the process used for determining binding parameters K_D_ and B_max_ for binding order A→**B**→ **Rh(D)** using RBC-bound antigens.


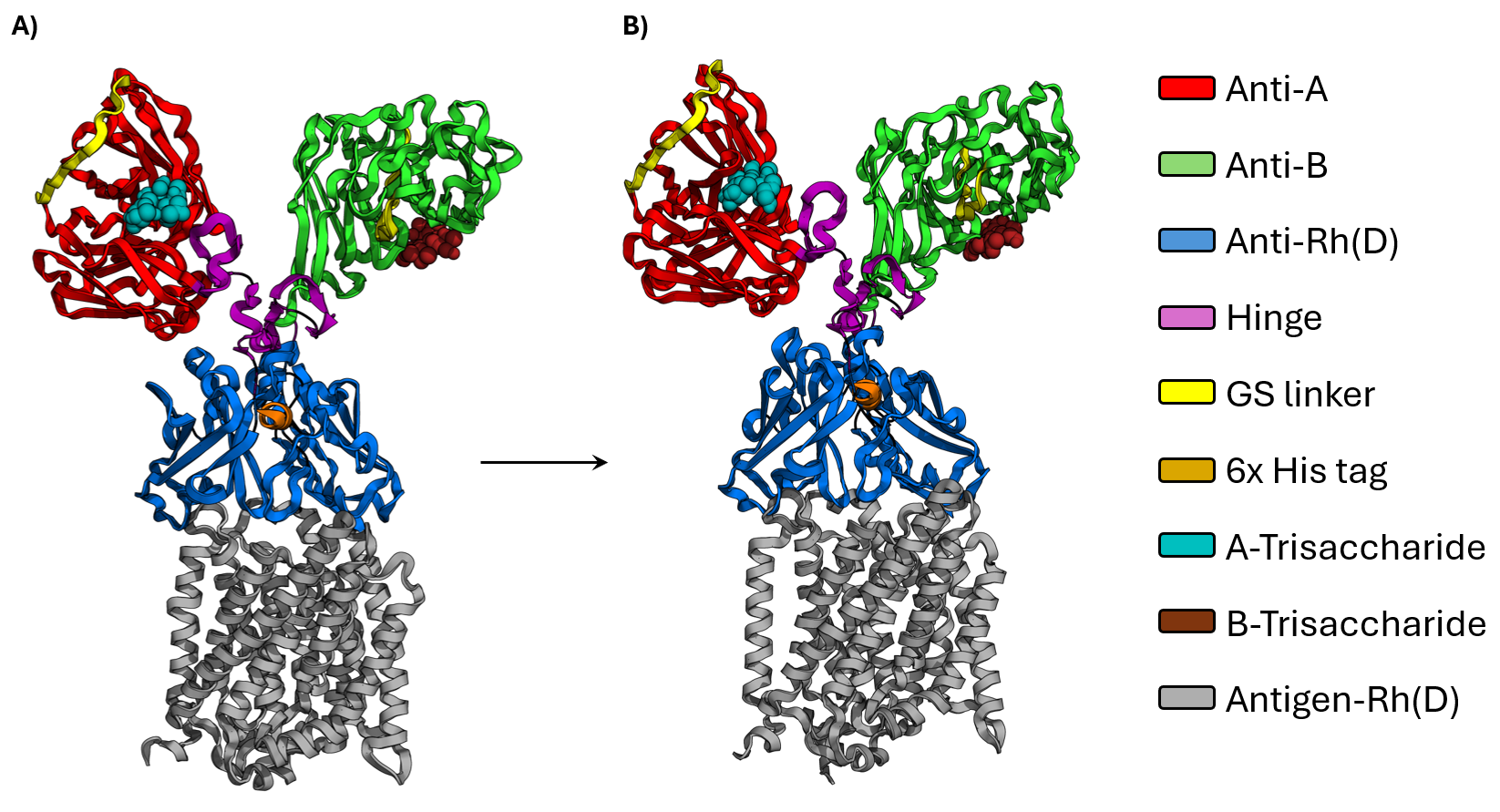


**Figure S4:** Snapshots of MD trajectories taken at the start and end of simulation of binding sequence A→B→ Rh(D). A) Snapshot taken at the start (0 ns) of simulation. B) Snapshot taken at the end (150 ns) of simulation. All complexes including anti-A ds-scFv with antigen A, anti-B ds-scFv with antigen B and anti-Rh(D) Fv with antigen Rh(D) were stable throughout the simulations. Antigens A and B are shown as light blue and dark red spheres respectively. PyMOL 3.1 was used to generate high-resolution images.

**
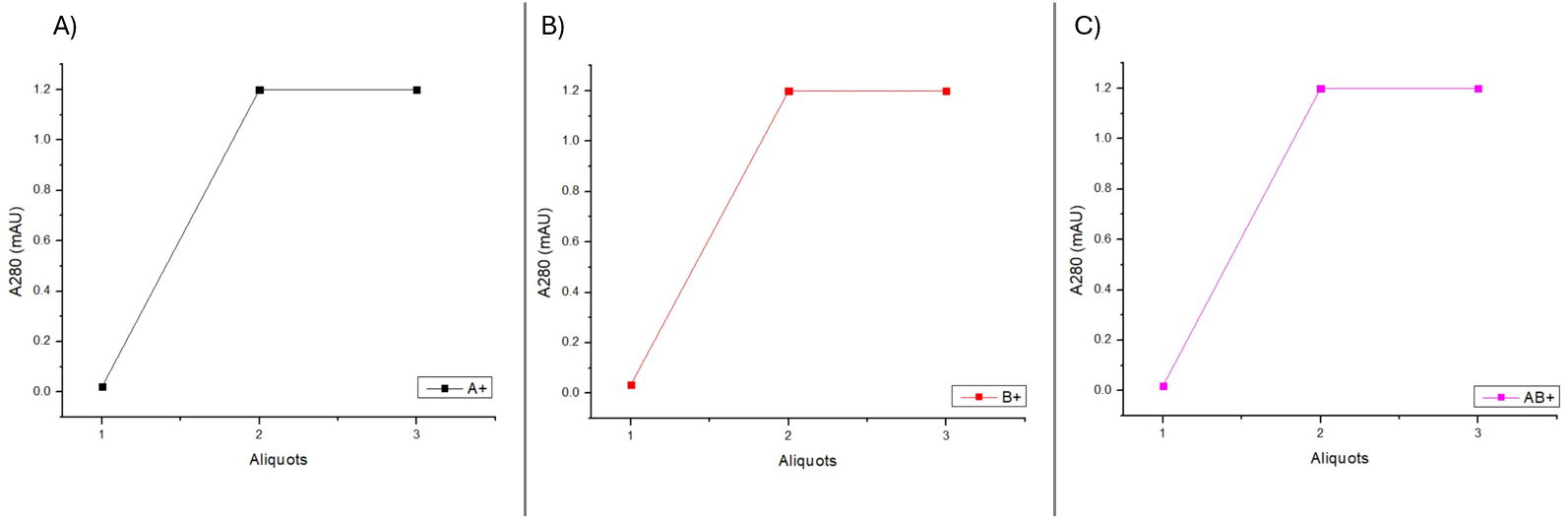
**

**
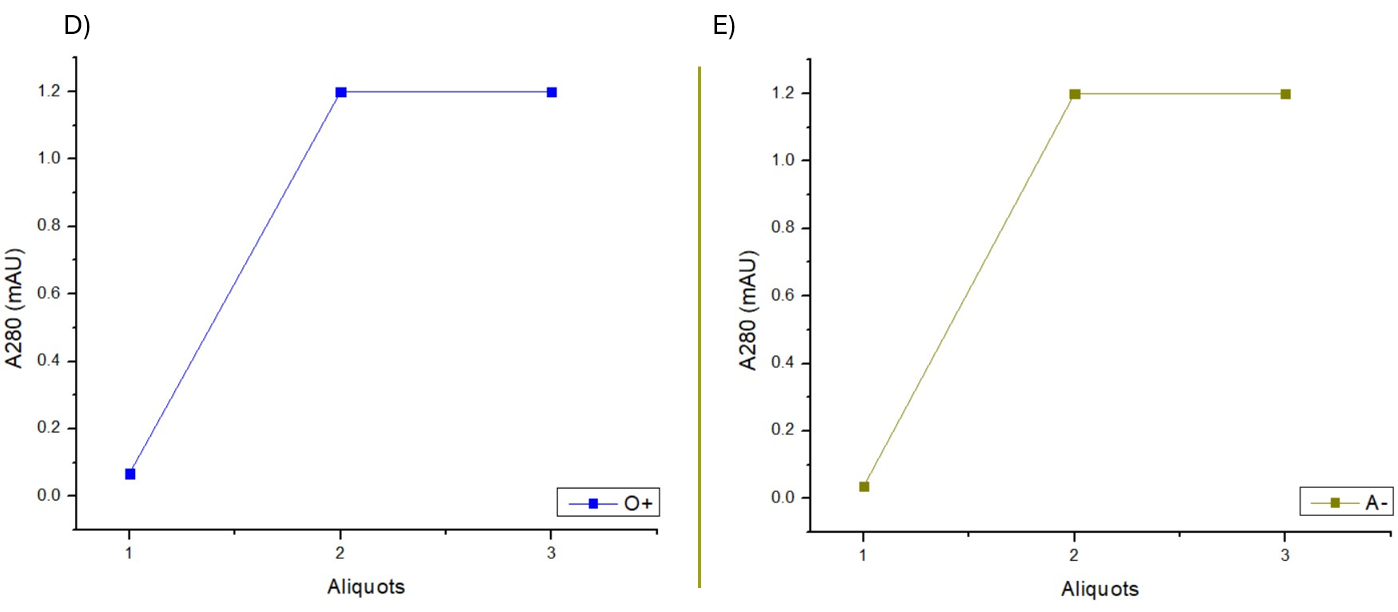
**

**
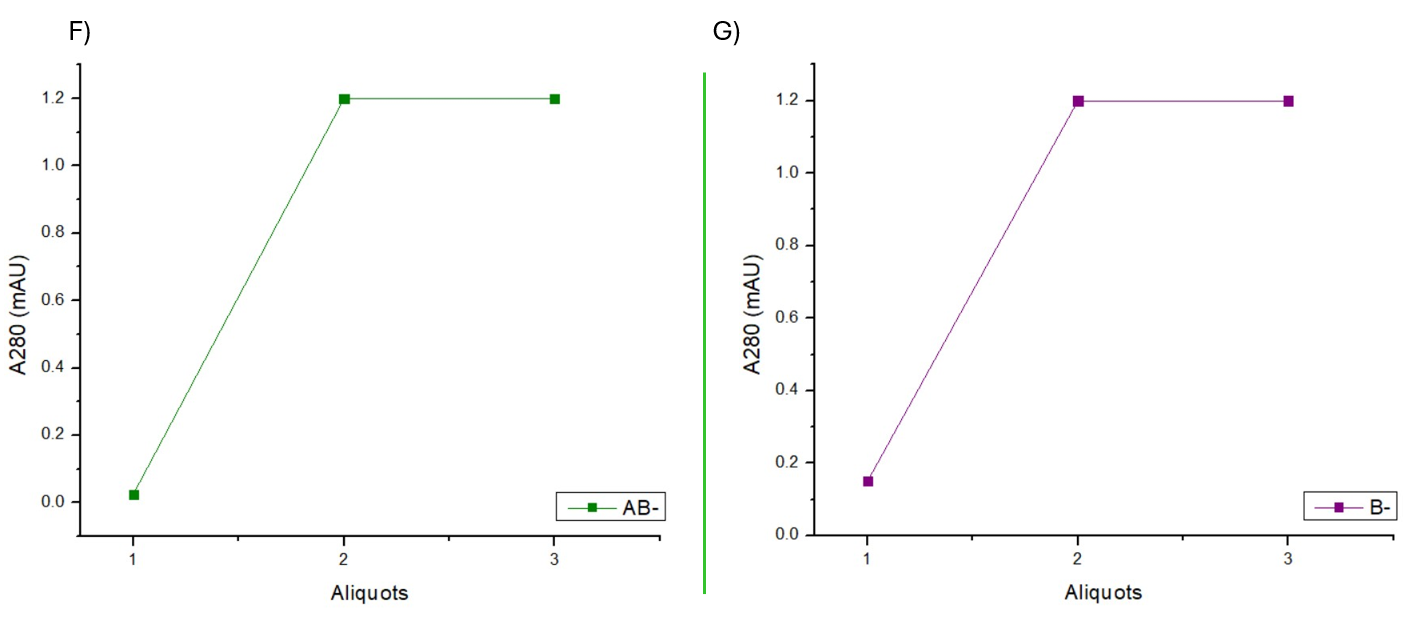
**

**Figure S5:** Graphs show the gradual decrease in the concentrations of triabody in the supernatant containing A) A+, B) B+, C) AB+, D) O+, E) A-, F) AB- and G) B- RBCs.

**
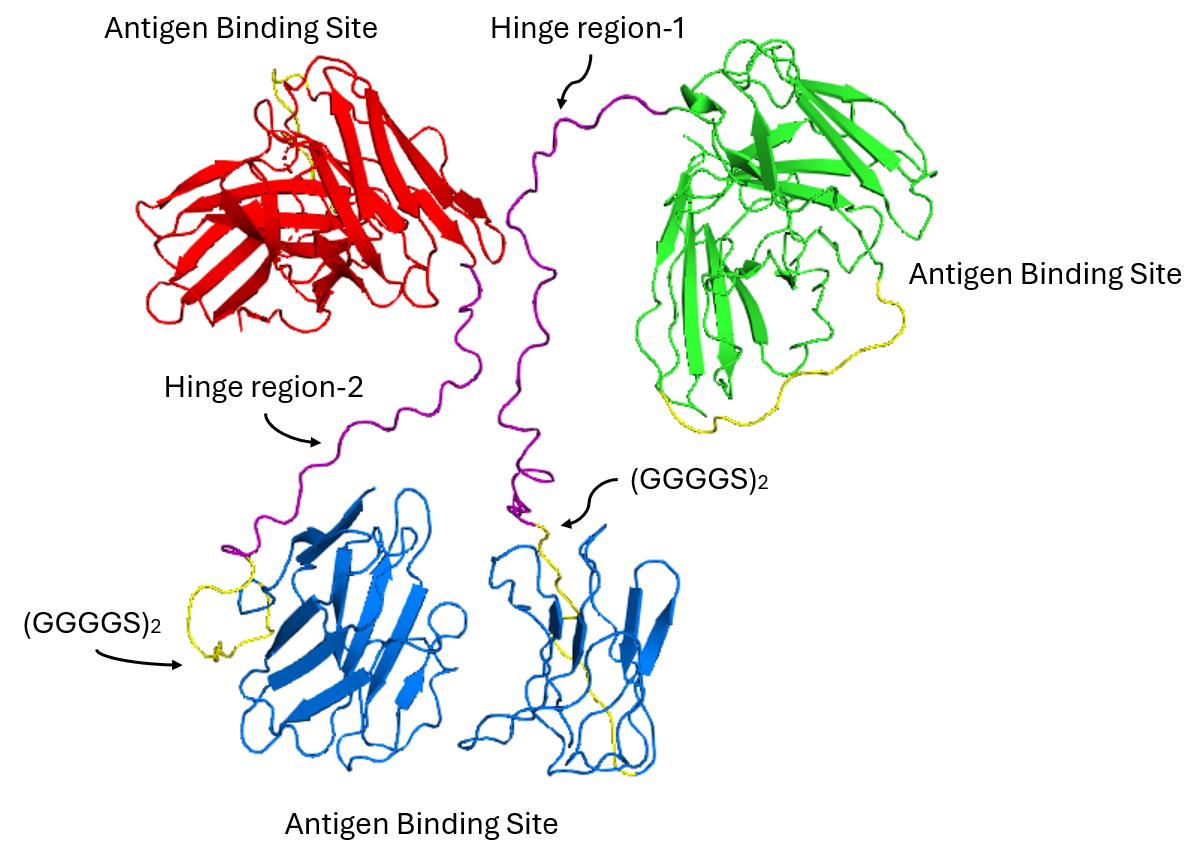
**

**Figure S6:** A model of triabody made from fusion proteins of combination two. A GS linker (yellow) was added between hinge region (purple) and variable chains (blue) of anti-Rh(D) to allow the fusion proteins to reorient and expose the anti-Rh(D) binding sites in final structure of triabody.
