# Supplementary material for: Enhancing red blood cell compatibility: in vitro hemagglutination prevention using a trispecific triabody as a blocking fragment for blood group antigens
Source: J Biol Eng. 2026 Mar 13;20:70. doi: 10.1186/s13036-026-00661-w (PMC13101387; doi:10.1186/s13036-026-00661-w)
Supplement: Supplementary file 2 — Supplementary Material 2 [file 13036_2026_661_MOESM2_ESM.docx]

**Enhancing Red Blood Cell Compatibility: In Vitro Hemagglutination Prevention Using a Trispecific Triabody as a Blocking Fragment for Blood Group Antigens**

^1^Saleha Hafeez and ^1,2^Muhammad Asghar^1,2,3,4^*

^1^Department of Biomedicine, Atta-Ur-Rahman School of Applied Biosciences, National University of Sciences and Technology, H-12 Sector, Islamabad 44000, Pakistan

^2^Department of Biomedical Engineering, School of Mechanical and Manufacturing Engineering, National University of Sciences & Technology, H-12 Sector, Islamabad 44000, Pakistan.

^3^Department of Biology, Lund University Sweden

^4^Department of Sports Sciences and Clinical Biomechanics, University of Southern Denmark.

*Corresponding Author: Muhammad Asghar

Email: [muhammad.asghar@biol.lu.se](mailto:muhammad.asghar@biol.lu.se), [muhammad.asghar@smme.nust.edu.pk](mailto:muhammad.asghar@smme.nust.edu.pk)

**Supplementary Materials**

All the software and web servers used in this study were as follows: I-TASSER web server, PyMOL 3.1, ProtParam tool, PlayMolecule web server, Glycam web server, Llamanade web server, SwissDock web server, GROMACS 2024.2, LZerD web server and Ambertools24.

All the chemicals used were of analytical grade and used as it is without further purification.

Screened blood types A+, B+, O+, AB+, A−, B−, O−, and AB− were provided by NUST ASAB Diagnostics Lab Islamabad and British Lab Multan, Pakistan.

Specific molecular biology products used in study were as follows: Bio Basic prestained mid-range (20-120 kDa) protein ladder, Thermo Fisher HisPur Ni^2+^-NTA resin, Cloud-Clone anti-histidine and HRP-conjugated anti-rabbit antibodies and Bio Basic and SolarBio TMB staining kit, Biosynth blood group A and B trisaccharide, Cloud-Clone recombinant antigen-Rh(D). Thermo Fisher Pierce protein A/G coated plates.

**Gene constructs**

For expression in pET-28a (+), the first fusion protein consisting of genes encoding anti-A ds-scFv and the VH chain of anti-Rh(D) dsFv was modified to include the genes encoding the OmpA signal peptide at the N-terminus and a 6X histidine tag at the C-terminus. This gene construct was then inserted between the NcoI and XhoI restriction sites. Similarly, the gene construct of the second fusion protein of the triabody consisting of genes encoding anti-B ds-scFv and the VL chain of anti-Rh(D) dsFv, was placed between the BamHI and XhoI restriction sites of the pET-21 (+) plasmid after the genes encoding the ribosomal binding site (RBS) and OmpA signal peptide at the N-terminal region were added.


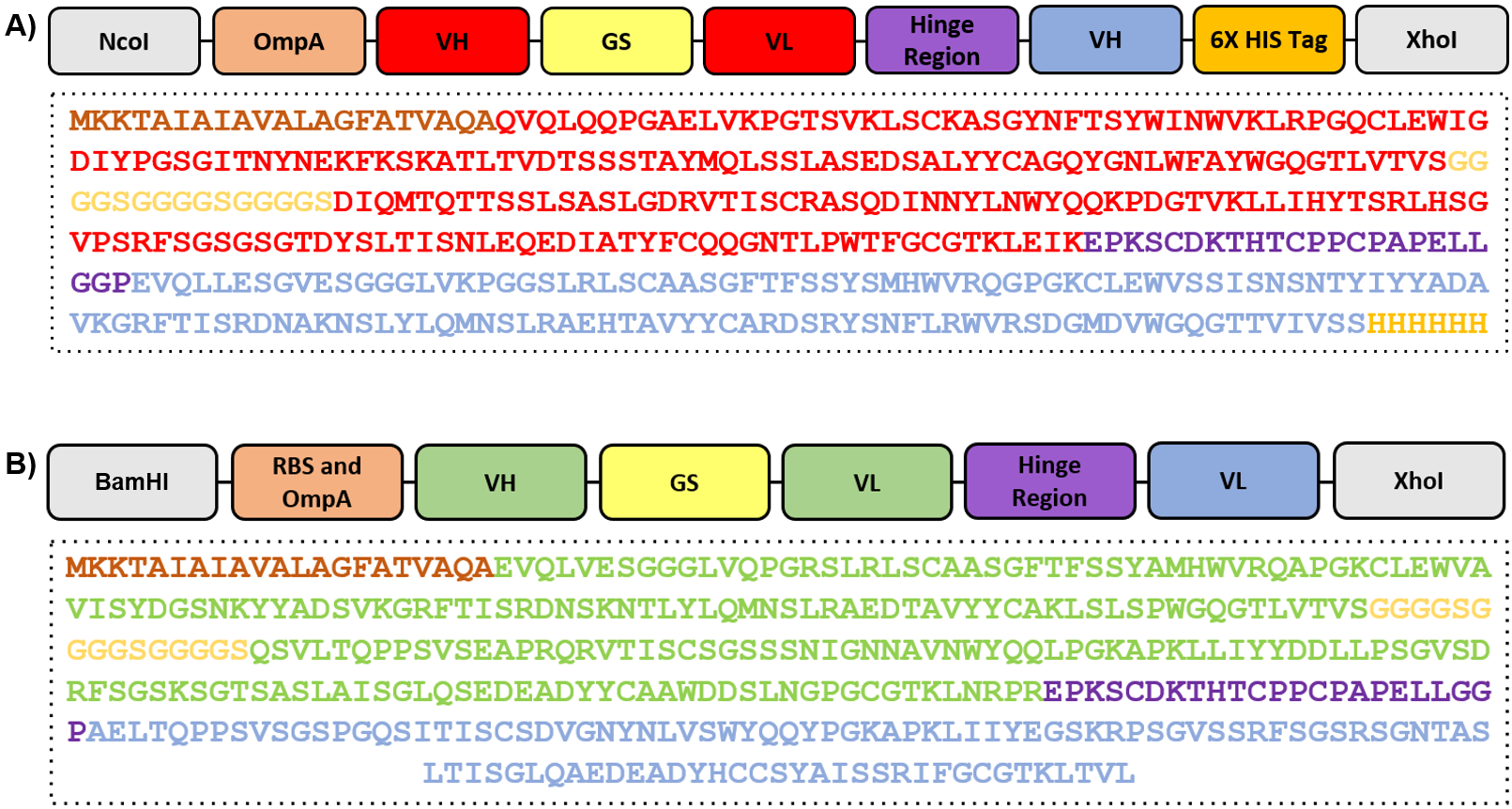


**Figure S1**: Schematic diagrams of recombinant fusion protein constructs. A) Amino acid sequence of the first fusion protein encoded by a gene construct inserted into the pET-28a (+) plasmid. The protein of interest includes variable chains of anti-A ds-scFv fused to the VH chain of anti-Rh(D) dsFv, followed by a 6X-His tag. B) Amino acid sequence of the second fusion protein encoded by a gene construct inserted into the pET-21 (+) plasmid. The protein of interest consists of variable chains of anti-B ds-scFv fused to the VL chain of anti-Rh(D) dsFv.

**Purification**

The first step of purification was performed using a batch method. Briefly, 600 µL of Ni^2+^-NTA resin equilibrated with resuspension buffer (50 mM Tris, 154 mM NaCl, pH 7.4) was added to the clarified crude sample and incubated overnight at 18 °C under continuous shaking. The following day, the resin was washed several times with wash buffer (50 mM Tris, 20 mM imidazole, 154 mM NaCl, pH 7.4) to remove unbound and weakly bound proteins. Finally, the His-tagged proteins were eluted by incubating the resin with 1 mL of elution buffer (50 mM Tris, 250 mM imidazole, 200 mM NaCl, pH 8.0) for 30 min at room temperature.

In the second step the Ni^2+^-NTA purified product was loaded onto a 10% Native PAGE gel. After the run, the protein ladder was used as a reference and the gel was divided into sections corresponding to molecular weights ranging from approximately 70-84 kDa, and 85-100 kDa. Each gel piece was placed in a 2 ml Eppendorf tube containing 0.5 ml Tris-buffered saline (TBS) (50 mM Tris, 154 mM NaCl, pH 7.4) and crushed with a Teflon pestle. The tubes were incubated at 37 °C with shaking for 24 hours, followed by centrifugation and resuspension every 3 hours. After 24 hours, the tubes were centrifuged at 5,000 rpm, and the supernatant was analyzed via the western blotting.

The third purification step involved the sequential purification of triabodies using blood type A−, B− and O+ RBCs. The previously purified product (0.5 ml) was incubated with blood type A RBCs (10 μl) for 30 mins to purify triabodies with functional A binding sites. After incubation, unbound triabodies were removed by washing with TBS buffer A (pH 7.4). The bound triabodies were then detached by using 0.25 ml of Glycine-HCl buffer B (50 mM Glycine, pH 3.5). The final eluate-A was neutralized by using 1 ml of TBS buffer C (pH 8.5). Eluate-A was subsequently used to purify triabodies with functional Rh(D) binding sites. The resulting eluate-Rh(D) eluate served as the starting material for the purification of triabodies with functional B binding sites. The final product was lyophilized and stored at −70 °C.

Size-exclusion chromatography (SEC) was performed using a Superdex 200 Increase 10/300 GL column equilibrated with resuspension buffer (50 mM Tris, 154 mM NaCl, pH 7.4). Lyophilized samples were resuspended in TBS buffer at a concentration of 1 mg/ml. Chromatography was carried out on a Bio-Rad NGC Quest 10 Plus system at a constant flow rate of 0.5 mL/min, with a maximum injection volume of 500 µL per run. Fractions were collected at 0.5 mL intervals and analyzed by non-reducing SDS-PAGE.

**Immunocytochemistry (ICC)**

Briefly, 5 μl of RBCs were incubated for 30 mins under gentle shaking in 0.5 ml of 250 μM triabody solution. To prevent hemagglutination due to primary and secondary antibodies, triabody-coated RBCs were first diluted in TBS buffer (pH 7.4) and then smeared onto glass slides. The smears were air dried and fixed with methanol for 1 min. Next, primary rabbit anti-his tag polyclonal antibodies (1:1000 dilution) were added, and the samples were incubated at room temperature for one hour. Following incubation, the slides were washed with TBS buffer (pH 7.4) before being incubated with a secondary HRP-conjugated caprine anti-rabbit IgG polyclonal antibody (1:10000 dilution). After incubation, the slides were washed with TBS buffer (pH 7.4), and staining was performed via 3,3′,5,5′-Tetramethylbenzidine (TMB) staining method as described by Woiszwillo (1991). To prevent hemolysis, the smears were exposed to the working TMB precipitating solution (2 mM TMB, 0.1% H_2_O_2_ and precipitating polymers: 0.1% alginic acid, 0.1% methyl vinyl ether/ maleic anhydride copolymer, 0.1% dextran sulfate and 0.3% carrageenan) for just 5 secs and then washed with TBS buffer (pH 7.4). This process was repeated until sufficient color was developed for optical microscopy.

Woiszwillo JE. TMB formulation for soluble and precipitable HRP-ELISA. US Patent 5,006,461. United States; 1991.

**Potentiator-enhanced hemagglutination (PEH) assay**

A potentiator-enhanced hemagglutination assay was performed using ficin to reduce the zeta potential of red blood cells (RBCs) and enhance agglutination sensitivity. Briefly, 30 µl of AB+ RBCs were treated with ficin (0.1% w/v) and incubated for 10 min at 37 °C. The ficin-treated RBCs were then divided into three 10 µl aliquots. Two of these aliquots were incubated with triabody fractions AE3-B1 and AE3-B2, respectively, while the third aliquot, consisting of RBCs without any triabody, served as a negative control. All samples were incubated for 30 min at room temperature, and hemagglutination was assessed microscopically.

**
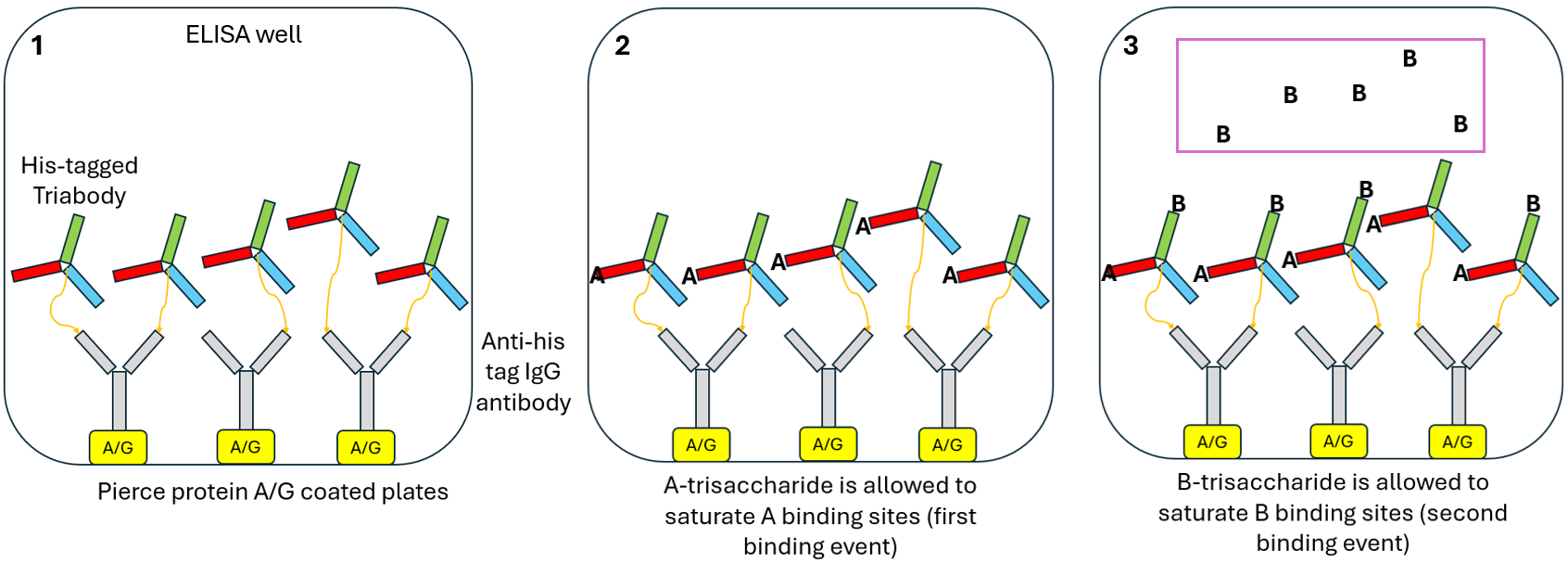
**

**
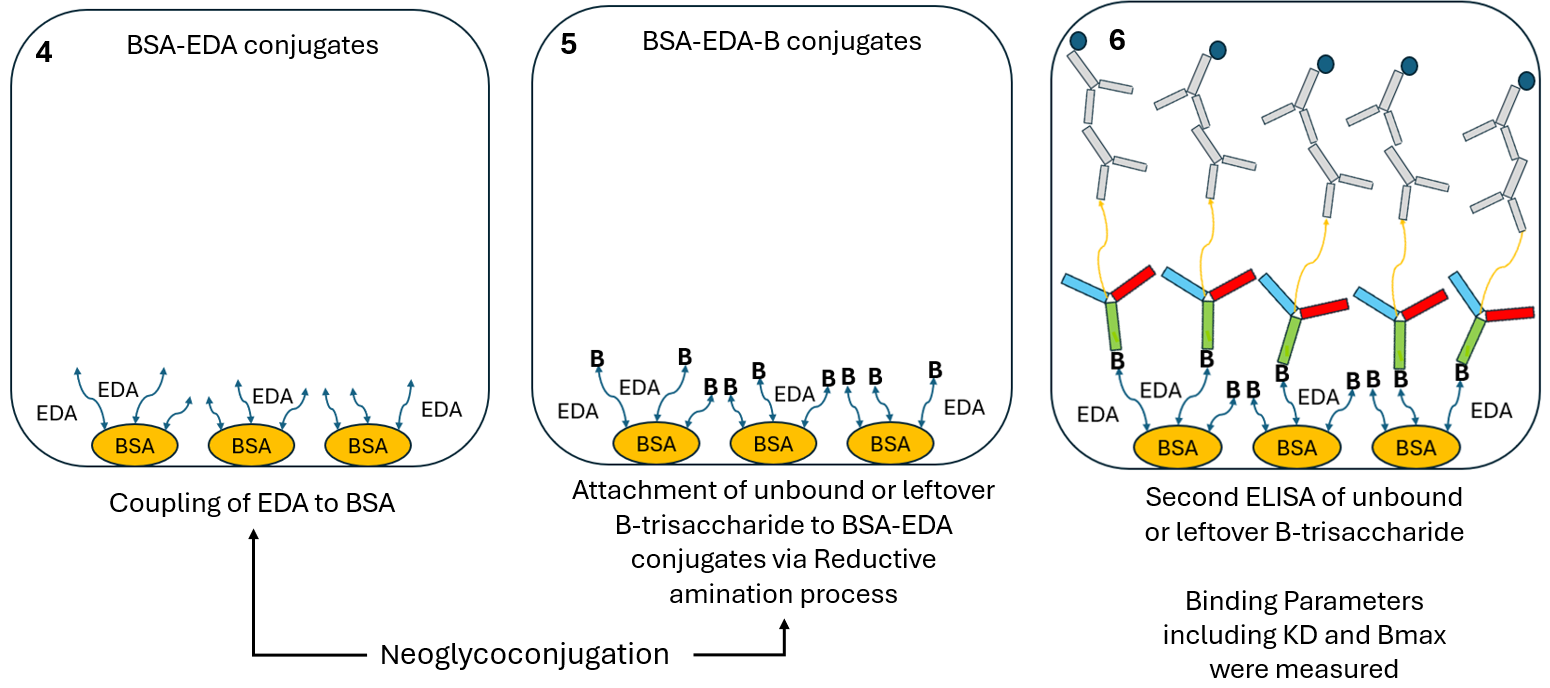
**

**
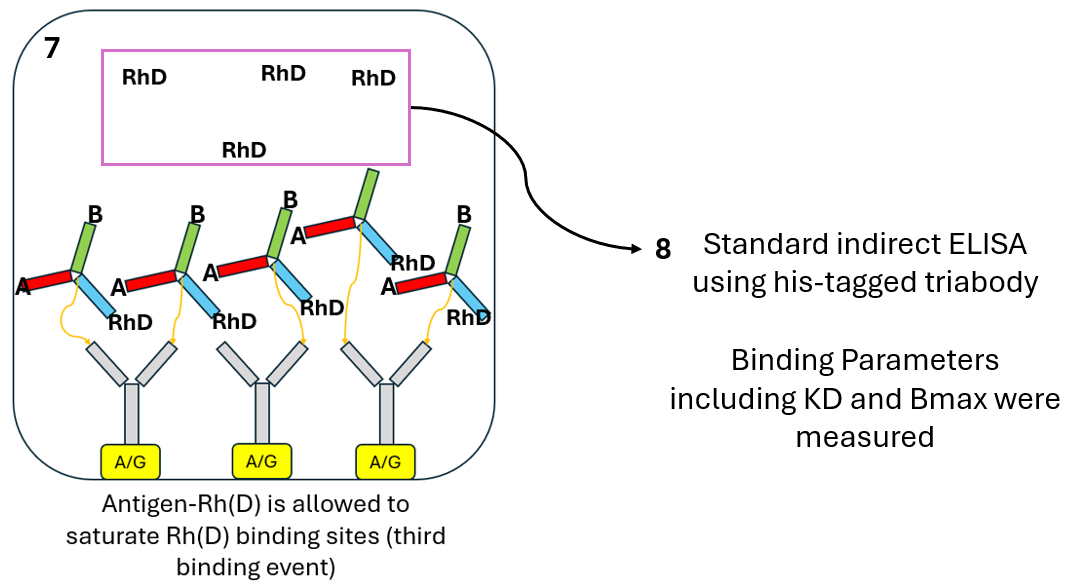
**

**Figure S2:** An overview of the process used for determining binding parameters K_D_ and B_max_ using free antigens.

**Determination of Optimal Antigen and Trisaccharide Concentrations for ELISA-Based Experiments**

An initial test was conducted to determine the optimal concentrations of A and B trisaccharides, as well as the antigen Rh(D), to cover a wide range of triabody dilutions from 0.2 nM to 100 µM (2-fold serial dilutions). An ELISA experiment was performed, similar to that shown in Supplementary Figure S1. In this experiment, only two triabody concentrations were used: 0.2 nM and 100 µM. The optical density (OD) of leftover or unbound antigens was measured. Optimal concentrations were selected based on detectable OD readings; specifically, the antigen concentration was chosen so that an OD signal was detectable at the highest triabody concentration (100 µM), corresponding to the lowest dilution.

- Lowest OD corresponds to highest concentration (100 µM, lowest dilution) of triabody. Less antigens were unbound and more antigens were in bound state.
- Highest OD corresponds to highest dilution (2 nM, lowest concentration) of triabody. More antigens were unbound and less antigens were in bound state.


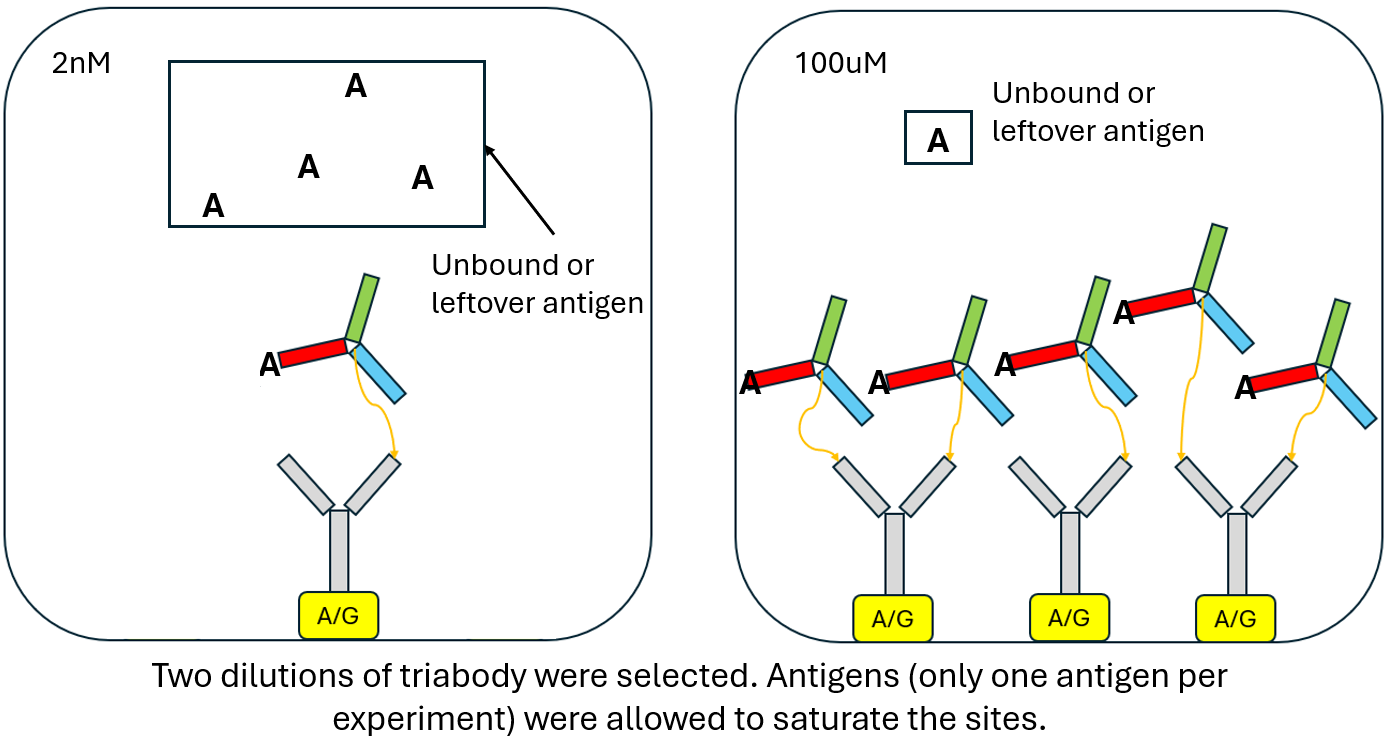

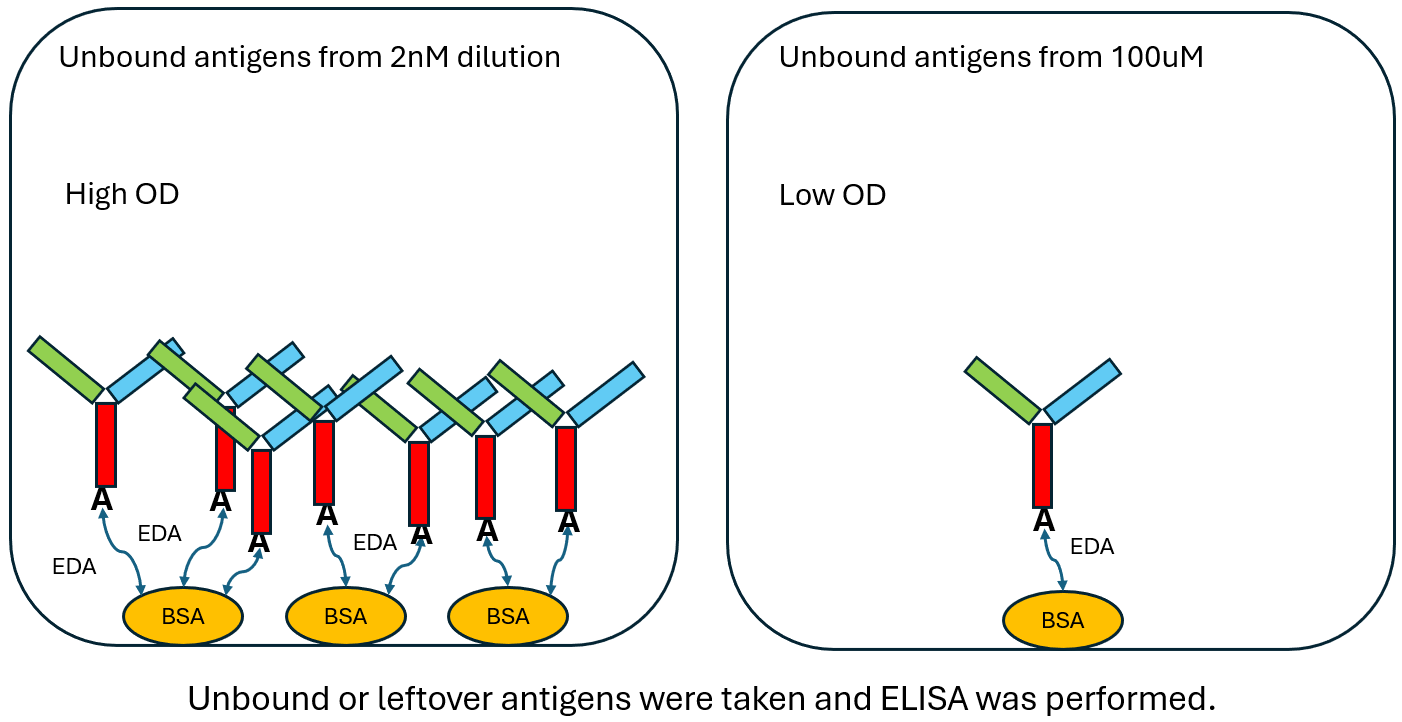


**Figure S3**: An overview of the process used for determining the optimal concentrations of antigens (process of antigen A is shown here).

**Table S1:** Concentrations of A-trisaccharide, B-trisaccharide and antigen Rh(D) used for determining intramolecular binding cooperativity of triabody.

| **Trisaccharide/Antigen** | **Concentration**  **(μM) Tri/Ag** | **Lowest Concentration (μM) Triabody** | **OD** | **Highest Concentration (μM)** | **OD** |
| --- | --- | --- | --- | --- | --- |
| A | 100 | 0.0002 | 1.390 | 100 | 0.031 |
| **A** | **50** | **0.0002** | **1.390** | **100** | **0.002** |
| A | 25 | 0.0002 | 1.390 | 100 | 0* |
| A | 12.5 | 0.0002 | 1.330 | 100 | 0* |
| B | 100 | 0.0002 | 1.390 | 100 | 0.023 |
| **B** | **50** | **0.0002** | **1.390** | **100** | **0.001** |
| B | 25 | 0.0002 | 1.390 | 100 | 0* |
| B | 12.5 | 0.0002 | 1.320 | 100 | 0* |
| Rh(D) | 1 | 0.0003 | 1.230 | 100 | 0.007 |
| Rh(D) | 0.5 | 0.0003 | 1.160 | 100 | 0.006 |
| **Rh(D)** | **0.25** | **0.0003** | **1.030** | **100** | **0.001** |
| Rh(D) | 0.125 | 0.0003 | 0.590 | 100 | 0* |

*****Not detectable

**
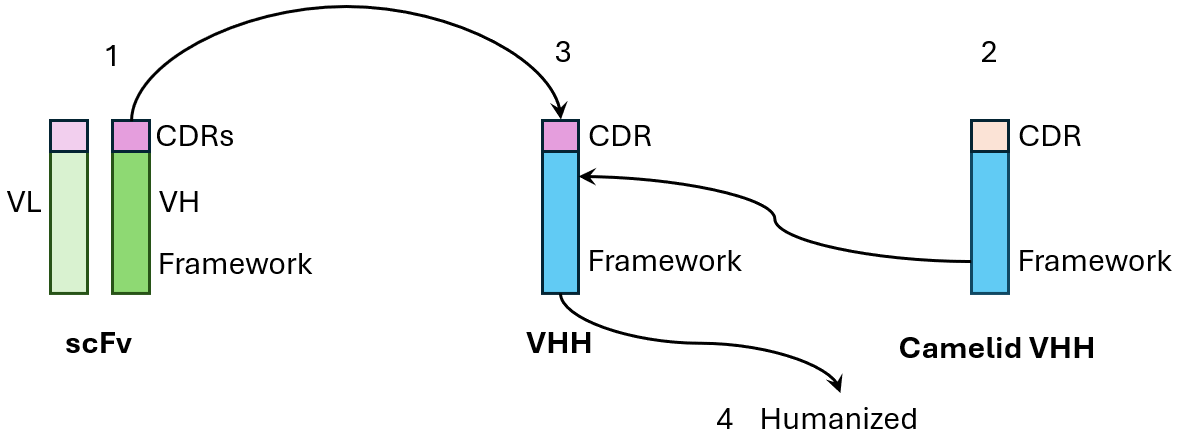
**

**Figure S4:** CDR-grafted humanized nanobody VHH.

**Table S2:** Dissociation pH of several blood group nanobodies, scFv and triabody.

| **Triabody** | **Dissociation pH** | **Nanobody** | **Dissociation pH** | **scFv** | **Dissociation pH** |
| --- | --- | --- | --- | --- | --- |
| A | 6.7 | - | - | - | - |
| B | 7.1 | - | - | - | - |
| RhD | 9.12 | - | - | - | - |
| - | - | - | - | Rh(D)  (AAC13488.1) VL  (AAB22940.1)  VH | 8.54 |
| - | - | A1 (1JV5) | 7.91 | - | - |
| - | - | A2* | 8.3 | - | - |
| - | - | B1* | 7.83 | - | - |
| - | - | B2 (AAK07095.1) | 8.02 | - | - |
| - | - | B3 (AAK07096.1) | 8.21 | - | - |
| - | - | Rh(D)1 (AAB22940.1) | 7.90 | - | - |
| - | - | Rh(D)2  (AAC13437.1) | 7.97 | - | - |
| - | - | Rh(D)3 (AAC13447.1) | 8.19 | - | - |

*Sequences taken from research article Santos‐Esteban and Curiel‐Quesada (2001).

**Table S3:** Selected blocking fragments for studying different binding orders in ELISA-based experiments.

| **Binding Sequence** | **1^st^ Blocking Fragment** | **Dissociation pH** | **2^nd^ Blocking Fragment** | **Dissociation pH** |
| --- | --- | --- | --- | --- |
| A→B→RhD | B1 | 7.83 | Rh(D) | 8.54 |
| A→RhD→B | Rh(D)3 | 7.9 | B3 | 8.21 |
| B→A→RhD | A1 | 7.91 | Rh(D) | 8.54 |
| B→RhD→A | Rh(D)3 | 7.9 | A2 | 8.3 |
| RhD→A→B | A1 | 7.91 | B3 | 8.21 |
| RhD→B→A | B1 | 7.83 | A2 | 8.3 |

**
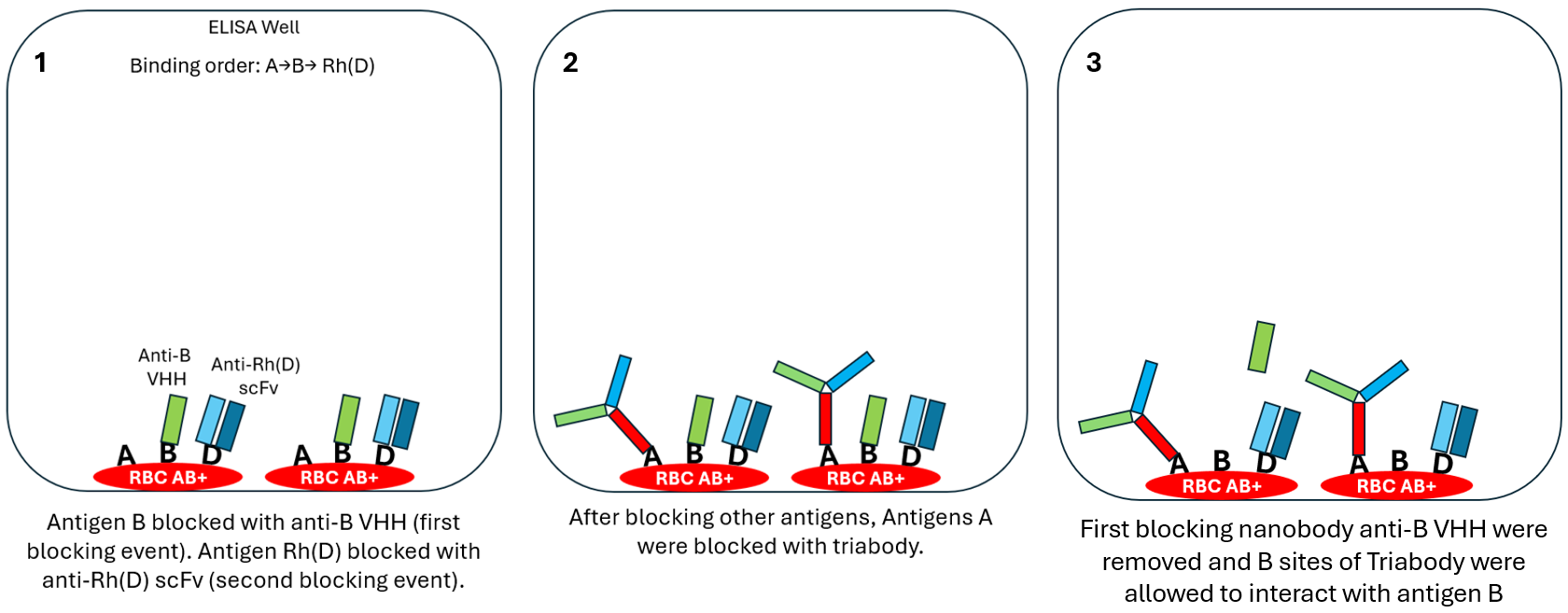
**

**
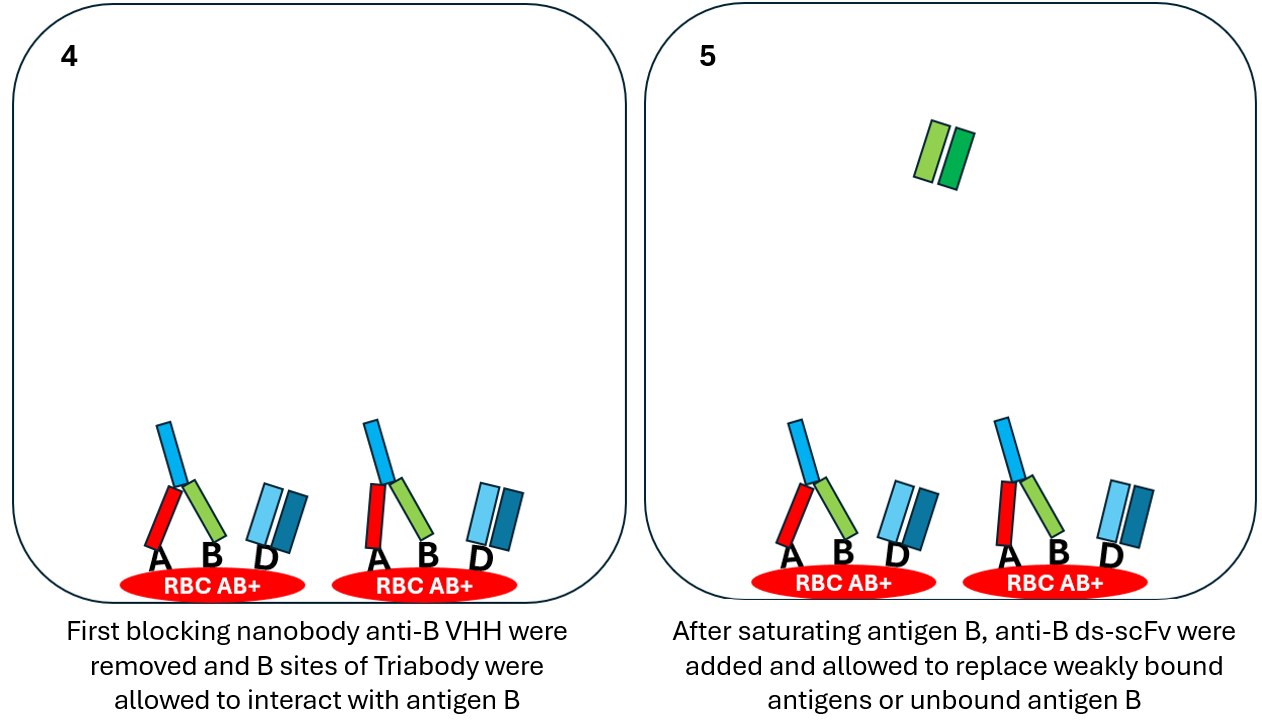
**

**
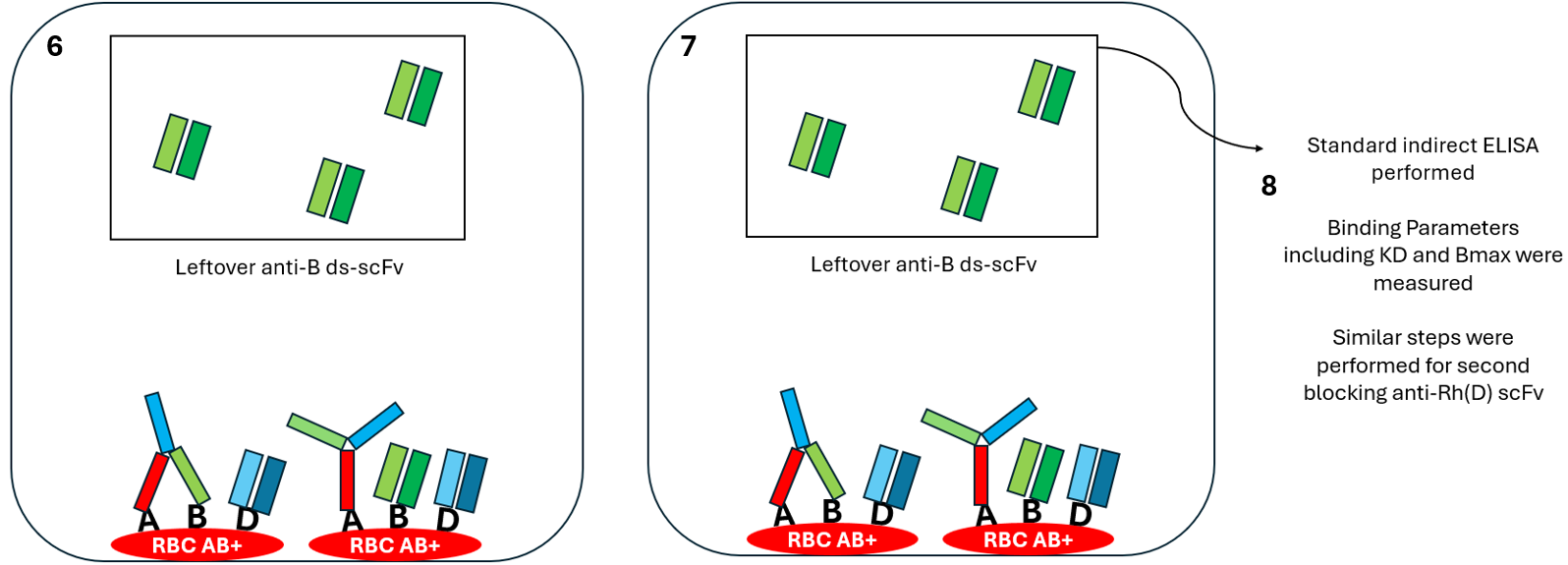
**

**Figure S5:** An overview of the process used for determining binding parameters K_D_ and B_max_ for binding order A→**B**→ **Rh(D)** using RBC-bound antigens.


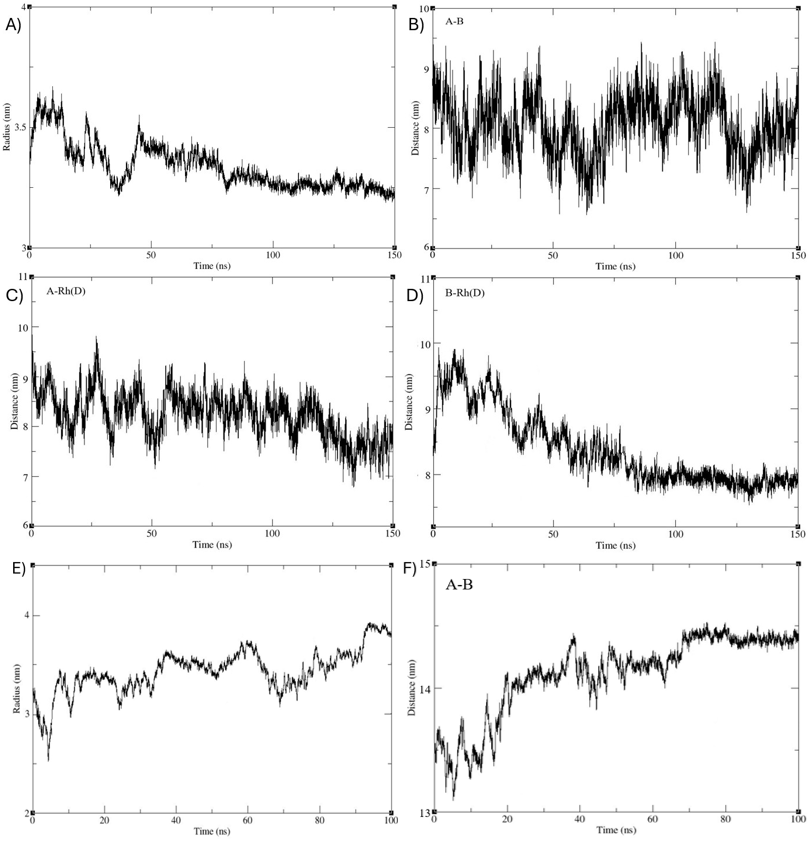


**Figure S6**: Graphs showing the radius of gyration and distances between binding sites of A-D) triabody-C1 and E-F) triabody-O1. A) radius of gyration of triabody-C1. B) distance between antigen binding sites of A and B of triabody-C1. C) distance between antigen binding sites of A and Rh(D) of triabody-C1. D) distance between antigen binding sites of B and Rh(D) of triabody-C1. E) radius of gyration of triabody-O1. F) distance between antigen binding sites of A and B of triabody-O1.


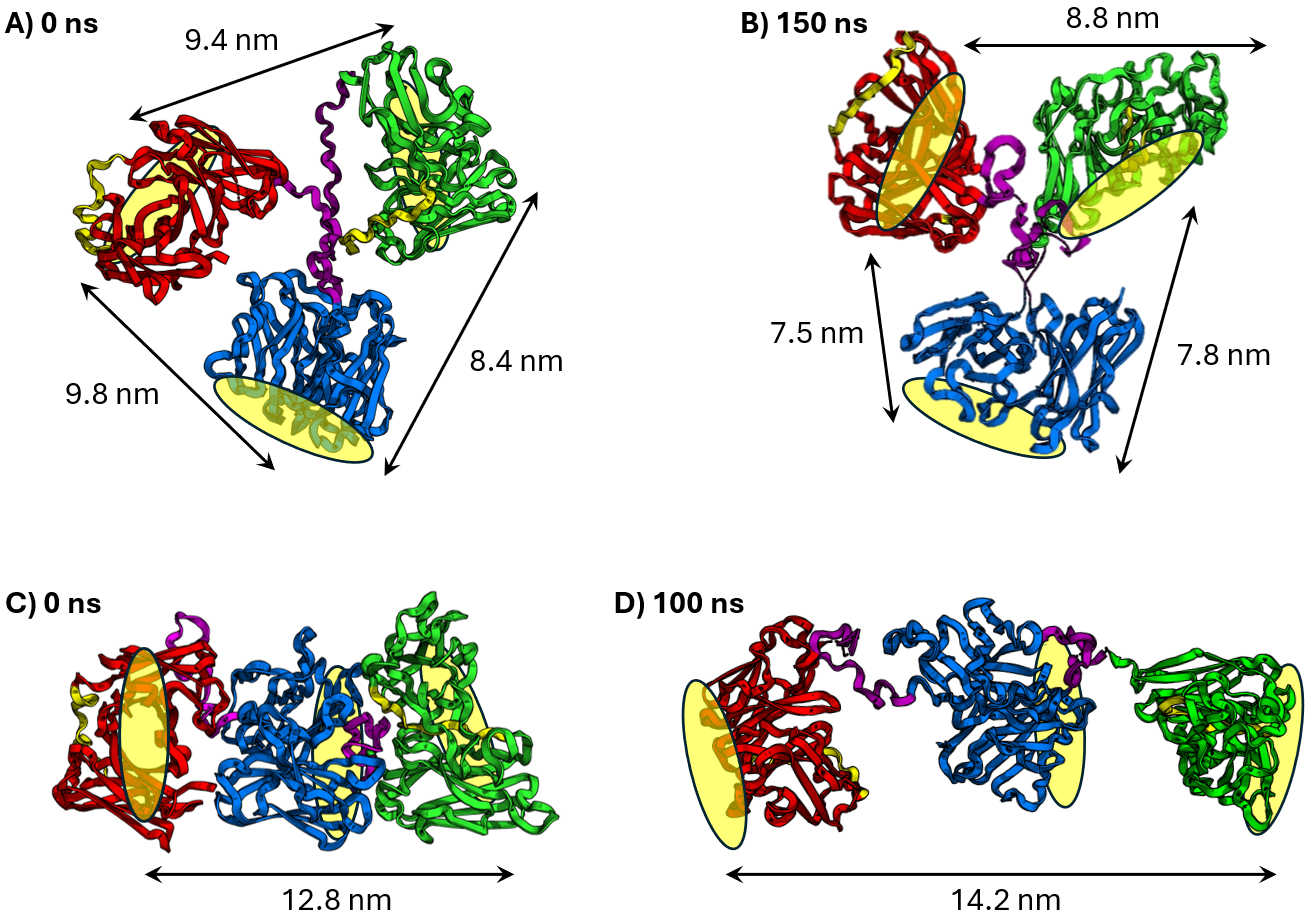


**Figure S7**: Snapshots from MD trajectories taken at the start and end of the simulation. A and B) Snapshots from the MD trajectory of triabody-C1 show the distances between antigen binding sites and domain flexibility. C and D) Snapshots from the MD trajectory of triabody-O1 show the distances between antigen binding sites and domain flexibility.


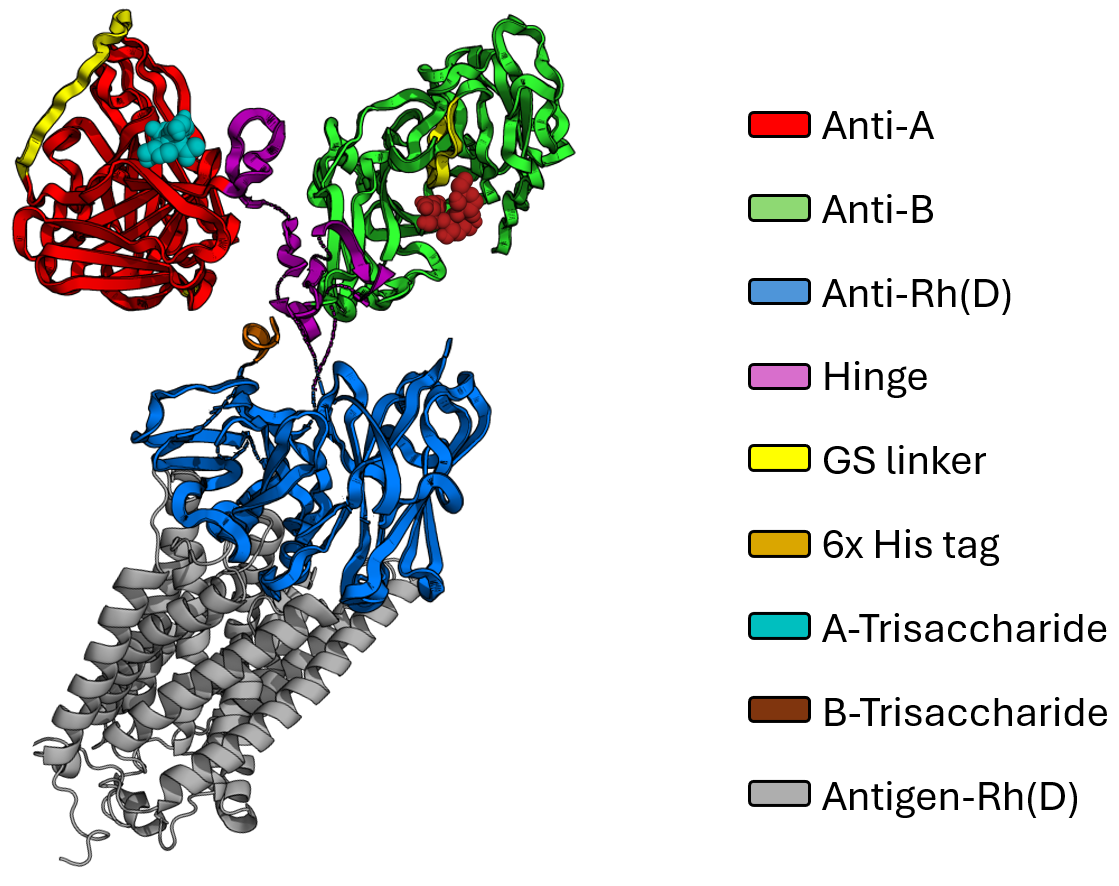


**Figure S8:** Snapshot from MD trajectory taken at the end of 150 ns simulation of binding sequence A→B→ Rh(D). All complexes including anti-A ds-scFv with antigen A, anti-B ds-scFv with antigen B and anti-Rh(D) Fv with antigen Rh(D) were stable throughout the simulations. Antigens A and B are shown as light blue and dark red spheres respectively. PyMOL 3.1 was used to generate high-resolution images.

**
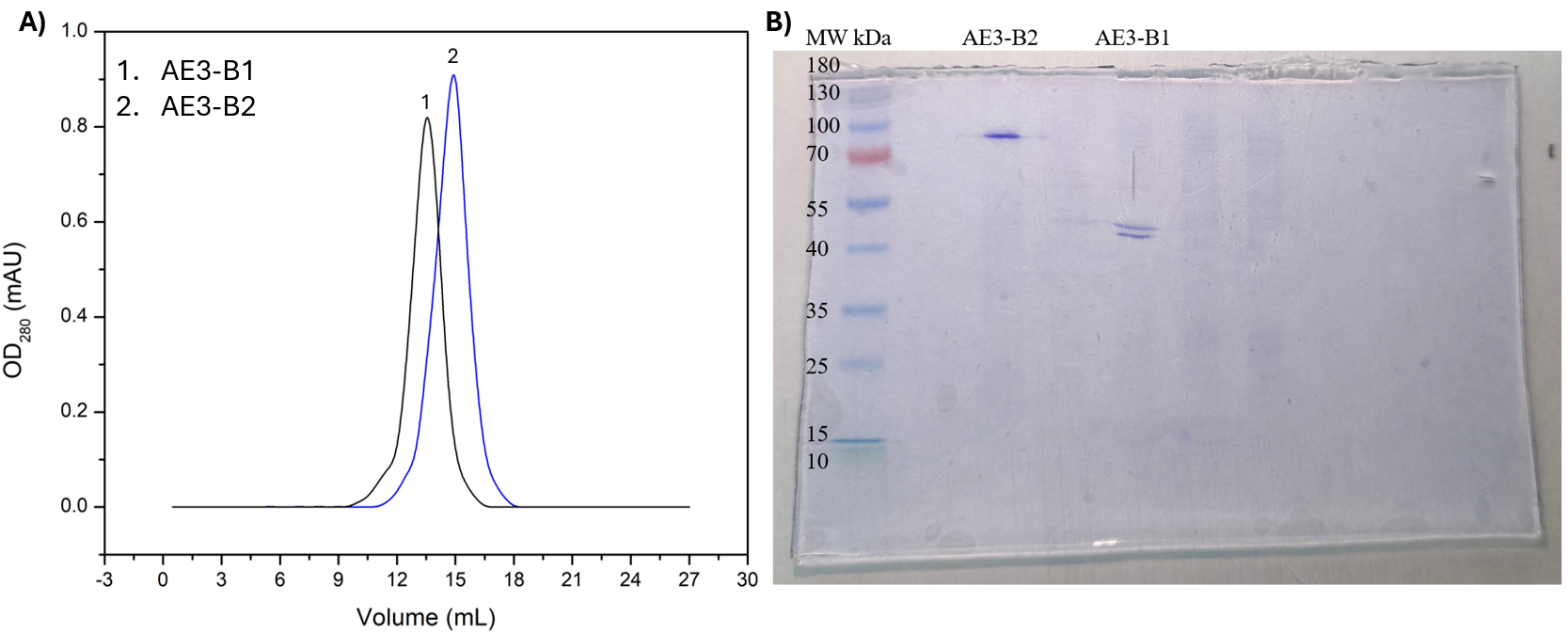
**

**Figure S9**: SEC graph showing peaks of triabodies in AE3-B1 (black) and AE3-B2 (blue) fractions. Non-reducing SDS PAGE showing bands at ~83kDa and ~45kDa of triabodies in AE3-B2 and AE3-B1 fractions respectively.


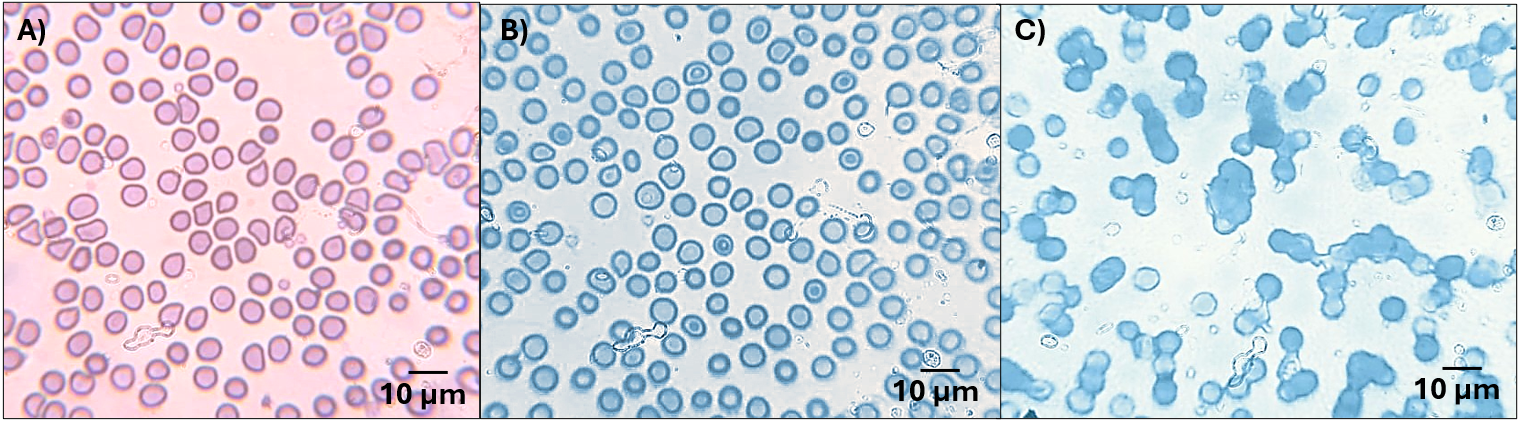


**Figure S10**: Micrographs of potentiator-enhanced hemagglutination assay. A) Giemsa-stained, non-hemagglutinated AB+ RBCs (control). B) TMB-stained, non-hemagglutinated AB+ RBCs in AE3-B2 triabody fraction. C) TMB-stained, hemagglutinated AB+ RBCs in AE3-B1 triabody fraction.


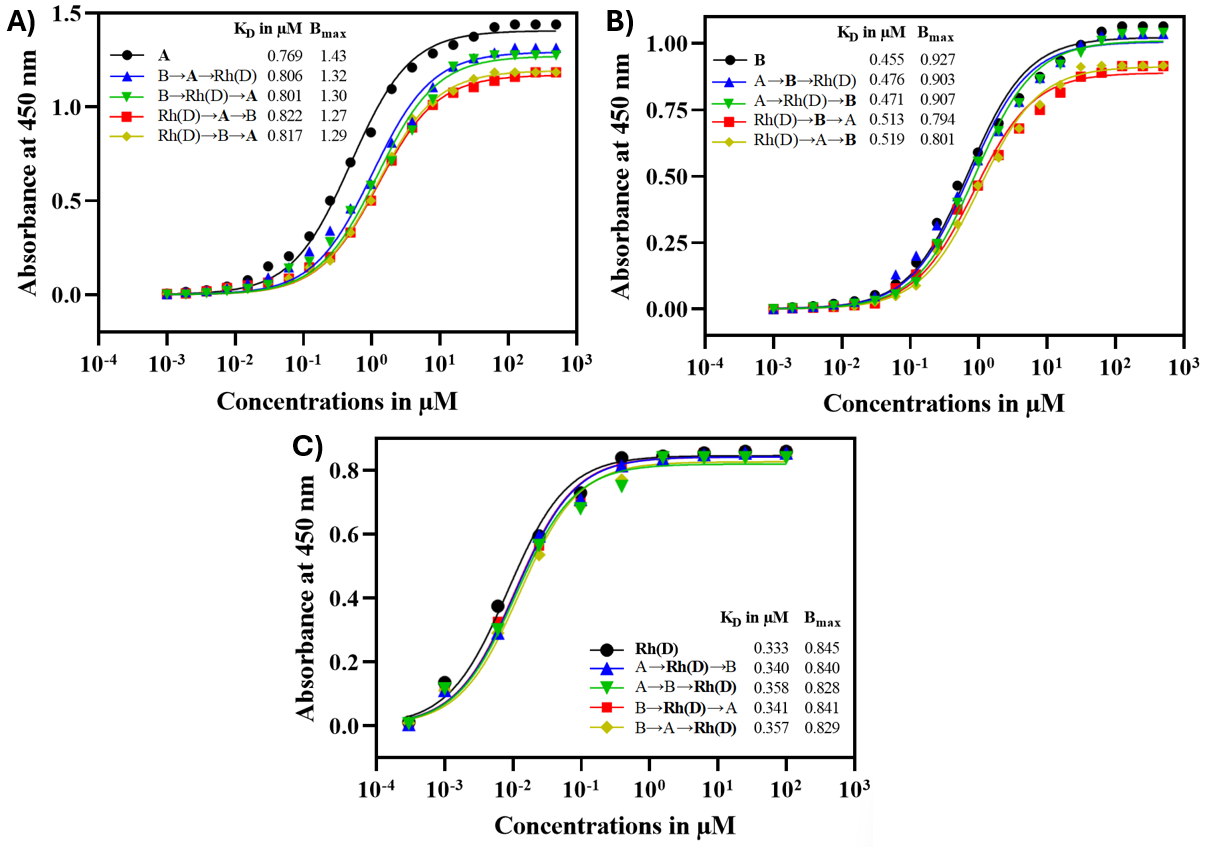


**Figure S11:** Binding behavior of the triabody toward RBC-bound blood group antigens. The binding parameters K_D_ and B_max_ are shown for A) anti-A ds-scFv, B) anti-B ds-scFv and C) anti-Rh(D) ds-scFv. The x-axis represents the ds-scFv log concentration (μM), and the y-axis represents the absorbance at 450 nm.


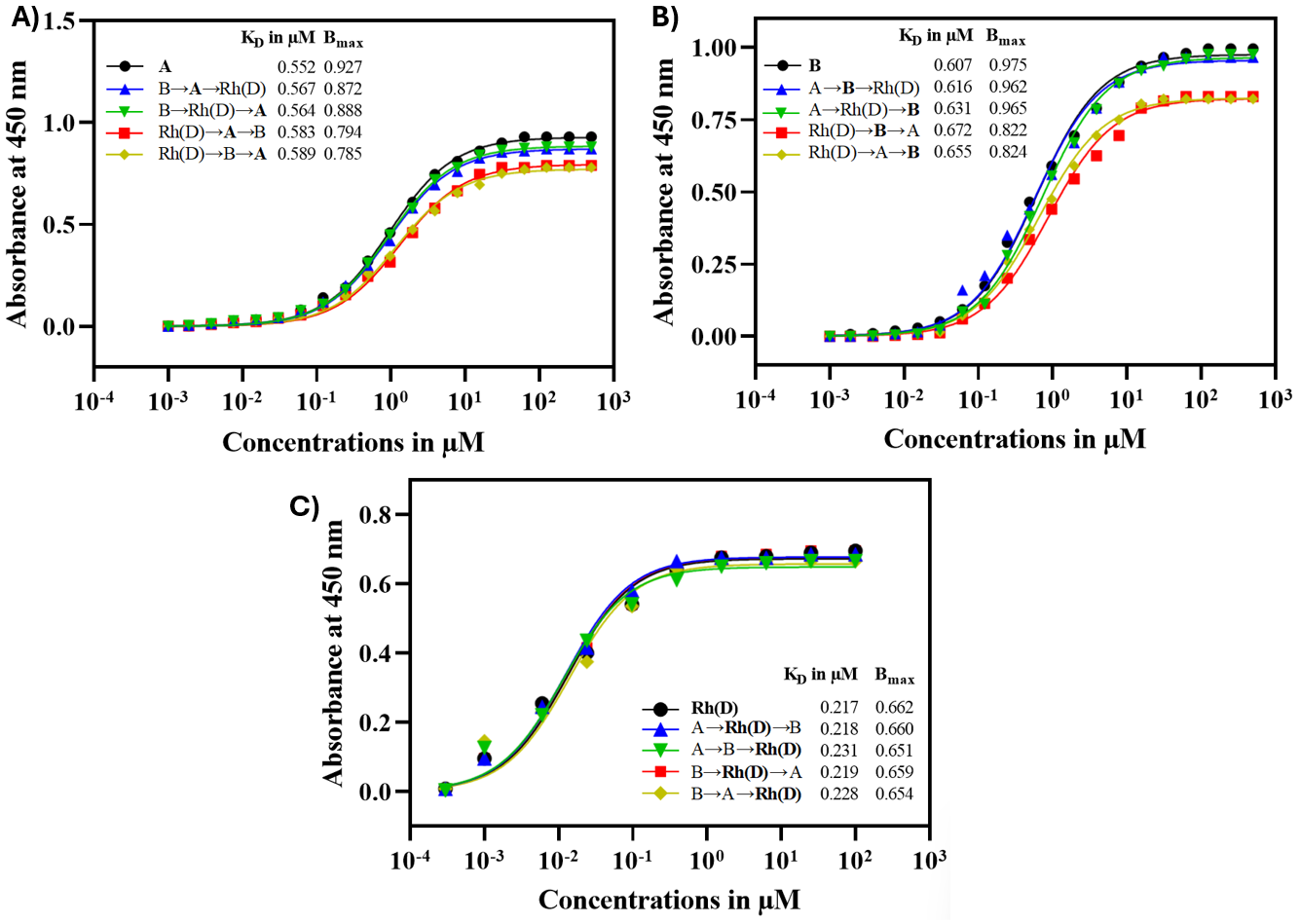


**Figure S12:** Binding behavior of the triabody toward RBC-bound blood group antigens. The binding parameters K_D_ and B_max_ are shown for A) anti-A ds-scFv, B) anti-B ds-scFv and C) anti-Rh(D) ds-scFv. The x-axis represents the ds-scFv log concentration (μM), and the y-axis represents the absorbance at 450 nm.

**
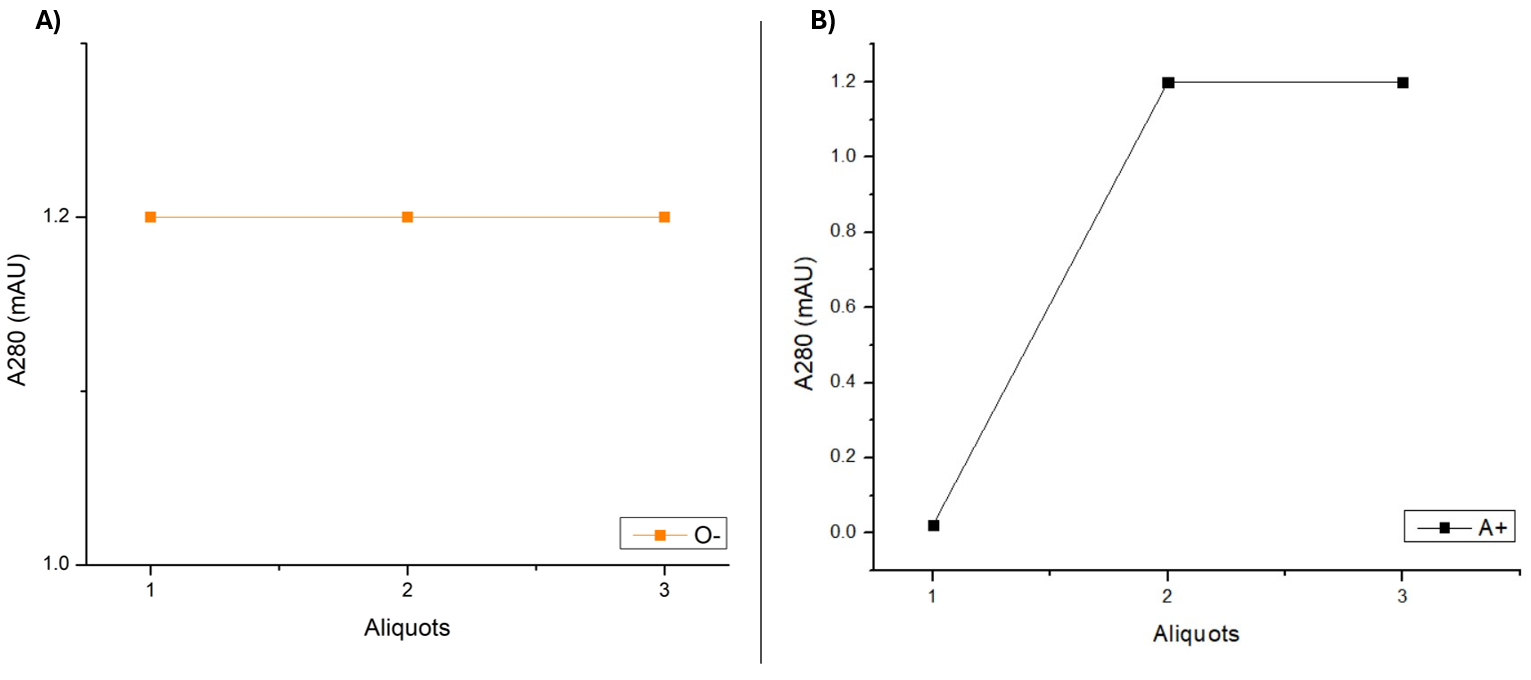

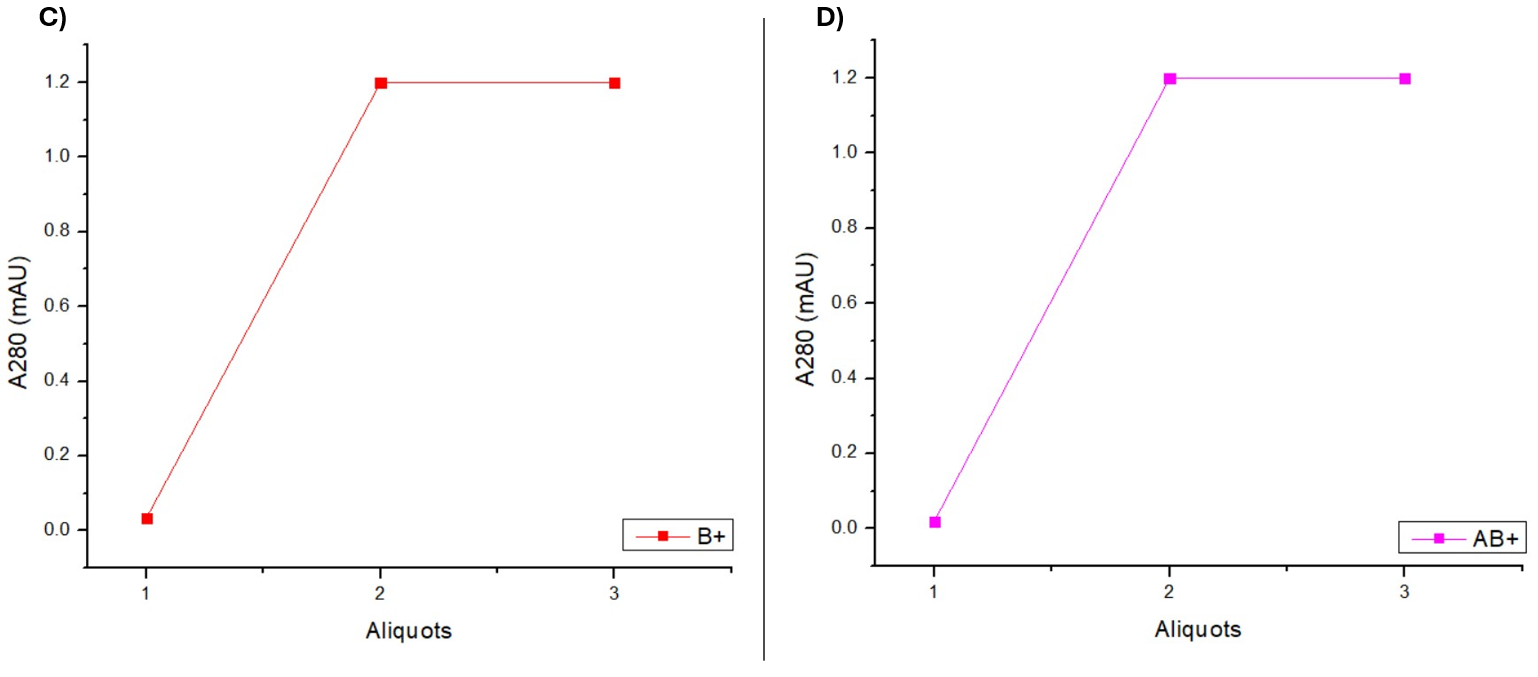

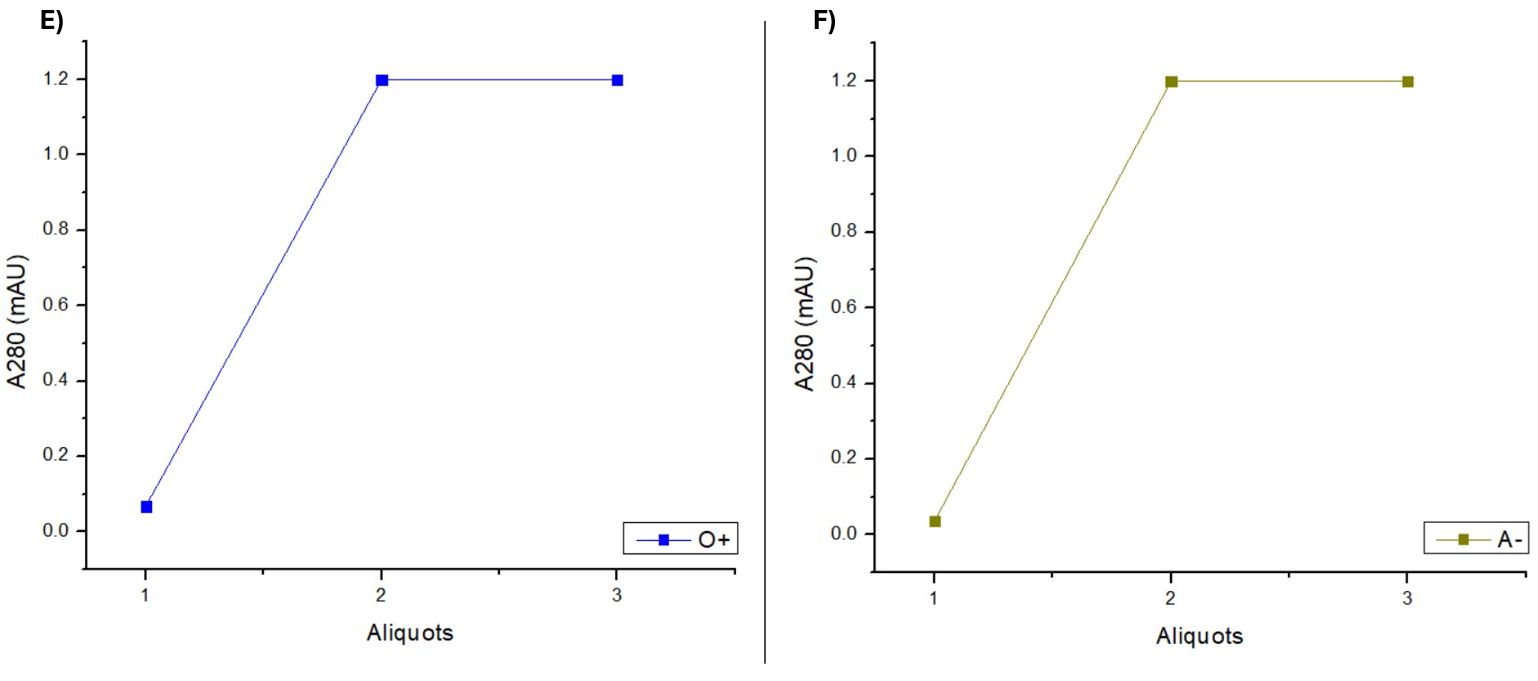
**
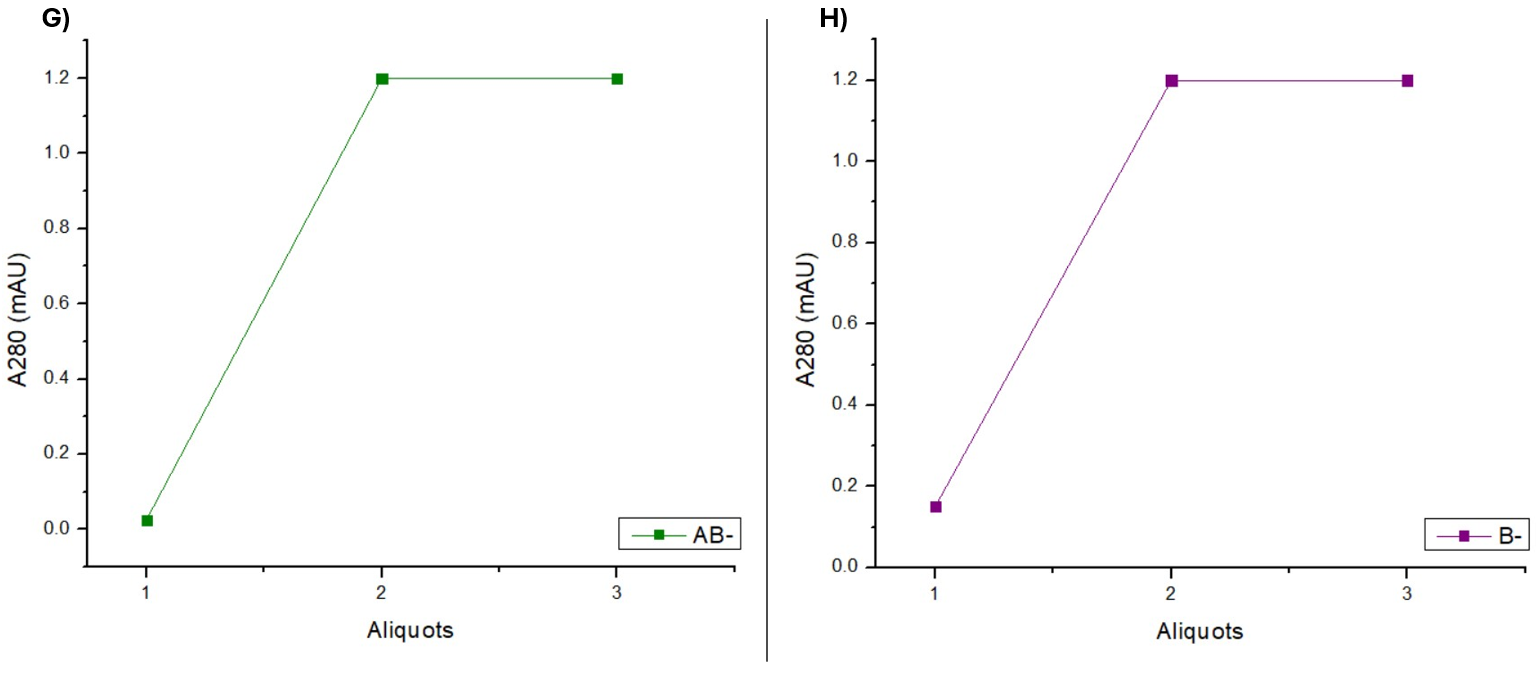


**Figure S13:** Graphs show the gradual decrease in the concentrations of triabody in the supernatant containing A) O-, B) A+, C) B+, D) AB+, E) O+, F) A-, G) AB- and H) B- RBCs.


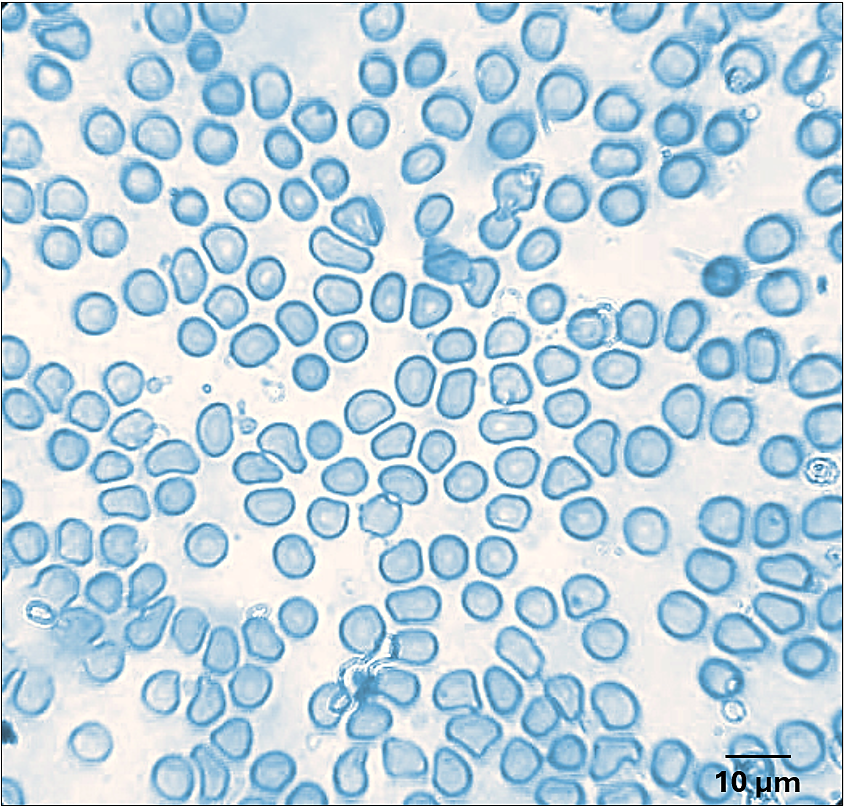


**Figure S14:** Micrograh showing non-hemagglutinated AB+ RBCs exposed to anti-A, anti-B and anti-Rh(D) IgM antibodies.

**
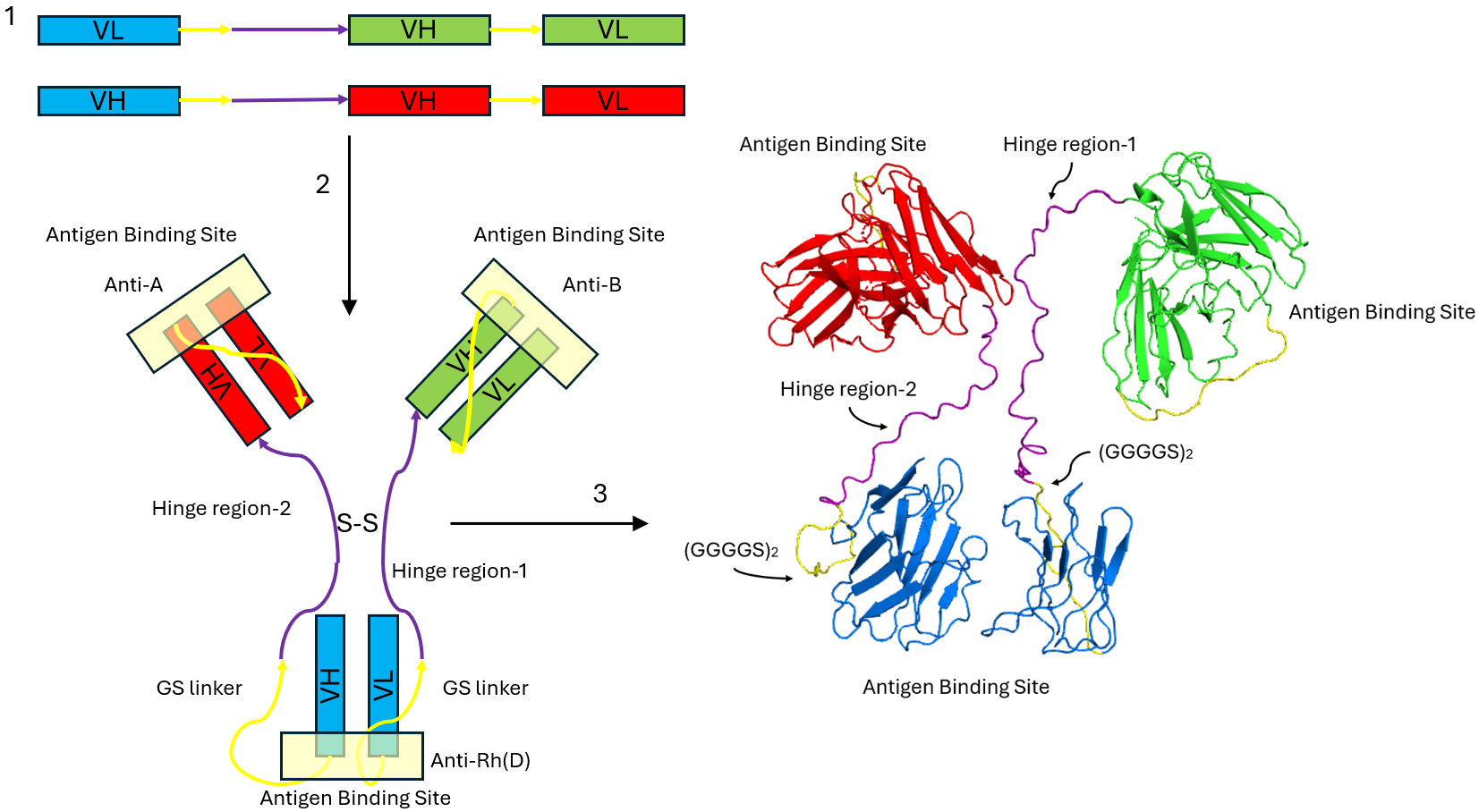
**

**Figure S15:** 1–2) Design and 3) model of the trifunctional triabody constructed from two modified fusion proteins of the second combination. To prevent steric hindrance and blocking of the anti-Rh(D) antigen-binding site by the hinge-linked interdomain disulfide bond, a flexible 10-amino-acid glycine-serine (GS) linker (yellow) was inserted between the hinge region (purple) and the variable chains (blue) of the anti-Rh(D) module. This linker increases conformational freedom, allowing the anti-Rh(D) domains to reorient and expose their antigen-binding surfaces in the final triabody conformation.


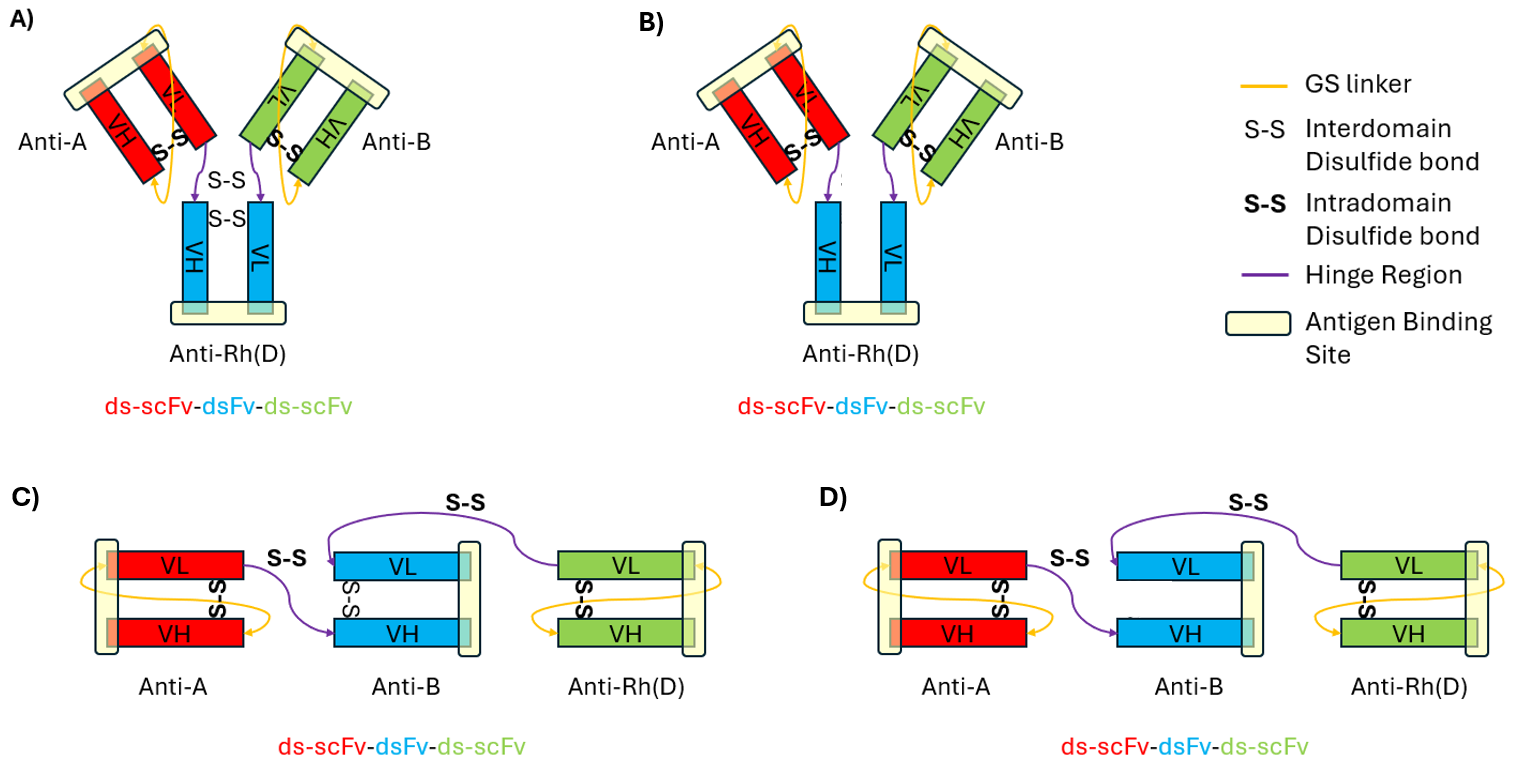


**Figure S16**: Design and structural configurations of triabodies. The figure illustrates the possible triabody architectures, including (linear) open and (compact) closed arrangements, with or without interdomain disulfide bonds, highlighting how structural differences can influence hemagglutination and functional behavior.
